# Different internal fixation methods for unstable distal clavicle fractures in adults: a systematic review and network meta-analysis

**DOI:** 10.1186/s13018-021-02904-6

**Published:** 2022-01-24

**Authors:** Yinglong Xu, Xiaobo Guo, Hui Peng, Hai Dai, Zonggui Huang, Jinmin Zhao

**Affiliations:** 1 Department of Trauma Orthopaedics and Hand Surgery, The Fifth Affiliated Hospital of Guangxi Medical University, Nanning, Guangxi China; 2 Research Centre for Regenerative Medicine, Department of Trauma Orthopaedics and Hand Surgery, The First Affiliated Hospital of Guangxi Medical University, Nanning, Guangxi China; 3 Department of Orthopaedics, Jincheng General Hospital, Jincheng, Shanxi China; 4 Department of Orthopaedics, Minzu Hospital of Guangxi Zhuang Autonomous Region, Nanning, Guangxi China

**Keywords:** Unstable distal clavicle fracture, CMS, Complications, Systematic review, Network meta-analysis

## Abstract

**Background:**

Surgical treatment is advised for unstable distal clavicle fractures (UDCFs). Various kinds of internal fixation methods have been used, but the best fixation is still controversial.

**Methods:**

We systematically searched all studies comparing postoperative outcomes of coracoclavicular (CC) reconstruction (TightRope, EndoButton, Mersilene tape, suture anchor or suture), fracture osteosynthesis (clavicular hook plate (HP), locking compression plate (LCP), Kirschner wire and tension band (KWTB), Kirschner wire (KW)), and a combination of the two methods (LCP + CC or KWTB + CC) for UDCF in PubMed, Web of Science Core Collection via Ovid, Embase, Cochrane Central Register of Controlled Trials (CENTRAL), and China Biology Medicine (CBM) databases up to September 16, 2021, with no language restrictions. A network meta-analysis (NMA) was conducted to integrate direct and indirect evidence and assess the relative effects of the internal fixation methods. The probability of being the best treatment was assessed by the surface under the cumulative ranking curve (SUCRA).

**Results:**

A total of 41 studies were included, involving 1969 patients and seven internal fixation methods. The NMA showed that LCP + CC fixation was associated with better efficacy (odds ratio (OR) 0.60, 95% CI 0.19–1.02, probability rank = 0.93) and fewer complications (odds ratio (OR) 0.22, 95% CI 0.09–0.51, probability rank = 0.69) than any other internal fixation method for UDCFs. The SUCRA probabilities of LCP + CC fixation were 98.6% for the Constant–Murley score and 93.9% for total complications.

**Conclusions:**

The results of this study indicate that LCP + CC appears to be the best internal fixation method for UDCF. Limited to the quality and quantity of the included studies, much larger and higher-quality RCTs are required to confirm these conclusions.

**Supplementary Information:**

The online version contains supplementary material available at 10.1186/s13018-021-02904-6.

## Introduction

Distal clavicle fractures (DCFs) are fractures located in the lateral third of the clavicle and account for 10–30% of clavicle fractures [1–3]. Neer [3] divided DCFs into five types based on the location of the fracture line in relation to the coracoclavicular (CC) ligament. Type II and type V fractures are unstable distal clavicle fractures (UDCFs), which often involve significant displacement caused by the loss of the coracoclavicular ligament from the proximal fragment and have a high rate of nonunion with conservative treatment [4, 5]. Surgical treatment is advocated for all UDCFs.

Surgical treatment for UDCFs is always a challenge for surgeons [6]. The difficulty of the treatment is due to the distal fragment of the fracture being too small for effective fixation, which can counteract the weight of the distal limb and the strong pull on the proximal fragment by the trapezius muscle. There are various fixation methods for UDCFs, including CC reconstruction [7, 8] (TightRope, EndoButton, Mersilene tape, suture anchors or sutures), fracture osteosynthesis (clavicular hook plate (HP) [9, 10], locking compression plate (LCP) [11], Kirschner wire and tension band (KWTB) [12], or Kirschner wire (KW) [13]), and a combination of the two methods (LCP + CC [14, 15] or KWTB + CC [16]). HPs are the most widely used internal fixators for UDCFs [17]. Clavicle HPs are inserted under the acromion through the distal hook and fixed proximally to the clavicle, forming a lever that maintains fracture reduction, which is consistent with the anatomy and biomechanics of the acromioclavicular joint. However, this internal fixation method also has complications such as subacromial osteolysis, rotator cuff injury, subacromial impingement and joint stiffness [18, 19]. Compared with HP fixation, arthroscopy-assisted CC reconstruction yields higher satisfaction from patients due to the minimally invasive surgical procedure with small wounds, minimal pain, good functional recovery, and no additional surgery is necessary to extract the internal implants [20]; LCP fixation also requires a smaller incision and significantly reduces the implant removal rate and postoperative complications [21]. CC ligament reconstruction may not be required when LCP is used to treat UDCFs [14]. There is still controversy regarding the optimal internal fixation method for UDCFs.

Several meta-analyses have compared the effectiveness and safety of different internal fixation methods for UDCFs [19, 22–26]. However, published meta-analyses did not include combined fixation methods (i.e., LCP + CC) and had a low level of evidence and high heterogeneity among the outcome parameters due to the small number of papers included. Therefore, we undertook a systematic review and network meta-analysis (NMA) of studies that compared the postoperative outcomes (incision size, operation time, blood loss, union time, Constant Murley Score (CMS), University of California at Los Angeles score (UCLAs), and CC distance (CCD)) and complications (total complications, implant-related complications, nonunion and delayed union, reoperation) of different internal fixation methods for UDCFs.

## Materials and methods

### Search strategies

We searched the PubMed, Web of Science Core Collection via Ovid, Embase, Cochrane Central Register of Controlled Trials (CENTRAL), and China Biology Medicine (CBM) databases to identify comparative studies of different internal fixation methods for UDCFs. All databases were searched from inception to September 16, 2021, with no language restrictions. The search strategy was as follows: [[(distal clavicle fracture) OR (lateral clavicle fracture)] AND (fracture fixation, internal)]. The search strategy for PubMed is described in Additional file 1: Appendix 1. An additional manual search of the reference lists of the included studies or any other relevant publications was also conducted independently by two investigators (Yinglong Xu and Hai Dai) to identify other eligible studies.

### Inclusion and exclusion criteria

We included comparable studies (cohort, case–control, and randomized controlled trials (RCTs)) that compared at least 2 kinds of internal fixation methods for acute UDCF in adults. Noncomparative studies, paediatric studies, or studies on acromioclavicular joint dislocation, nonunion, or shaft or medial fracture of the clavicle were excluded. Studies without any data on these outcomes (incision size, operation time, blood loss, union time, CMS, UCLAs, CCD, total complications, implant-related complications, nonunion and delayed union, and reoperation) were excluded. Pathological fractures and duplicate publications were also excluded.

### Study selection and data extraction

All records identified from the 5 electronic databases were downloaded and imported into EndNote X9 for literature management. Two reviewers (Yinglong Xu and Hai Dai) screened the literature independently. First, duplications were removed from the identified studies through automatic and manual checks, and then irrelevant studies were excluded by title and abstract screening. Finally, the full text of the rest of the potential studies was reviewed for definitive inclusion. Reasons for not eligible or excluded studies were documented. The differences between the two reviewers were resolved by consensus and discussion with a third author. (Zonggui Huang).

Two reviewers (Yinglong Xu and Xiaobo Guo) independently used standardized data extraction forms to extract the details of the included studies, including the baseline characteristics (author, location, study period, number of patients, age, sex, fracture type, follow-up period), elements for risk of bias evaluation, outcomes, and any statistics of interest. The data were cross-checked, and any discrepancies were resolved through discussions between the two investigators.

### Outcomes

The outcomes included (1) postoperative function assessment: CMS or UCLAs; (2) radiographic outcomes: CCD; (3) complications: total complications, implant-related complications (implant mispositioning, loss of reduction, peri-implant fractures, peri-anchor osteolysis, irritation and breakage of the implant), reoperation, and nonunion and delayed union). and (4)surgical outcomes: incision size (cm), operation time (min), blood loss (mL), union time (w).

### Risk of bias assessment

Two reviewers (Xiaobo Guo and Hui Peng) independently assessed the risk of methodological bias of the included studies. The Cochrane Risk of Bias tool [27] was employed for RCTs and includes the following domains: random sequence generation, allocation concealment, blinding, incomplete outcome data, and selective outcome reporting. The Risk Of Bias In Nonrandomized Studies—of Interventions (ROBINS-I) tool [28] was used for observational comparative studies, which considers six domains: within-study bias, reporting bias, indirectness, imprecision, heterogeneity, and incoherence. Assessments were displayed graphically with RevMan version 5.4 (Cochrane Collaboration) and the Confidence in Network Meta-Analysis (CINeMA) tool [29, 30]. Differences between the two reviewers were resolved by consensus and discussion with a third author (Hai Dai).

### Statistical analysis

NMA was performed according to the current Preferred Reporting Items for Systematic Review and Meta-Analyses Network Meta-Analyses (PRISMA-NMA) guidelines [31]. A network map was created to present the relationships between the different internal fixation methods. Odds ratios (ORs) and 95% confidence intervals (95% CIs) were used as summary statistics to present pooled estimates of dichotomous variables (complications), and mean deviations (MDs) and 95% CIs were used to report pooled estimates of continuous outcomes (Constant Murley Score (CMS), University of California at Los Angeles score (UCLAs), and CC distance (CCD), and surgical outcomes). The inconsistency assessment [32] comprised global inconsistency and local inconsistency. Global inconsistency was estimated by a design-by-treatment interaction model, and local inconsistency was estimated by the node-splitting method. *P* values < 0.05 were indicated statistical significance unless otherwise specified. Sensitivity analysis was performed to assess the transitivity. The surface under the cumulative ranking curve (SUCRA) was used to rank the effectiveness or safety of internal fixation methods by estimating the probability of a method yielding the best fixation. A larger SUCRA was considered a much better fixation. The predictive interval was assessed and graphed to confirm whether relative treatment effects would work in other populations. Network funnel plot and Egger’s test were generated to evaluate potential publication bias. *P* values < 0.05 were indicated high risk of publication bias. The NMA was conducted in Stata 15.0 (Stata, College Station, Texas, USA.). The confidence for the results comparing different internal fixation methods was estimated with the Confidence in Network Meta-Analysis (CINeMA) tool, a web application that simplifies the assessment of confidence in findings from NMA.

## Results

### Identification of eligible studies

**Fig. 1:**
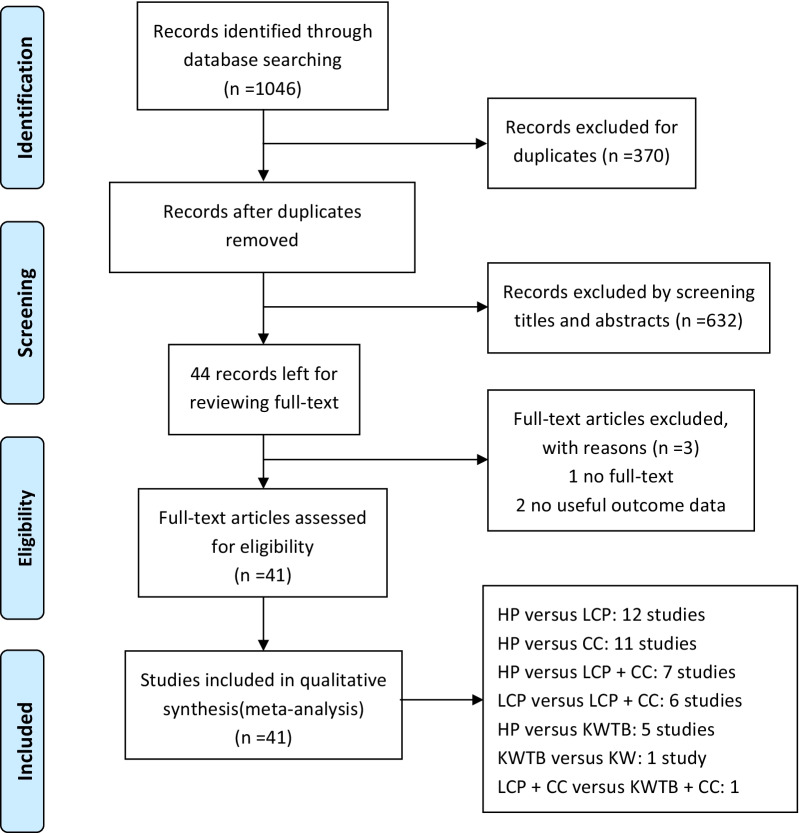
Flowchart of the literature selection

We found 1046 articles through the electronic database search. After removing duplicate studies, 676 studies underwent title and abstract review. A total of 632 studies were excluded due to being noncomparative studies, paediatric studies, or studies on acromioclavicular joint dislocation, nonunion, or shaft or medial fracture of the clavicle. Forty-four studies underwent full text review and data extraction, and 41 studies [14–16, 20, 21, 33–68] were included in the network meta-analysis (NMA). The PRISMA flowchart of the study selection procedure is presented in Fig. 1.

### Characteristics of the included studies

The baseline characteristics of the included studies are presented in Table 1. Of the 41 included studies, 28 [15, 20, 33–36, 39, 42, 44–48, 53–68] were from China, 3 [14, 21, 52] were from the USA, 3 [37, 38, 43] were from Germany, 2 [40, 41] were from Finland and 1 each was from Korea [51], Australia [33], Turkey [16], Morocco [49], and the Netherlands [50]. There were 1969 Neer type II (1642/1969, 83.4%; type II B, 709/1969, 36%) and unclear type (type II or type V, 327/1969, 16.6%) distal clavicle fractures that were fixed with HP (hook plate, 923/1969, 46.9%), LCP (locking compression plate, 384/1969, 19.5%), CC (coracoclavicular reconstruction, 255/1969, 13.0%), LCP + CC (combination of locking compression plate and coracoclavicular reconstruction, 260/1969, 13.2%), KWTB (Kirshner wire and tension band, 123/1969, 6.2%), KWTB + CC (combination of Kirshner wire and tension band and coracoclavicular reconstruction, 10/1969, 0.5%), or KW (Kirshner wire, 14/1969, 0.7%). Twelve studies [21, 34, 36, 38, 47, 50, 52, 53, 56, 58, 60, 65] provided data on HP versus LCP (number of patients: 274 vs. 286), 11 studies [20, 35, 40, 44, 48, 52, 58, 59, 62, 63, 67] provided data on HP versus CC (number of patients: 281 vs. 255), 7 studies [33, 42, 43, 51, 64, 66, 68] provided data on HP versus LCP + CC (number of patients: 178 vs. 148), 6 studies [14, 15, 37, 39, 54, 61] provided data on LCP versus LCP + CC (number of patients: 98 vs. 98), 5 studies [45, 46, 49, 57] provided data on HP versus KWTB (number of patients: 190 vs. 108), 1 study [55] provided data on KWTB versus KW (number of patients: 15 vs. 14), and 1 study [16] provided data on LCP + CC versus KWTB + CC (number of patients: 14 vs. 10).The included studies were published from 2002 to 2021, and the research period of the included studies was from 1988 to 2019. The proportion of women ranged from 4.7 to 70.1%. The mean age and postoperative follow-up ranged from 31.7 to 55.2 years and 6 to 76.2 months, respectively.

**Table 1:** Baseline characteristics of included studies

References	Country	Study design	Duration	Fixation methods	No. of patients	Age (years)		Gender (M/F)		Neer type	Follow-up (months)		Outcomes
Wang et al. [56]	China	Retrospective	2014.8–2018.4	HP/LCP	64 (33/31)	37.0 ± 10.0	38.8 ± 8.6	21/12	19/12	II	12		1, 2, 4, 5, 6, 7, 8
Li et al. [47]	China	Retrospective	2015.1–2017.5	HP/LCP	44 (26/18)	51.7 ± 17.1	55.2 ± 16.3	16/10	11/7	II, V	17.2 (13–19)		1, 4, 6, 7, 8
Chen et al. [21]	USA	Retrospective	2009–2019	HP/LCP	31 (12/19)	40 ± 15	44 ± 13	10/2	15/4	II, V	31 ± 31	40 ± 36	4
Ochen et al. [50]	Netherlands	Retrospective	2011.1–2016.6	HP/LCP	67 (19/48)	42 ± 17	43 ± 12	13/6	41/7	II, V	31.3 ± 16.3	40.0 ± 18.0	4
Singh et al. [52]	USA	Retrospective	2010.1–2012.9	HP/LCP	53 (16/37)	31.7 ± 16.0	36.2 ± 15.9	10/6	27/10	II, V	NR		4
Xiong et al. [58]	China	Retrospective	2001–2010	HP/LCP	30 (25/5)	46.5 ± 15.8	38.0 ± 14.7	34/24		II	77.4 ± 34.6	76.2 ± 72.4	4, 5, 6, 7
Erdle et al. [38]	Germany	Retrospective	2004–2016	HP/LCP	32 (19/13)	44.3 ± 14.9	43.7 ± 13.7	NR		IIB	54.9 ± 19.8	53.3 ± 19.6	1, 4
Cai et al. [34]	China	Prospective	2011.3–2013.3	HP/LCP	40 (20/20)	33.3 ± 7.5	32.9 ± 6.6	15/5	16/4	II	NR		2, 4, 6, 7
Zhu et al. [65]	China	Retrospective	2008.1–2011.12	HP/LCP	46 (26/20)	40.3 (20–57)		31/15		II	NR		1, 4, 8
Zhang et al. [60]	China	Retrospective	2007.2–2010.11	HP/LCP	66 (30/36)	41.1 ± 10.3	42.5 ± 10.7	17/13	20/16	II	27.2 ± 6.1	28.6 ± 6.2	1, 4
Tan et al. [53]	China	Retrospective	2007–2009	HP/LCP	42 (23/19)	41.8 ± 11.1	40.5 ± 9.6	15/8	13/6	II	22.1 ± 9.3	22.4 ± 10.1	2, 4, 6, 8
Dai et al. [36]	China	Retrospective	2008.1–2009.1	HP/LCP	45 (25/20)	35.6 (21–46)	36.5 (24–51)	17/8	13/7	II	15.6 (12–24)		4
Wang et al. [67]	China	Retrospective	2014.10–2016.10	HP/CC	50 (25/25)	44.8 ± 13.9	43.3 ± 14.3	15/10	19/6	IIB	12.7 ± 4.0		4, 5, 6, 7
Wu et al. [20]	China	Prospective	2015.10–2018.2	HP/CC	26 (14/12)	48.9 ± 16.5	44.8 ± 10.3	8/6	9/3	II	18.9 ± 3.5	17.0 ± 4.2	1, 2, 4, 5, 6, 7
Singh et al. [52]	USA	Retrospective	2010.1–2012.9	HP/CC	37 (16/21)	31.7 ± 16.0	43.8 ± 19.6	10/6	16/4	II, V	NR		4
Xiang et al. [62]	China	Retrospective	2009.1–2015.10	HP/CC	66 (34/32)	40.2 ± 11.4	41.8 ± 12.7	26/8	22/10	IIB	30.5 ± 7.1	29.1 ± 5.6	1, 4, 6, 8
Xiong et al. [58]	China	Retrospective	2001–2014	HP/CC	53 (25/28)	46.5 ± 15.8	41.9 ± 13.5	34/24		II	77.4 ± 34.6	35.6 ± 13.9	4, 5, 7
Hsu et al. [44]	China (TW)	Retrospective	2010–2014	HP/CC	72 (49/23)	47.9 ± 19.5	42.4 ± 15.9	29/20	16/7	II, V	13.3 ± 2.5	14.2 ± 1.9	1, 2, 4, 8
Xu et al. [59]	China	Retrospective	2013.4–2015.6	HP/CC	42 (22/20)	33.8 ± 10.7	37.7 ± 9.9	14/8	11/9	II	7.2 (6–12)		4, 6, 7
Flinkkilä et al. [40]	Finland	Retrospective	2007–2012	HP/CC	40 (19/21)	45 ± 13	39 ± 14	13/6	20/1	II	62 ± 21	32 ± 16	1, 4
Lu et al. [48]	China	Retrospective	2009.5–2010.5	HP/CC	40 (19/21)	34.3 ± 1.8	33.2 ± 1.5	11/8	11/10	IIB	12–24		1, 4, 8
Chen et al. [35]	China (TW)	Retrospective	2004.12–2010.8	HP/CC	68 (28/40)	48.3 (28–78)	43.2 (18–75)	16/12	28/12	IIB	37.4 (24–68)	38.2 (24–64)	4
Wu et al. [63]	China	Retrospective	2008.1–2010.3	HP/CC	42 (30/12)	37.5 ± 2.8	36.8 ± 1.5	22/8	9/3	II	12.6 (3–18)		4, 5, 6, 7
Seo et al. [51]	Korea	Retrospective	2011.1–2019.3	HP/LCP + CC	82 (54/28)	43.9 ± 15.8	45.6 ± 18.2	35/19	18/10	IIB	18.7 ± 4.2	15.6 ± 2.1	1, 4, 6, 8
Hu et al. [68]	China	Retrospective	2011.4–2018.5	HP/LCP + CC	42 (23/19)	41.6 ± 13.2		14/9	12/7	II	17.5 ± 4.7		1, 3, 4, 6, 7, 8
Zeng et al. [64]	China	Retrospective	2012–2016	HP/LCP + CC	39 (18/21)	43.4 ± 13.9	41.7 ± 14.0	7/11	11/10	IIB	24.6 ± 6.6		1, 3, 4, 6, 7, 8
Helfen et al. [43]	Germany	Retrospective	2011.11–2015.11	HP/LCP + CC	41 (21/20)	39.7 ± 14.6	53 ± 17.5	NR		IIB	NR		1, 4
Lin et al. [66]	China	Retrospective	2012.1–2015.1	HP/LCP + CC	65 (32/33)	41.2 ± 9.9	42.6 ± 10.4	18/14	21/12	IIB	15.2 ± 2.1	14.6 ± 5.2	1, 4, 8
Gao et al. [42]	China	Retrospective	2010.8–2013.8	HP/LCP + CC	42 (20/22)	45.6 ± 12.4	44.2 ± 11.6	11/9	14/8	IIB	16.8 (10–24)		1, 3, 4, 8
Bhatia et al. [33]	Australia	Retrospective	2005–2008	HP/LCP + CC	15 (10/5)	34.5 (20–57)	13/2		IIB	26.1 (12–40)		4	
Dey Hazra et al. [37]	Germany	Retrospective	2014–2017	LCP/LCP + CC	31 (14/17)	43 ± 15	43 ± 17	9/5	9/8	IIB	29 ± 12.4	18 ± 7.1	1, 3, 4, 6
Salazar et al. [14]	USA	Retrospective	2009–2019	LCP/LCP + CC	23 (16/7)	42 ± 13	45 ± 13	14/2	5/2	II, V	28 ± 34	47 ± 39	4
Zhang et al. [61]	China	Retrospective	2013.1–2015.1	LCP/LCP + CC	40 (20/20)	37.3 ± 10.6	40.6 ± 12.6	16/4	15/5	IIB	30.1 ± 6.7		1, 3, 4, 5, 6, 7, 8
Xu et al. [15]	China	Retrospective	2010.2–2017.1	LCP/LCP + CC	34 (16/18)	50.7 ± 17.7	45.5 ± 14.4	9/7	12/6	IIB	16.0	17.5	1, 4, 8
Tang et al. [54]	China	Retrospective	2013.2–2017.1	LCP/LCP + CC	40 (22/18)	45.4 (26–65)	43.3 (24–61)	10/12	11/7	IIB	16.3 (12–27)		1, 4, 6
Fan et al. [39]	China	Retrospective	2013.1–2015.1	LCP/LCP + CC	28 (10/18)	40.2 ± 12.6	36.9 ± 11.0	7/3	14/4	II	21.8 ± 7.7	18.4 ± 3.6	1, 2, 3, 4, 5, 6, 7, 8
Mechchat et al. [49]	Morocco	Prospective	2009–2013	HP/KWTB	26 (14/12)	34.2 ± 13.7	29 ± 13.7	8/6	5/7	II	12		1, 4, 8
Wu et al. [57]	China (TW)	Retrospective	2007.1–2010.6	HP/KWTB	116 (92/24)	49.3 ± 15.5	50.7 ± 17.6	55/37	17/7	II	22.8 ± 2.2	25.7 ± 2.8	1, 4
Hsu et al. [45]	China (TW)	RCT	2005.2–2008.11	HP/KWTB	65 (35/30)	43.2 ± 12.8	41.3 ± 13.6	23/12	21/9	II	6		4
Lee et al. [46]	China (TW)	Retrospective	2000–2007	HP/KWTB	52 (32/20)	43.4	35.9	18/14	9/11	II	24.3	29.8	1, 4
Flinkkilä et al. [41]	Finland	Retrospective	1988–1999	HP/KWTB	39 (17/22)	43 (18–71)	35 (17–68)	16/1	16/6	II	24	74.4	4
Tsuei et al. [55]	China (TW)	Retrospective	2002–2008	KW/KWTB	29 (14/15)	39.2 ± 15.0	39.3 ± 14.1	9/5	11/4	II	8.62 (6–20)		2, 4, 6, 8
Seyhan et al. [16]	Turkey	Retrospective	2000–2011	KWTB + CC/LCP + CC	24 (10/14)	36.1 (20–55)		6/4	10/4	IIB	NR		1, 4

*RCT* randomized controlled trail, *HP* hook plate, *LCP* locking compression plate, *CC* coracoclavicular reconstruction, *LCP + CC* combination of locking compression plate and coracoclavicular reconstruction, *KWTB* Kirshner wire and tension band, *KWTB + CC* combination of Kirshner wire and tension band and coracoclavicular reconstruction, *KW* Kirshner wire, *NR* not reported

Outcomes: 1, Constant Murley Score (CMS); 2, University of California at Los Angeles (UCLA) score; 3, coracoclavicular distance (CCD); 4, complications; 5, incision; 6, operative time; 7, blood loss; 8, union time

### Risk of bias assessment

Among the 41 studies included in this meta-analysis, one randomized controlled trail (RCT) [45] had a high risk of bias, as evaluated by the Cochrane Risk of Bias tool. All 40 comparative studies [14–16, 20, 21, 33–44, 46–68] were at high risk of bias, as evaluated by the Risk Of Bias In Non-randomized Studies—of Interventions (ROBINS-I) tool. The results of the evaluation of the exposure to methodological bias for RCTs and nonrandomized studies are shown in Additional file 2: Fig. S1(B, A), respectively. Additional file 2: Fig. S1C shows the average risk of bias contribution for each comparison within the network.

### Constant Murley Score (CMS)

**Fig. 2:**
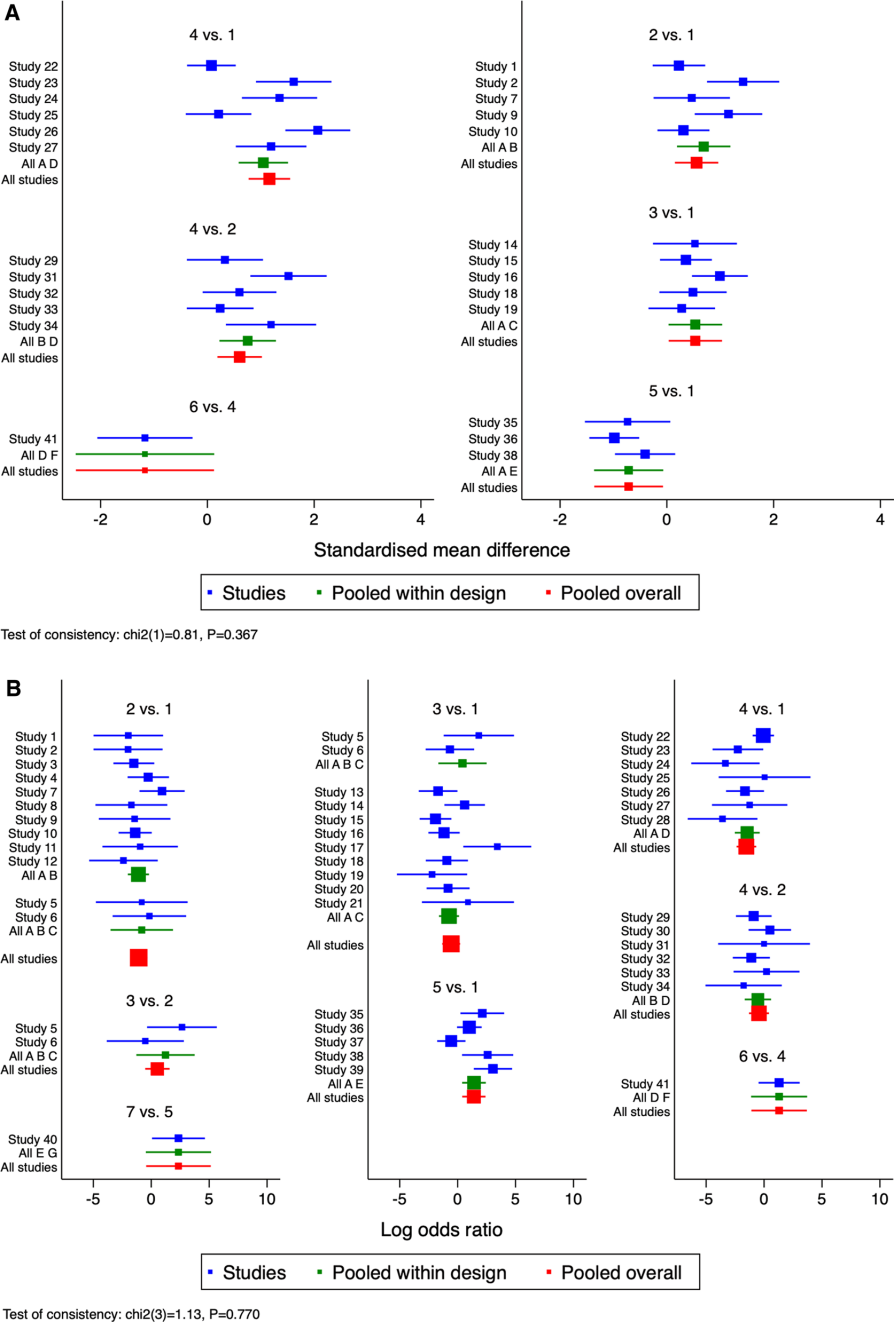
Forest plots of the meta-analysis of different internal fixation methods for UDCFs. **A** CMS; **B** Total complications. 1, HP; 2, LCP; 3, CC; 4, LCP + CC; 5, KWTB; 6, KWTB + CC; 7, KW

**Fig. 3:**
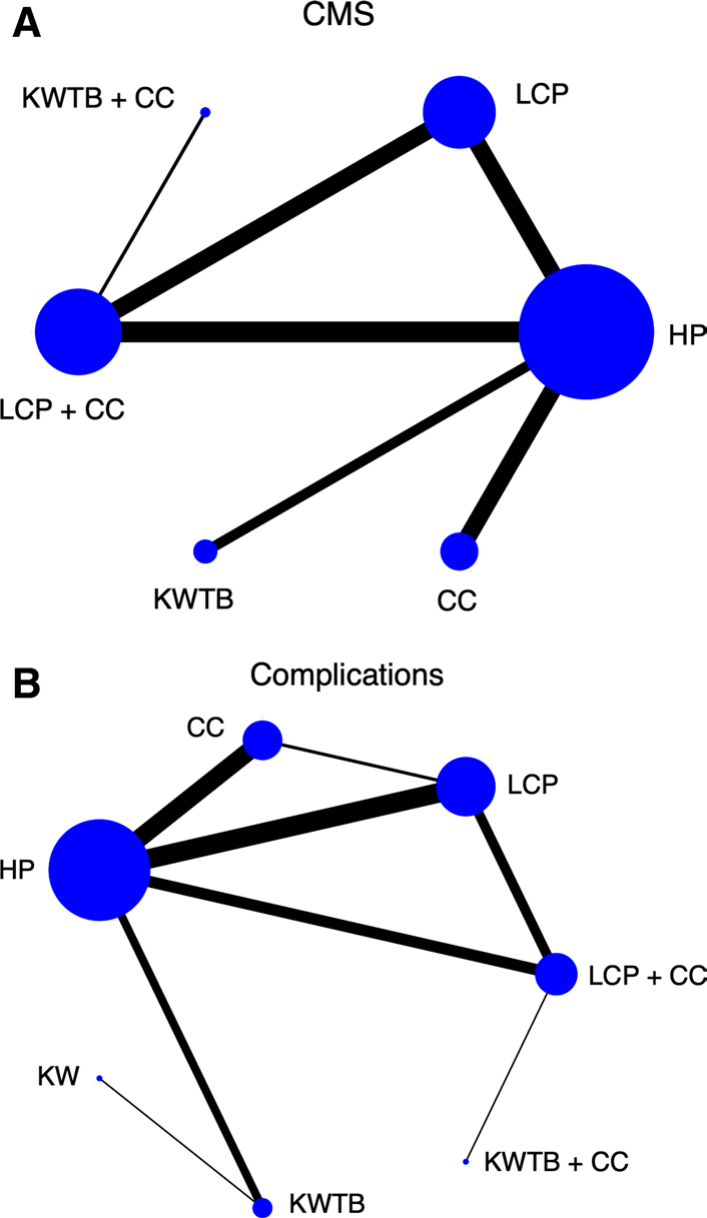
Network meta-analysis maps of CMS and total complications. **A** CMS; **B** Total complications. Each node represents an intervention, and the size of the node is proportional to the number of patients assigned to the intervention. The lines indicate direct comparisons between nodes, and the size of the line is proportional to the number of trials comparing each pair of nodes

**Fig. 4:**
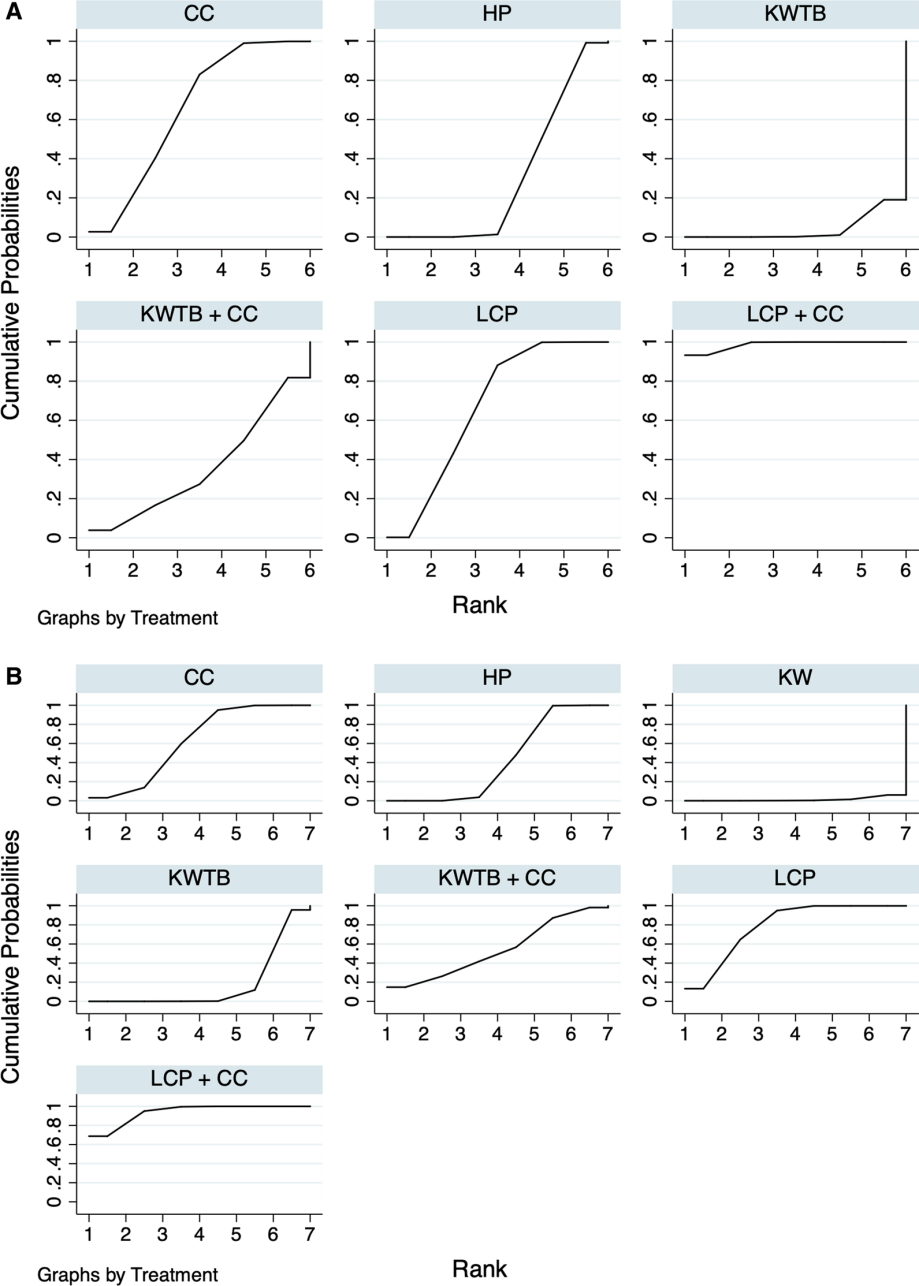
Rankogram of different internal fixation methods for CMS and total complications. **A** CMS; **B** Total complications

**Fig. 5:**
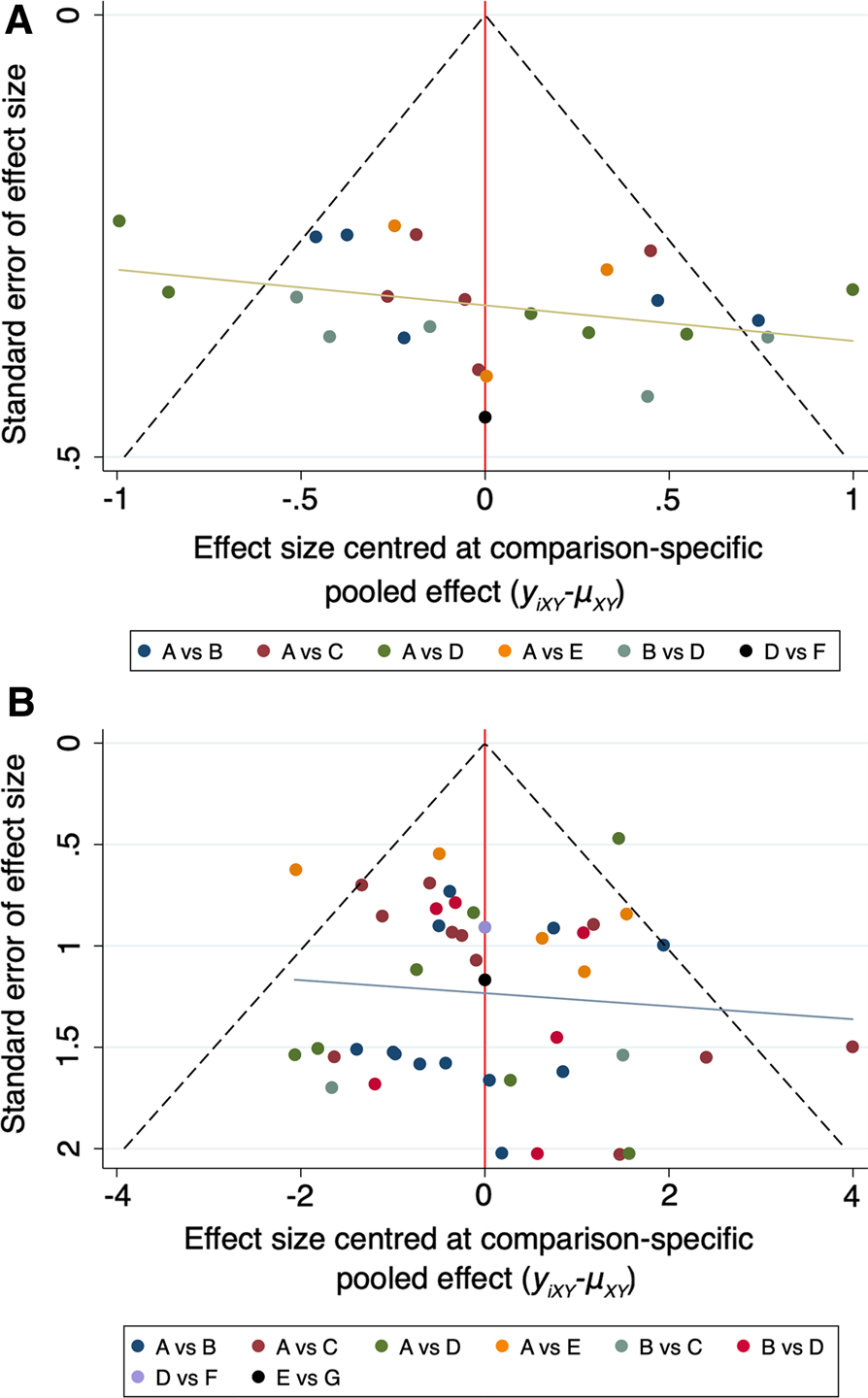
Network meta-analysis funnel plots for the assessment of publication bias of the included studies. **A** CMS; **B** Total complications. **A** HP, **B** LCP, **C** CC, **D** LCP + CC, **E** KWTB, **F** KWTB + CC, **G** KW

The NMA of the CMS comprised 25 studies [15, 16, 20, 37–40, 42–44, 46–49, 51, 54, 56, 57, 60–62, 64–66, 68] with 25 direct comparisons of 6 different internal fixation methods (HP, LCP, CC, LCP + CC, KWTB, and KWTB + CC) (Fig. 2A). The network map is shown in Fig. 3A. The results of the NMA are presented in Table 2, which shows that the combinations of LCP and CC, CC, and KWTB + CC were much more effective for UDCFs compared with LCP, followed by HP and KW fixation. LCP + CC fixation had a significantly higher CMSs of 0.60 (95% CI 0.19–1.02), 1.16 (95% CI 0.77–1.55), and 1.88 (95% CI 1.12–2.63) when compared with LCP, HP, and KWTB, respectively, but no measurable difference was present when compared with CC and KWTB + CC. For ranking of the best treatment, LCP + CC fixation was the first, with an SUCRA of 98.6 (Fig. 4A) and a probability of being the best treatment of 93.3%. There was no measurable inconsistency (*p* = 0.367) within the NMA. In addition, the prediction intervals were assessed and presented with a graph (Additional file 3: Fig. S5A). The funnel plot and Egger's test did not indicate any risk of publication bias (Fig. 5A, *P* = 0.171). The confidence of most of the comparisons of interest was graded as low (Additional file 4: Table S1).

**Table 2:** League table of different internal fixation comparisons of CMS, UCLAs, and CCD for UDCF

**CMS** MD (95% CI)					
**LCP + CC**	0.77 (0.27, 1.28)^a^	NR	NR	1.09 (0.39, 1.79)^a^	NR
0.60 (0.19, 1.02)*	**LCP**	NR	NR	0.70 (0.23, 1.17)^a^	NR
0.63 (− 0.01, 1.26)	0.02 (− 0.62, 0.67)	**CC**	NR	0.55 (0.29, 0.82)	NR
1.17 (− 0.12, 2.46)	0.56 (− 0.79, 1.92)	0.54 (− 0.90, 1.98)	**KWTB + CC**	NR	NR
1.16 (0.77, 1.55)*	0.56 (0.15, 0.96)*	0.53 (0.03, 1.03)*	− 0.01 (− 1.36, 1.34)	**HP**	0.76 (0.43, 1.09)
1.88 (1.12, 2.63)*	1.27 (0.51, 2.03)*	1.25 (0.43, 2.07)*	0.71 (− 0.79, 2.20)	0.72 (0.07, 1.36)*	**KWTB**
**UCLA** MD (95% CI)					
**LCP + CC**	NR	NR	NR		
0.53 (− 1.38, 2.44)	**CC**	NR	1.24 (0.78, 1.69)		
0.63 (− 0.81, 2.07)	0.10 (− 1.16, 1.35)	**LCP**	1.05 (0.16, 1.94)^a^		
1.65 (0.01, 3.29)*	1.12 (0.15, 2.09)*	1.02 (0.23, 1.81)*	**HP**		
**CCD** MD (95% CI)					
**HP**	NR	− 0.78 (− 1.82, 0.25)^b^			
− 0.76 (− 1.76, 0.23)	**LCP + CC**	− 0.30 (− 1.25, 0.64)^b^			
− 1.06 (− 2.48, 0.36)	− 0.29 (− 1.30, 0.72)	**LCP**			

Fixation methods are ordered by the SUCRA value, and the top left is the best, whereas the bottom right is the worst. Estimates on the upper right are direct comparisons (i.e., head- to-head studies); the lower-left estimates are from the network meta-analysis

*MD (95% CI)* mean difference and 95%confidence interval, *OR (95% CI)* odd ratio and 95%confidence interval, *HP* hook plate, *LCP* locking compression plate, *CC* coracoclavicular reconstruction, *LCP* *+* *CC*, combination of locking compression plate and coracoclavicular reconstruction, *KWTB* Kirshner wire and tension band, *KWTB* + *CC* combination of Kirshner wire and tension band and coracoclavicular reconstruction, *KW* Kirshner wire, *NR* not reported, *NFT* no further treatment

^a^Significant heterogeneity was tested between the direct comparisons, and sensitivity analysis confirmed the robustness of the results

^b^Significant heterogeneity was tested between the direct comparisons

*p value < 0.05 statistically significant different

### University of California at Los Angeles score (UCLAs)

The NMA of the UCLAs comprised 6 studies [20, 34, 39, 44, 53, 55, 56] with 6 direct comparisons of 4 different internal fixation methods (HP, LCP, CC, and LCP + CC) (Additional file 5: Fig. S2A). The network map is presented in Additional file 6: Fig. S3A. The results of the consistency NMA are presented in Table 2 and indicated that LCP + CC, CC, and LCP were much more effective for UDCFs than HP fixation in terms of the UCLAs. LCP + CC fixation had a significantly higher UCLAs than HP fixation (1.65 (95% CI 0.01–3.29)), but no measurable difference was present when compared with CC and LCP fixation. In the ranking of the best treatment, LCP + CC fixation was first, with a SUCRA of 82.1 (Additional file 7: Fig. S4A) and a probability of being the best treatment of 64.4%. There was no measurable inconsistency (*p* = 0.96) within the network. In addition, the prediction intervals were assessed and presented with a graph (Additional file 3: Fig. S5B). The funnel plot and Egger's test did not indicate any risk of publication bias (Additional file 8: Fig. S6A, *P* = 0.563).

### Radiographic outcomes

The NMA of the coracoclavicular distance (CCD) comprised 6 studies [37, 39, 42, 61, 64, 68] with 6 direct comparisons of 3 different internal fixation methods (Additional file 5: Fig. S2B). The network map is presented in Additional file 6: Fig. S3B. The results of the consistency NMA are presented in Table 2 and showed no significant difference in CCD among the 3 internal fixation methods. In the ranking of the best treatment, HP was first, with a SUCRA of 93.1 (Additional file 7: Fig. S4B) and a probability of being the best treatment of 89.1% for CCD. There was no measurable inconsistency (*p* > 0.05) within the network. The predictive intervals were estimated and plotted (Additional file 3: Fig. S5C). The funnel plot and Egger's test did not indicate any risk of publication bias (Additional file 8: Fig. S6B, *P* = 0.629).

### Complications

Total complications and implant-related complications were reported in all 41 studies [14–16, 20, 21, 33–68], and 45 direct comparisons were synthesized in an NMA of 7 internal fixation methods (HP, LCP, CC, LCP + CC, KWTB, KWTB + CC, and KW) (Fig. 2B and Additional file 5: Fig. S2C). The network map is shown in Fig. 3B and Additional file 6: Fig. S3C. The results of the consistency NMA are presented in Table 3, which showed that LCP + CC, LCP, CC, and KWTB + CC were much safer fixation methods in terms of total complications and implant-related complications than CC, followed by LCP and HP fixation. Compared with HP, KWTB, and KW fixation, LCP + CC fixation had statistically significantly fewer total complications (0.22 (95% CI 0.09–0.51), 0.05 (95% CI 0.01–0.20), and 0.01 (95% CI 0.00–0.11), respectively) and implant-related complications (0.31 (95% CI 0.14–0.70), 0.12 (95% CI 0.03–0.43), and 0.01 (95% CI 0.00–0.20), respectively). There was no measurable difference between LCP + CC and LCP, CC, or KWTB + CC. In the ranking of the best treatment, LCP + CC fixation was first, with an SUCRA of 93.9 (Fig. 4B) and a probability of being the best treatment of 68.8% for total complications, and a SUCRA of 84.5 (Additional file 7: Fig. S4C) and a probability of being the best treatment of 34.5% for implant-related complications. There was no measurable inconsistency (*p* = 0.770) within the network. In addition, the prediction intervals were assessed and presented with a graph (Additional file 3: Fig. S5D, E). The funnel plot and Egger's test did not indicate any risk of publication bias (Fig. 5B, *P* = 0.638; Additional file 8: Fig. S6C, *P* = 0.341). The confidence of the total complications for most of the comparisons of interest was graded as low (Additional file 9: Table S2).

**Table 3:** League table of different internal fixation comparisons of complications for UDCF

**Total complications** OR (95% CI)						
**LCP + CC**	0.55 (0.23, 1.29)	NR	NR	0.19 (0.05, 0.66)	NR	
0.65 (0.27, 1.53)	**LCP**	NR	NR	0.25 (0.12, 0.52)^b^	NR	
0.38 (0.12, 1.20)	0.59 (0.21, 1.69)	**CC**	NR	0.53 (0.22, 1.28)^b^	NR	
0.27 (0.02, 2.98)	0.42 (0.03, 5.37)	0.72 (0.05, 10.20)	**KWTB + CC**	NR	NR	
0.22 (0.09, 0.51)*	0.34 (0.16, 0.71)*	0.57 (0.26, 1.25)	0.80 (0.06, 10.09)	**HP**	0.22 (0.06, 0.84)^a^	
0.05 (0.01, 0.20)*	0.08 (0.02, 0.29)*	0.14 (0.04, 0.50)*	0.20 (0.01, 3.03)	0.25 (0.09, 0.67)*	**KWTB**	
0.01 (0.00, 0.11)*	0.01 (0.00, 0.17)*	0.01 (0.00, 0.29)*	0.02 (0.00, 0.93)*	0.02 (0.00, 0.46)*	0.10 (0.01, 1.55)	**KW**
**Implant related complications** OR (95% CI)						
**LCP + CC**	2.92 (0.89, 9.59)^b^	NR	NR	0.40 (0.22, 0.75)	NR	
0.78 (0.34, 1.80)	**LCP**	NR	NR	0.18 (0.06, 0.50)	NR	
0.62 (0.18, 2.14)	0.79 (0.24, 2.59)	**CC**	NR	0.47 (0.13, 1.75)	NR	
0.72 (0.01, 45.37)	0.93 (0.01, 63.35)	1.17 (0.02, 88.04)	**KWTB + CC**	NR	NR	
0.31 (0.14, 0.70)*	0.40 (0.19, 0.85)*	0.50 (0.20, 1.28)	0.43 (0.01, 29.00)	**HP**	NR	
0.12 (0.03, 0.43)*	0.16 (0.05, 0.52)*	0.20 (0.05, 0.73)*	0.17 (0.00, 12.76)	0.40 (0.16, 0.98)*	**KWTB**	
0.01 (0.00, 0.20)*	0.01 (0.00, 0.24)*	0.02 (0.00, 0.32)*	0.02 (0.00, 2.40)	0.04 (0.00, 0.55)*	0.10 (0.01, 1.19)	**KW**
**Re-operation** OR (95% CI)						
**LCP + CC**	NR	0.35 (0.08, 1.57)	0.03 (0.00, 0.33)	NR	NR	
0.68 (0.19, 2.37)	**CC**	NR	0.26 (0.10, 0.67)	NR	NR	
0.46 (0.18, 1.18)	0.68 (0.23, 1.98)	**LCP**	0.82 (0.21, 3.26)	NR	NR	
0.34 (0.12, 0.92)*	0.50 (0.22, 1.13)	0.73 (0.33, 1.63)	**HP**	0.36 (0.09, 1.45)	NR	
0.14 (0.03, 0.73)*	0.21 (0.04, 0.96)*	0.30 (0.06, 1.41)	0.41 (0.11, 1.53)	**KWTB**	NR	
0.03 (0.00, 0.73)	0.05 (0.00, 1.39)	0.07 (0.00, 1.83)	0.10 (0.00, 2.55)	0.25 (0.01, 8.00)	2.60 (0.04, 166.59)	**NFT**
0.01 (0.00, 0.22)*	0.02 (0.00, 0.31)*	0.03 (0.00, 0.45)*	0.04 (0.00, 0.55)*	0.10 (0.01, 0.94)*	**KW**	
**Nonunion and delayed union** OR (95% CI)						
**LCP + CC**	NR	NR	0.42 (0.05, 3.70)	NR	NR	
0.74 (0.26, 2.13)	**LCP**	NR	0.71 (0.24, 2.16)	NR	NR	
0.73 (0.19, 2.75)	0.99 (0.31, 3.12)	**CC**	0.82 (0.27, 2.55)	NR	NR	
0.67 (0.24, 1.85)	0.90 (0.40, 2.05)	0.91 (0.38, 2.19)	**HP**	0.53 (0.10, 2.92)	NR	
0.49 (0.04, 6.28)	0.66 (0.06, 7.88)	0.67 (0.06, 8.14)	0.74 (0.07, 7.61)	**KWTB**	NR	
0.07 (0.00, 1.63)	0.10 (0.00, 2.62)	0.10 (0.00, 2.93)	0.11 (0.00, 2.88)	0.15 (0.00, 8.25)	0.19 (0.01, 6.31)	**NFT**
0.39 (0.07, 2.04)	0.53 (0.11, 2.46)	0.53 (0.11, 2.57)	0.59 (0.16, 2.15)	0.79 (0.07, 9.22)	**KW**	

^a^Significant heterogeneity was tested between the direct comparisons, and sensitivity analysis confirmed the robustness of the results

^b^Significant heterogeneity was tested between the direct comparisons

*p value < 0.05 statistically significant different

The forest plots of reoperation and nonunion and delayed union are presented in Additional file 5: Fig. S2D, E, and the network maps are presented in Additional file 6: Fig. S3D and E. The results of the consistency NMA are shown in Table 3 and showed no significant difference in nonunion or delayed union among the 6 different internal fixation methods. LCP + CC had a lower reoperation rate than HP, followed by KWTB and KW, and was first, with a SUCRA of 93.9 (Additional file 7: Fig. S4D) and a probability of being the best treatment of 70.4% for reducing the rate of reoperation. In the ranking of the best treatment, LCP + CC was also ranked first, with a SUCRA of 78.2 (Additional file 7: Fig. S4E) and a probability of being the best treatment of 39.5% for reducing the rate of nonunion and delayed union. There was no measurable inconsistency (*p* = 0.7713) within the network. The predictive interval plots are presented in Additional file 3: Fig. S5F and G. The funnel plot and Egger's test did not show any risk of publication bias (Additional file 8: Fig. S6D, *P* = 0.306; Additional file 8: Fig. S6G, *P* = 0.662).

### Surgical outcomes

The forest plots of the NMA for surgical outcomes are shown in Additional file 5: Fig. S2F–I, and the network maps are presented in Additional file 6: Fig. S3F–I. The results (Table 4) showed that CC (SUCRA: 100, Additional file 7: Fig. S4F) fixation was associated with a smaller incision than LCP, LCP + CC, and HP and also had less blood loss than HP (Additional file 7: Fig. S4H). LCP (SUCRA: 83.6, Additional file 7: Fig. S4G) fixation had a shorter operative time than CC, but no statistically significant difference was present compared with HP and LCP + CC. LCP + CC (SUCRA: 81.1) had a shorter union time than HP and CC (Additional file 7: Fig. S4I). There was significant measurable inconsistency (*p* = 0.0084) within the NMA for incision size. The node-splitting analysis revealed inconsistency between HP versus CC (*p* = 0.019) and LCP versus CC (*p* = 0.001). The sensitivity analysis confirmed the robustness of the results. There was no measurable inconsistency within the network for the operative time, blood loss, or union time. In addition, the prediction intervals were assessed and presented with a graph (Additional file 3: Fig. S5H–K). The funnel plot and Egger's test did not indicate any risk of publication bias (Additional file 8: Fig. S6F, *P* = 0.086; 6G, *P* = 0.346; 6H, *P* = 0.057; 6I, *P* = 0.105).

**Table 4:** League table of different internal fixation comparisons of surgical outcomes for UDCF

**Incision** MD (95*% CI)					
**CC**	NR	NR	− 3.66 (− 4.54, − 2.77)^a^		
− 3.13 (− 4.60, − 1.66)*	**LCP**	− 0.32 (− 0.80, 0.17)	− 0.76 (− 2.6, 1.08)^b^		
− 3.45 (− 5.39, − 1.51)*	− 0.32 (− 1.58, 0.94)	**LCP + CC**	NR		
− 3.70 (− 4.66, − 2.75)*	− 0.57 (− 1.83, 0.69)	− 0.25 (− 2.04, 1.53)	**HP**		
**Operative time** MD (95% CI)					
**LCP**	− 0.01 (− 0.28, 0.27)	− 0.48 (− 0.82, − 0.14)	NR		
0.50 (− 0.50, 1.51)	**HP**	− 0.26 (− 0.58, 0.06)	− 0.27 (− 0.51, − 0.02)		
− 0.30 (− 0.73, 0.13)	0.43 (− 0.18, 1.05)	**LCP + CC**	NR		
− 0.65 (− 1.28, − 0.03)*	0.04 (− 0.24, 0.33)	− 0.35 (− 0.99, 0.28)	**CC**		
**Blood loss** MD (95% CI)					
**CC**	NR	− 1.8 (− 3.70, 0.12)^b^	NR		
− 1.33 (− 3.12, 0.47)	**LCP**	− 0.07 (− 0.38, 0.24)	− 0.39 (− 0.88, 0.10)		
− 1.60 (− 2.96, − 0.24)*	− 0.27 (− 1.62, 1.07)	**HP**	− 0.36 (− 0.80, 0.08)		
− 1.85 (− 3.96, 0.26)	− 0.52 (− 2.20, 1.15)	− 0.25 (− 1.92, 1.42)	**LCP + CC**		
**Union time** MD (95% CI)					
**LCP + CC**	NR	− 0.40 (− 0.80, 0.00)	NR	− 0.61 (− 1.16, − 0.05)*	NR
− 0.08 (− 1.42, 1.26)	**KWTB**	NR	NR	NR	NR
− 0.34 (− 0.85, 0.18)	− 0.25 (− 1.60, 1.10)	**LCP**	NR	− 0.37 (− 1.03, 0.30)^b^	NR
− 0.30 (− 2.11, 1.52)	− 0.21 (− 1.44, 1.01)	0.04 (− 1.78, 1.86)	**KW**	NR	NR
− 0.64 (− 1.08, − 0.19)*	− 0.55 (− 1.82, 0.71)	− 0.30 (− 0.78, 0.18)	− 0.34 (− 2.10, 1.42)	**HP**	− 0.18 (− 0.82, 0.47)^b^
− 0.81 (− 1.60, − 0.02)*	− 0.73 (− 2.15, 0.69)	− 0.47 (− 1.28, 0.33)	− 0.51 (− 2.39, 1.36)	− 0.17 (− 0.82, 0.47)	**CC**

^a^Significant heterogeneity was tested between the direct comparisons, and sensitivity analysis confirmed the robustness of the results

^b^Significant heterogeneity was tested between the direct comparisons

*p value < 0.05 statistically significant different

**Fig. 6:**
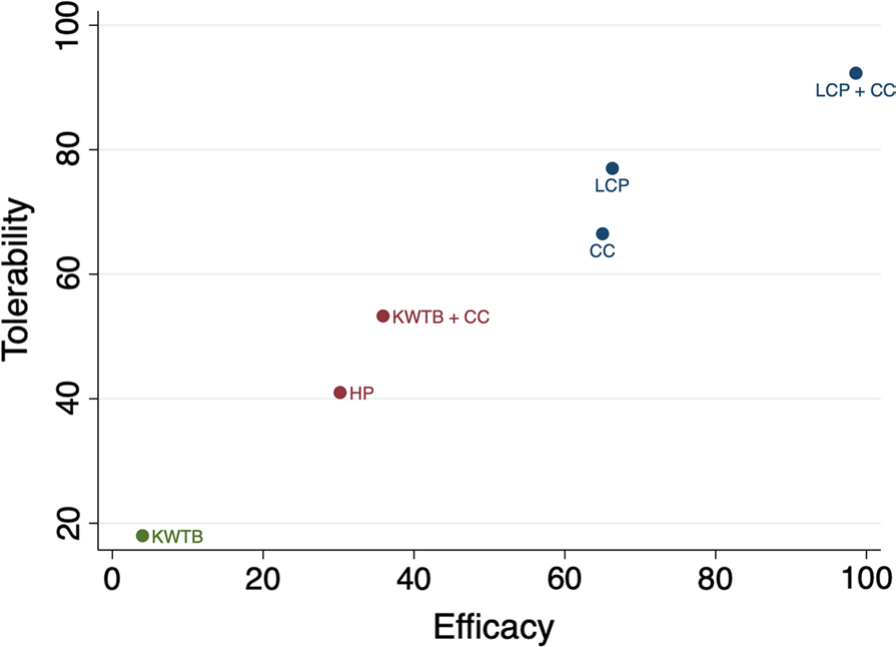
Cluster-rank plot of CMS and total complications for UDCFs

To examine the relative effectiveness and safety of different internal fixation methods, cluster ranking was conducted and indicated that LCP + CC appears to display the greatest potential to be the optimum treatment (Fig. 6).

## Discussion

To the best of our knowledge, there has been no systematic review or meta-analysis comparing all internal fixation methods (including single internal fixation and combined internal fixation) prior to this review. This systematic review and NMA showed that LCP + CC fixation was associated with better efficacy and fewer complications than any other internal fixation method for UDCFs. On the other hand, HP, KWTB and KW were associated with lower functional scores and a higher risk of complications. The results indicated that LCP + CC had the greatest potential to be the optimum fixation method for patients with UDCFs. Our NMA provides a reference for surgeons when choosing the best internal fixation method for UDCFs.

The CC ligament is one of the important stabilizing structures of the distal clavicle. CC rupture is the main factor in fracture displacement in proximal fractures; therefore, CC reconstruction is very important for fracture reduction and maintenance reduction [69–71]. Yagnik et al. [22] reported that arthroscopy-assisted CC reconstruction of UDCFs reduced implant-related complications and the risk of reoperation, with the same good functional outcomes and union rates as LCP and HP fixation. For UDCFs, especially fractures with a comminuted distal fragments, CC fixation that only fixes the proximal end of the fracture is obviously not enough to achieve the standard of fracture healing.

The advantage of LCP is that its lateral section increases the number of locking screws and the angle of fixation of the screws, increasing the grip and resistance to extraction of the distal fracture block and increasing the effectiveness of fixation of the fracture [72]. In contrast to HP, LCP does not invade the subacromial space and the acromioclavicular joint, reducing complications such as osteoarthritis of the acromioclavicular joint, rotator cuff injury, subacromial impingement and osteolysis [51, 56]. The plate requires a smaller incision and does not require a second operation to remove the internal fixation device. KWTB and KW have a higher risk of implant-related complications, such as pin displacement and skin irritation, which increases the risk of infection and increases fracture loss. Compared with HP, LCP showed better recovery of shoulder function and fewer complications related to pain and limited abduction when treating Neer type II DCFs [73]. However, LCP is significantly less effective for fixing unstable fractures than stable fractures. Our results showed that LCP + CC fixation was associated with better efficacy and fewer complications, as well as a shorter incision and less blood loss but a much longer operative time than other internal fixation methods for UDCFs. Biomechanical studies have shown that LCP + CC can achieve greater fracture stability than the fixation method alone [74, 75]. The mechanism may be that CC fixation protects against upward stresses on the proximal clavicle and achieves fracture repositioning and stabilization, thereby reducing the incidence of screw extraction with LCP and internal fixation failure in the distal clavicle. Therefore, the LCP + CC group seemed to have better outcomes (functional and complications) after fracture fixation than the other groups.

The results of the NMA agreed with the results of the direct meta-analysis that LCP + CC appeared to be the best option in terms of postoperative functional scores and complications in UDCFs. Although prediction interval plots from the NMA indicated that the use of LCP + CC may be ineffective in the future compared to other internal fixation methods, this study offers trends in outcomes between the different internal fixation methods.

### Limitations

This research has some limitations. There may be too many internal fixation methods for UDCFs and none of them can achieve the good results, only one low-quality RCT (Additional file 2: Fig. S1B) was retrieved and included in this NMA. The results of the NMA were not consistent with the only one RCT compared HP and KWTB, as well as the other comparative studies. The fracture classifications included in the study were not unified, and subgroup analyses could not be performed due to insufficient data. There were related biases, which may have impacted the results of the study. There is a certain degree of bias that affected the results of the study. The follow-up times of the included studies were different, which led to heterogeneity among the studies, and the effect on the research results needs to be discussed. The quality of the studies included in the meta-analysis was not very high, and the reliability of the comprehensive CINeMA evaluation was medium to low. Therefore, a multi-center RCT with appropriate random sequence generation, allocation concealment and blinding were required for the treatment of UDCFs. The fracture types of all included cases must be clarified to conduct a subgroup analysis for different types of fractures; all surgeons need to be trained in the surgical procedure to reduce the bias; blinding must be assessed in measurers and data analysts.

## Conclusions

Overall, the results of this study indicate that LCP + CC appears to be the best internal fixation method for UDCF. Limited to the quality and quantity of the included studies, much larger and higher-quality RCTs are required to confirm these conclusions.

## Supplementary Information

**Additional file 1:** **Appendix 1.** Search strategies for electronic databases.

**Additional file 2: Figure S1.** Risk of bias assessment for the included studies. **A** Risk of bias summary for the nonrandomized studies. **B** Risk of bias summary for the randomized studies. **C** The average risk of bias contribution for each comparison within the network.

**Additional file 3: Fig. S5.** Predictive interval plots of the postoperative function assessment, radiographic outcome, complications and surgical outcomes between the comparisons. **A** CMS; **B** UCLAs; **C**CCD; **D** Total complications; **E** Implant-related complications; **F** Reoperation; **G** Nonunion and delayed union; **H** Incision; **I** Operative time; **J** Blood loss; **K**Union time.

**Additional file 4: Table S1.** The confidence rating for the CMS of different internal fixation comparisons for UDCFs.

**Additional file 5: Fig. S2.** Forest plots of the meta-analysis of different internal fixation methods for UDCFs. **A**. UCLAs; **B** CCD; **C**Implant-related complications; **D** Reoperation; **E** Nonunion and delayed union; **F** Incision; **G** Operative time; **H** Blood loss; **I** Union time. 1, HP; 2, LCP; 3, CC; 4, LCP + CC; 5, KWTB; 6, KWTB + CC; 7, KW.

**Additional file 6: Fig. S3.** Network meta-analysis maps of the outcomes. **A** UCLAs; **B** CCD; **C** Implant-related complications; **D** Reoperation; **E** Nonunion and delayed union; **F** Incision; **G** Operative time; **H** Blood loss; **I** Union time. Each node represents an intervention, and the size of the node is proportional to the number of patients assigned to the intervention. The lines indicate direct comparisons between nodes, and the size of the line is proportional to the number of trials comparing each pair of nodes.

**Additional file 7: Fig. S4.** Rankogram of different internal fixation methods for outcomes. **A**. UCLAs; **B** CCD; **C** Implant-related complications; **D** Reoperation; **E** Nonunion and delayed union; **F** Incision; **G** Operative time; **H** Blood loss; **I** Union time.

**Additional file 8: Fig. S6.** Network meta-analysis funnel plots for the assessment of publication bias of the included studies. **A** UCLAs; **B** CCD; **C** Implant-related complications; **D** Reoperation; **E** Nonunion and delayed union; **F**Incision; **G** Operative time; **H** Blood loss; **I** Union time. **A**, **C**–**H** A: HP, B: LCP, C: CC, D: LCP + CC, E: KWTB, F: KWTB + CC, G: KW; **B**: A: HP, B: LCP, C: LCP + CC; **I** A: HP, B: LCP, C: CC, D: LCP + CC, E: KWTB, F: KW.

**Additional file 9: Table S2.** The confidence rating for total complications of different internal fixation comparisons for UDCFs.

## Data Availability

This study does not contain any third-party materials.

## Abbreviations

DCF: Distal clavicle fracture
UDCF: Unstable distal clavicle fracture
NMA: Network meta-analysis
HP: Hook plate
LCP: Locking compression plate
CC: Coracoclavicular reconstruction
LCP + CC: Combination of LCP and CC
KWTB: Kirshner wire and tension band
KWTB + CC: Combination of KWTB and CC
KW: Kirshner wire
CMS: Constant Murley Score
UCLAs: University of California at Los Angeles score
CCD: Coracoclavicular distance
RCTs: Randomized controlled trials
CINeMA: The confidence in network meta-analysis
ORs: Odds ratios
95% CIs: 95% Confidence intervals
MDs: Mean deviations
SUCRA: Surface under cumulative ranking curve
