# Supplementary material for: Different internal fixation methods for unstable distal clavicle fractures in adults: a systematic review and network meta-analysis
Source: J Orthop Surg Res. 2022 Jan 24;17:43. doi: 10.1186/s13018-021-02904-6 (PMC8785604; doi:10.1186/s13018-021-02904-6)
Supplement: Supplementary file 1 — Additional file 1: Appendix 1. Search strategies for electronic databases. [file 13018_2021_2904_MOESM1_ESM.docx]

**Additional file 1: Appendix 1. Search strategies for electronic databases.**

1. **PubMed. (-20210916) 434.**

#1 "Fracture Fixation, Internal"[Mesh]

#2 (Fixation, Internal Fracture) OR (Fixations, Internal Fracture) OR (Fracture Fixations, Internal) OR (Internal Fracture Fixation) OR (Internal Fracture Fixations) OR (Osteosynthesis, Fracture) OR (Fracture Osteosyntheses) OR (Fracture Osteosynthesis) OR (Osteosyntheses, Fracture)

#3 #1 OR #2

#4 (distal) OR (lateral)

#5 #3 AND #4

#6 "Clavicle"[Mesh]

#7 (Clavicles) OR (Collar Bone) OR (Bone, Collar) OR (Bones, Collar) OR (Collar Bones)

#8 #6 OR #7

#9 #5 AND #8

1. **Web of Science Core Collection (1985-present) (20210916). 151**

**#1 (((((ALL=(Clavicle)) OR ALL=(Clavicles)) OR ALL=(Collar Bone)) OR ALL=(Bone, Collar)) OR ALL=(Bones, Collar)) OR ALL=(Collar Bones) 4,864**

**#2 (ALL=(Distal)) OR ALL=(Lateral) 495,767**

**#3 (#1) AND #2 1,260**

**#4 (((((((((ALL=(Fracture Fixation, Internal)) OR ALL=(Fixation, Internal Fracture)) OR ALL=(Fixations, Internal Fracture)) OR ALL=(Fracture Fixations, Internal)) OR ALL=(Internal Fracture Fixation)) OR ALL=(Internal Fracture Fixations)) OR ALL=(Osteosynthesis, Fracture)) OR ALL=(Fracture Osteosyntheses)) OR ALL=(Fracture Osteosynthesis)) OR ALL=(Osteosyntheses, Fracture) 18,155**

**#5 (#3) AND #4 151**

1. **CBM 126**

**#1 锁骨远端骨折 1253 2021-09-19 10:05:39.0**

**#2 (锁骨远端骨折) AND ("1"[期刊类型]) 126 2021-09-19 10:05:47.0**

1. **Cochrane Trails 11**

#1 Mesh descriptor: [Clavicle] explode all trees

#2 (Clavicles) OR (Collar Bone) OR (Bone, Collar) OR (Bones, Collar) OR (Collar Bones)

#3 #1 OR #2

#4 (distal) OR (lateral)

#5 #3 AND #4

# 6 Mesh descriptor: [Fracture Fixation, Internal] explode all trees

#7 (Fixation, Internal Fracture) OR (Fixations, Internal Fracture) OR (Fracture Fixations, Internal) OR (Internal Fracture Fixation))OR (Internal Fracture Fixations) OR (Osteosynthesis, Fracture) OR (Fracture Osteosyntheses) OR (Fracture Osteosynthesis) OR (Osteosyntheses, Fracture)

#8 #6 OR #7

#9 #5 AND #8. (reviews 9 + trails 11=20)

1. **EMBASE. 324**

**#1. 'clavicle fracture'/exp 3,909**

**#2. clavicula AND fracture OR (clavicular AND fracture) OR (fractura AND claviculae) OR (fracture, AND clavicle) 6,414**

**#3. #1 OR #2 6,414**

**#4. 'osteosynthesis'/exp 46,953**

**#5. (fracture AND fixation, AND internal) OR (gleitosteosynthesis) OR (internal AND fixation) OR (internal AND fracture AND fixation) OR (osteo AND synthesis) 33,351**

**#6. #4 OR #5 62,290**

**#7. distal OR lateral 775,092**

**#8. #3 AND #7 1,195**

**#9. #6 AND #8 324**
