# Supplementary material for: Different internal fixation methods for unstable distal clavicle fractures in adults: a systematic review and network meta-analysis
Source: J Orthop Surg Res. 2022 Jan 24;17:43. doi: 10.1186/s13018-021-02904-6 (PMC8785604; doi:10.1186/s13018-021-02904-6)
Supplement: Supplementary file 2 — Additional file 2: Figure S1. Risk of bias assessment for the included studies. A Risk of bias summary for the nonrandomized studies. B Risk of bias summary for the randomized studies. C The average risk of bias contribution for each comparison within the network. [file 13018_2021_2904_MOESM2_ESM.docx]

**Additional file 2: Figure S1.** Risk of bias assessment for the included studies. (A). Risk of bias summary for the nonrandomized studies. (B). Risk of bias summary for the randomized studies. (C). The average risk of bias contribution for each comparison within the network.

**Supplementary Figure 1A**

**Supplementary Figure 1B**

**Supplementary Figure 1C**
