# Supplementary material for: Different internal fixation methods for unstable distal clavicle fractures in adults: a systematic review and network meta-analysis
Source: J Orthop Surg Res. 2022 Jan 24;17:43. doi: 10.1186/s13018-021-02904-6 (PMC8785604; doi:10.1186/s13018-021-02904-6)
Supplement: Supplementary file 3 — Additional file 3: Fig. S5. Predictive interval plots of the postoperative function assessment, radiographic outcome, complications and surgical outcomes between the comparisons. A CMS; B UCLAs; CCCD; D Total complications; E Implant-related complications; F Reoperation; G Nonunion and delayed union; H Incision; I Operative time; J Blood loss; KUnion time. [file 13018_2021_2904_MOESM3_ESM.docx]

**Additional file 3: Figure S5.** Predictive interval plots of the postoperative function assessment, radiographic outcome, complications and surgical outcomes between the comparisons. (A). CMS; (B). UCLAs; (C). CCD; (D). Total complications; (E). Implant-related complications; (F). Reoperation; (G). Nonunion and delayed union; (H). Incision; (I). Operative time; (J). Blood loss; (K). Union time.

**Supplementary Figure 5A**

**
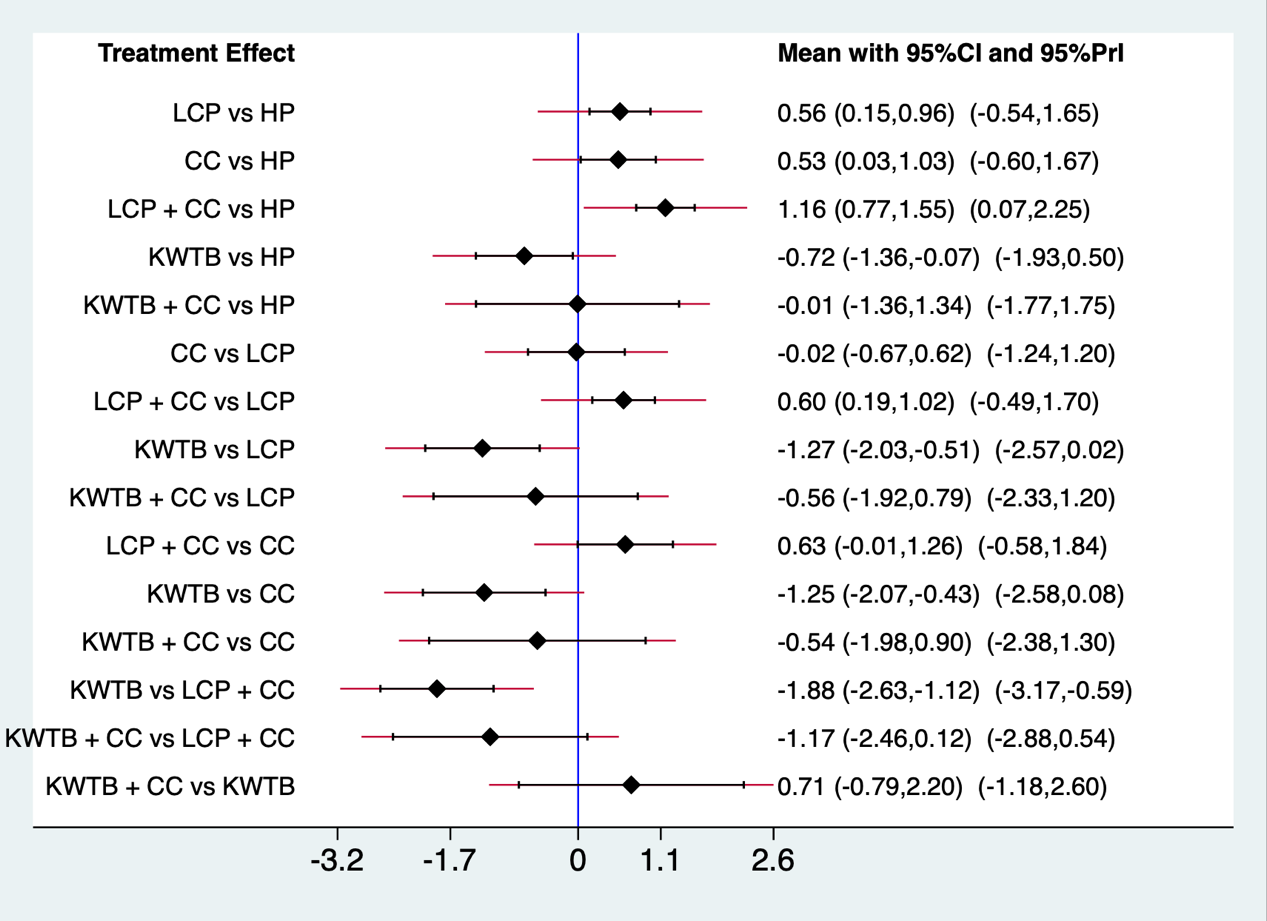
**

**Supplementary Figure 5B**

**
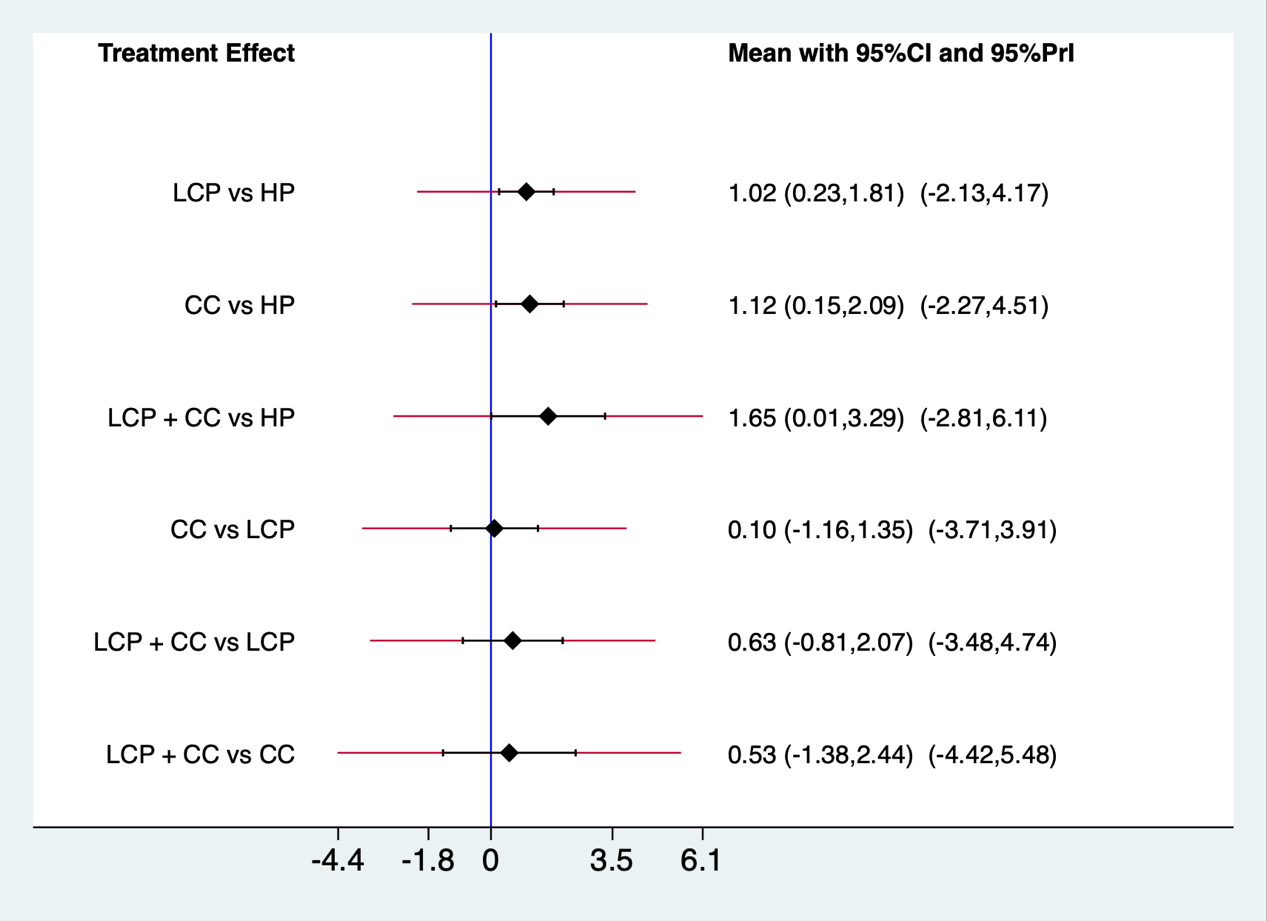
**

**Supplementary Figure 5C**

**
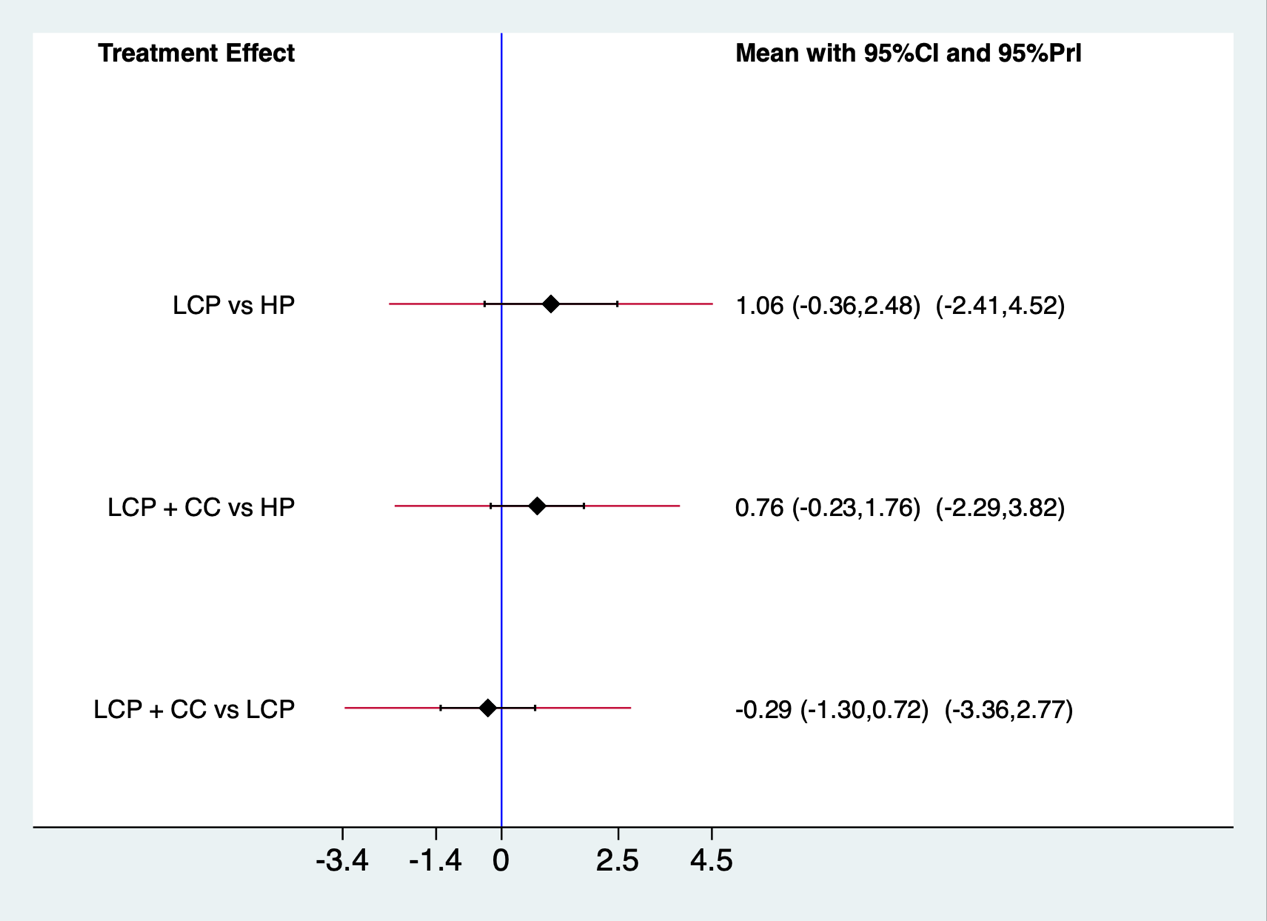
**

**Supplementary Figure 5D**

**
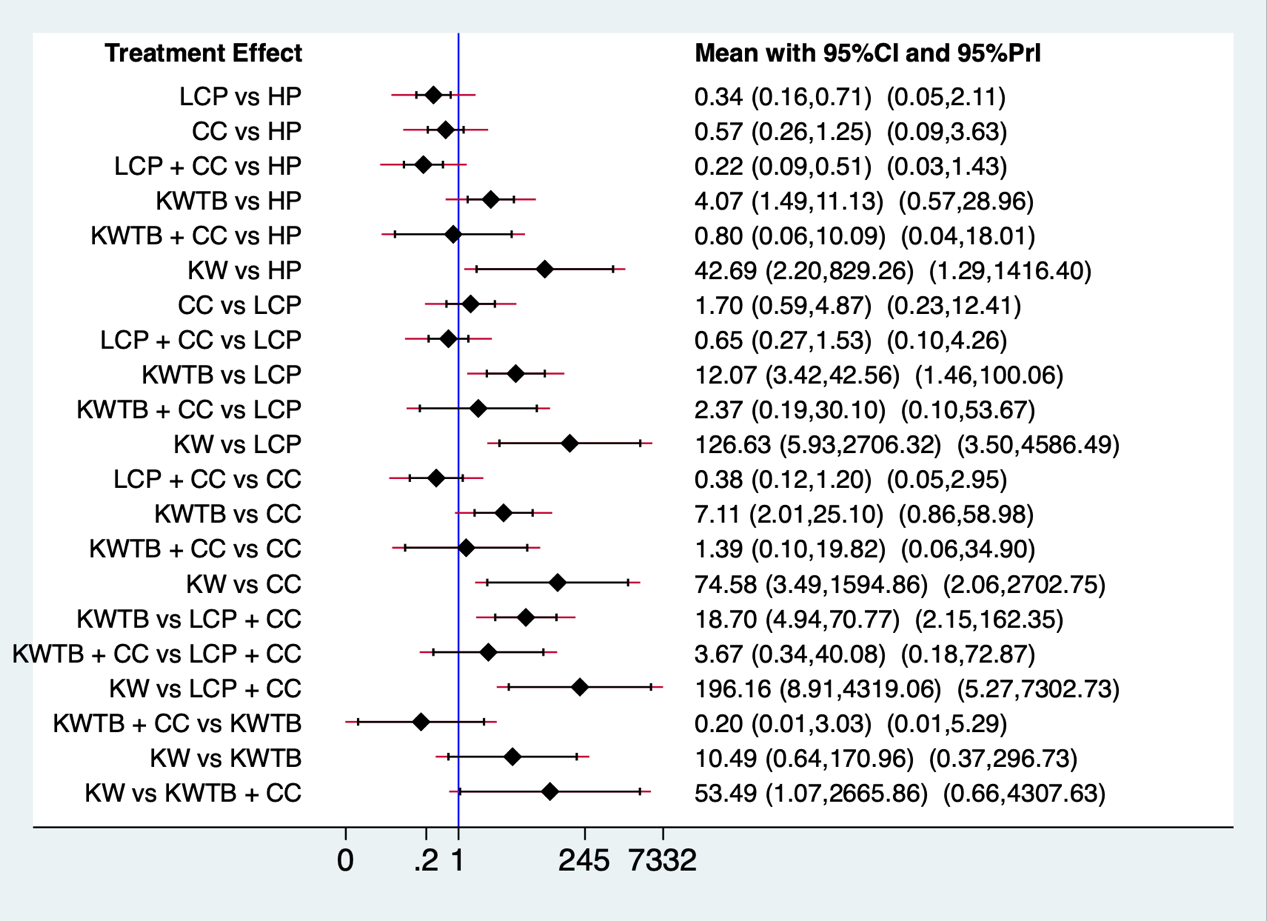
**

**Supplementary Figure 5E**

**
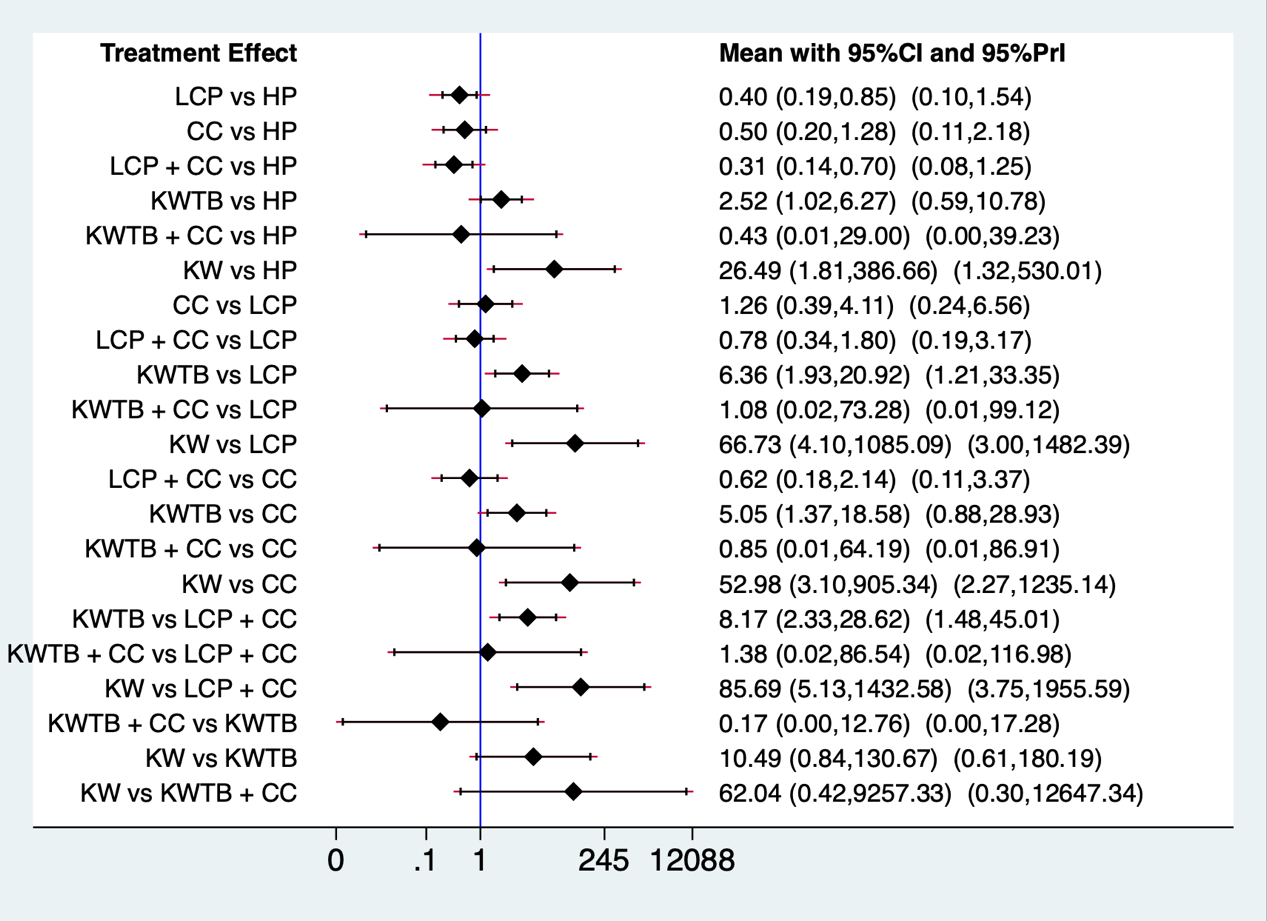
**

**Supplementary Figure 5F**

**
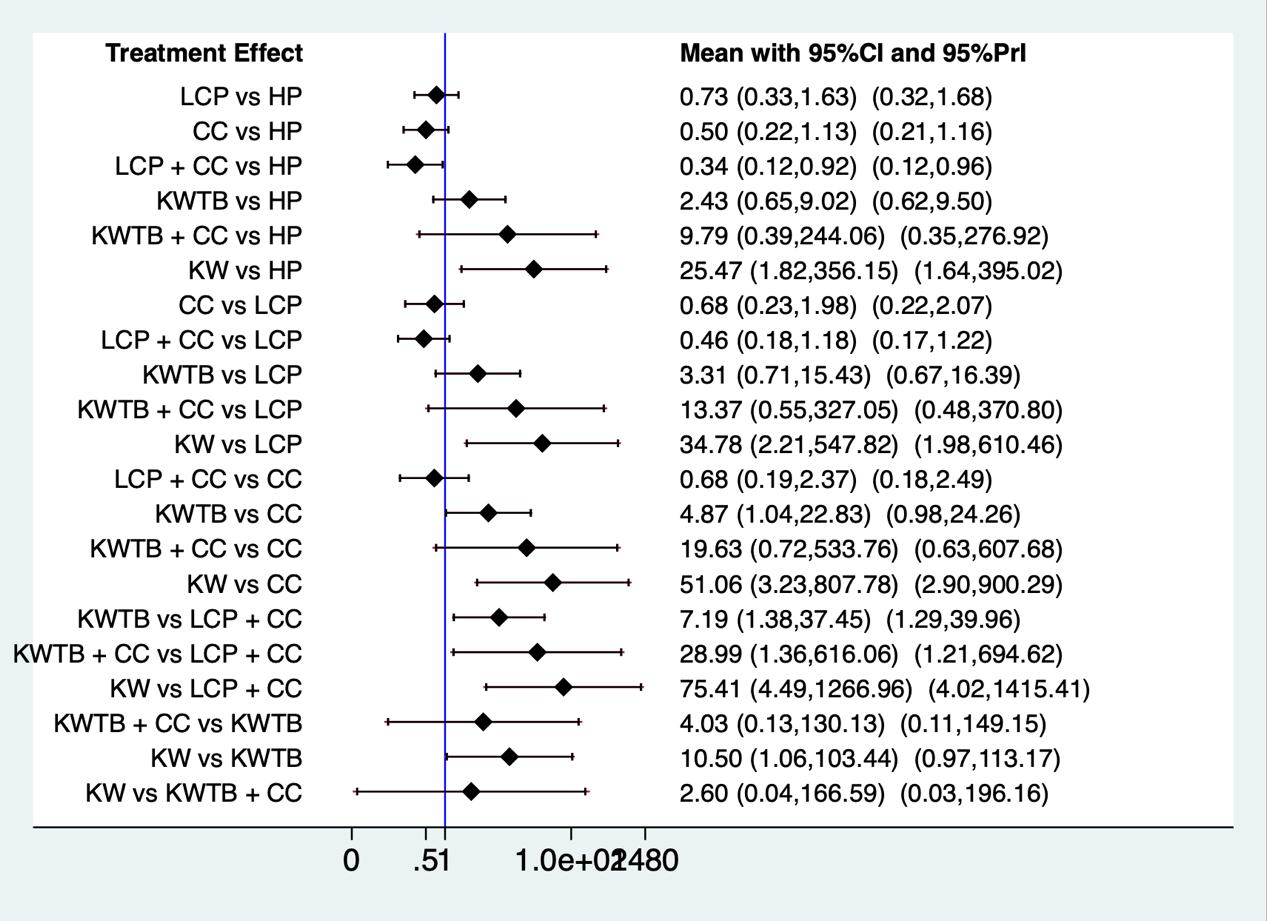
**

**Supplementary Figure 5G**

**
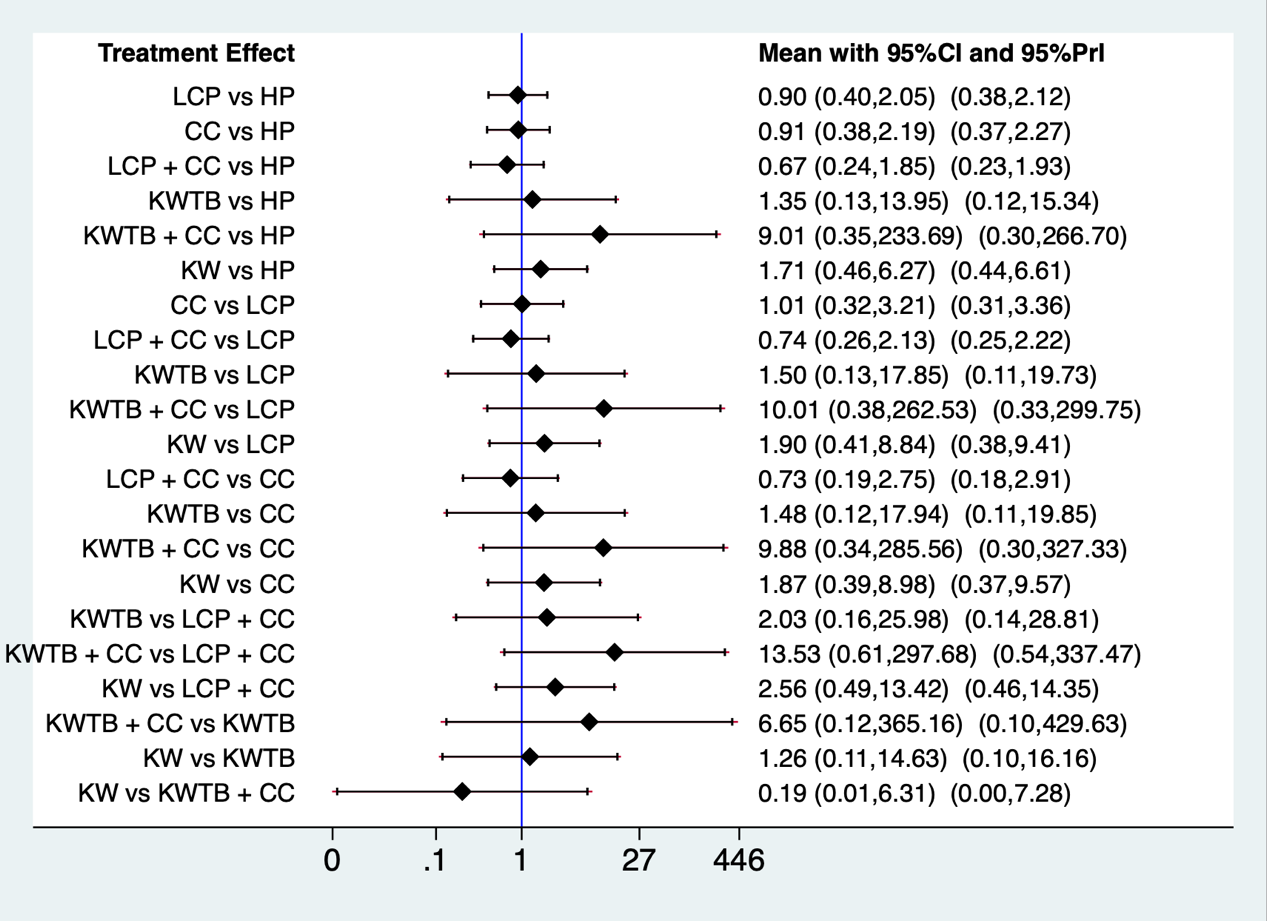
**

**Supplementary Figure 5H**

**
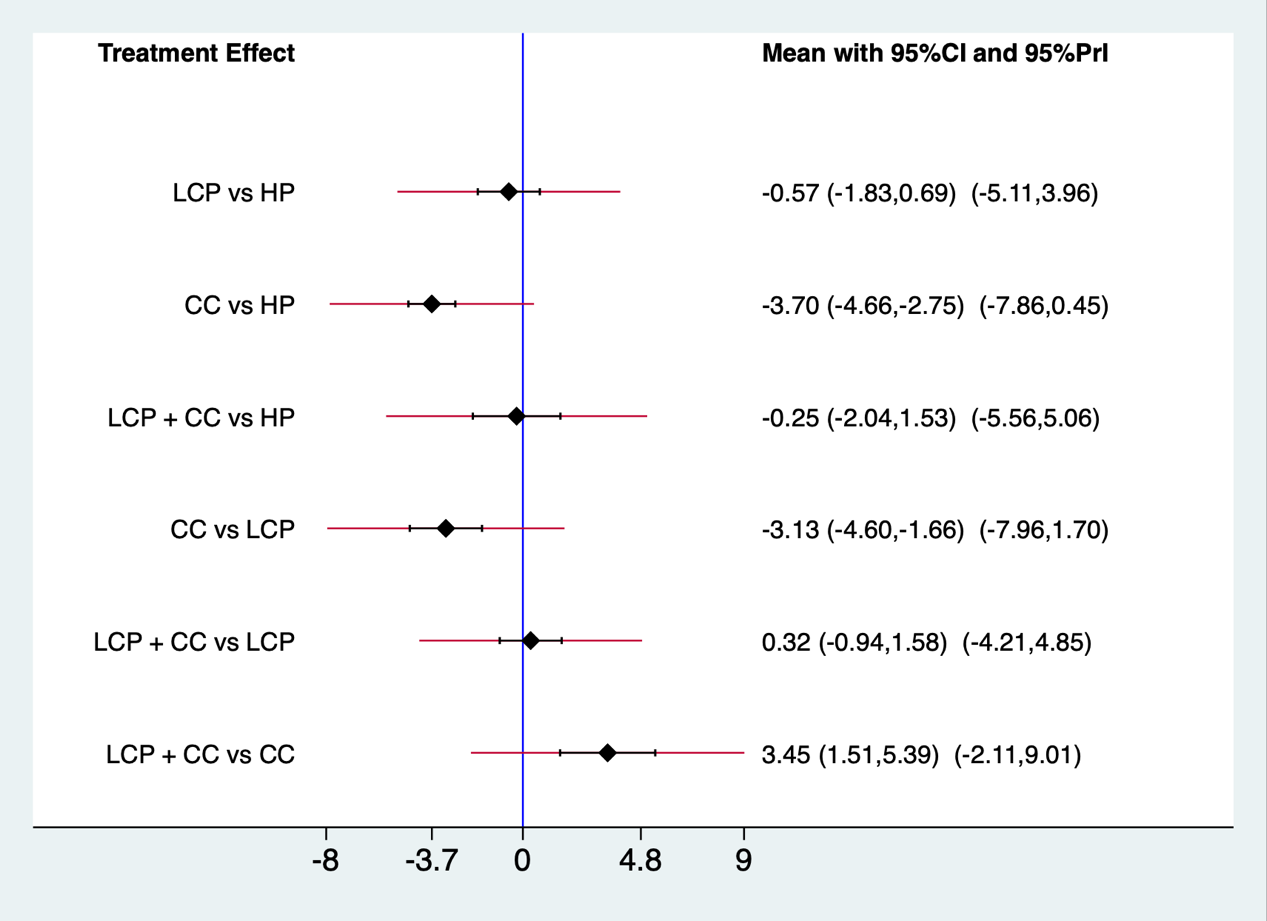
**

**Supplementary Figure 5I**


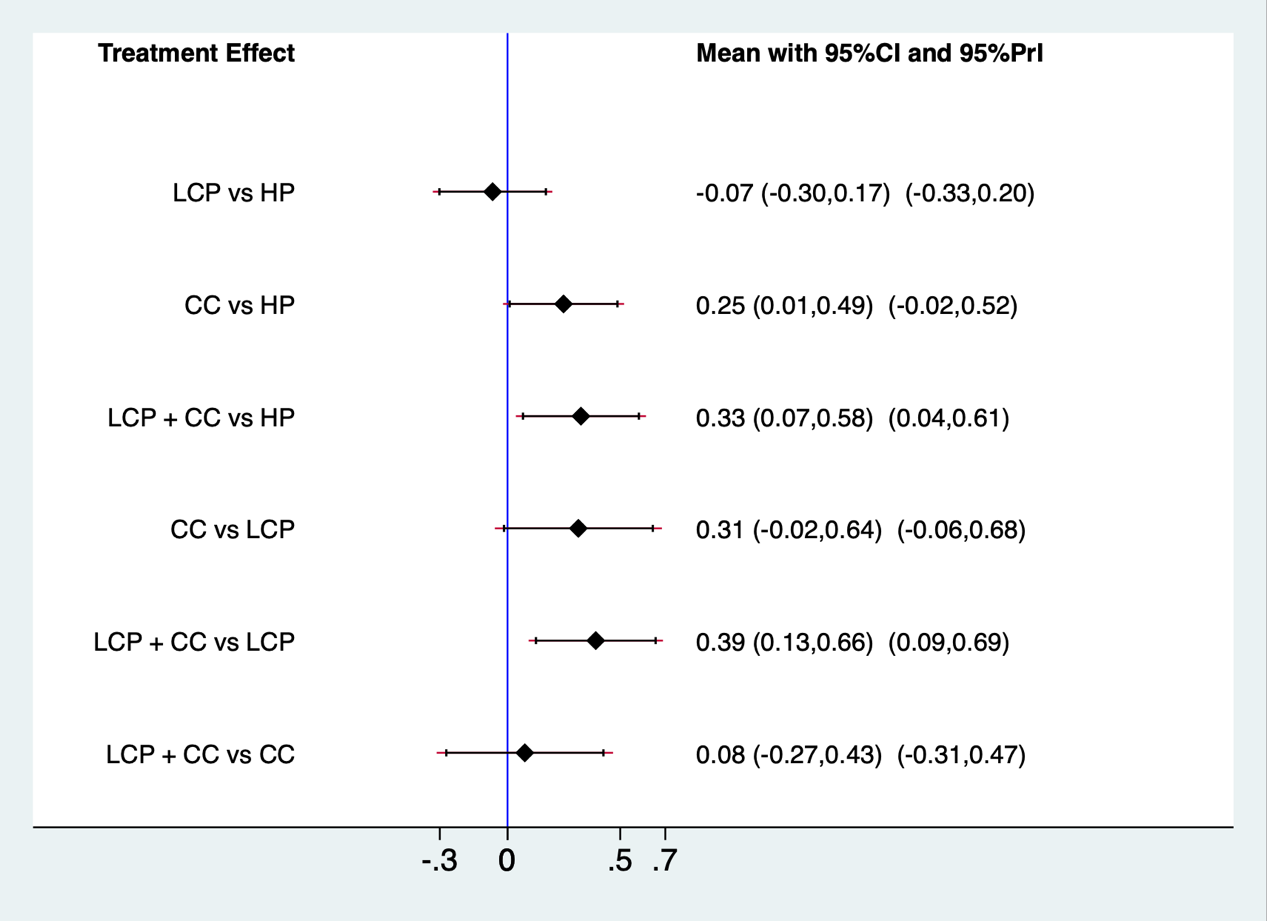


**Supplementary Figure 5J**

**
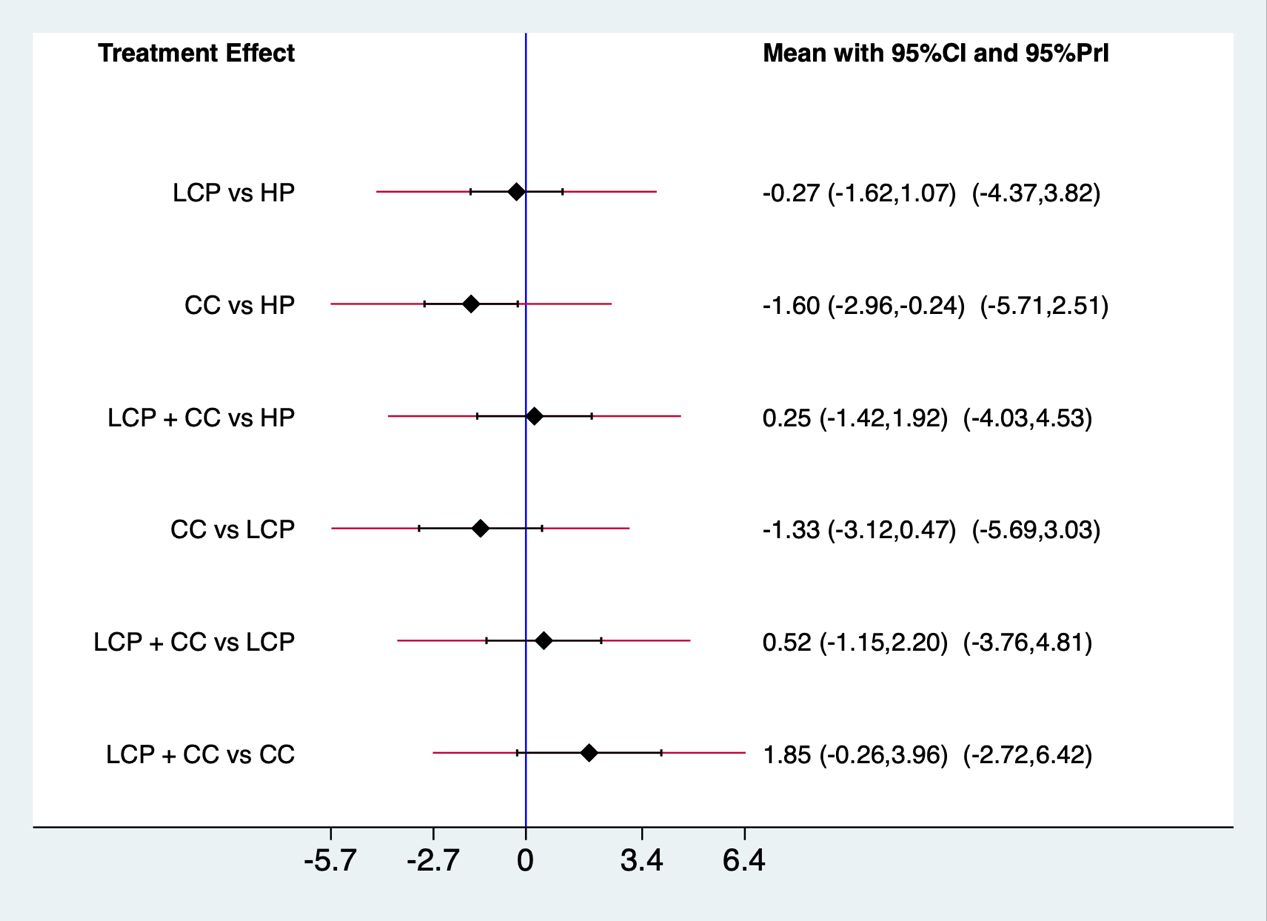
**

**Supplementary Figure 5K**

**
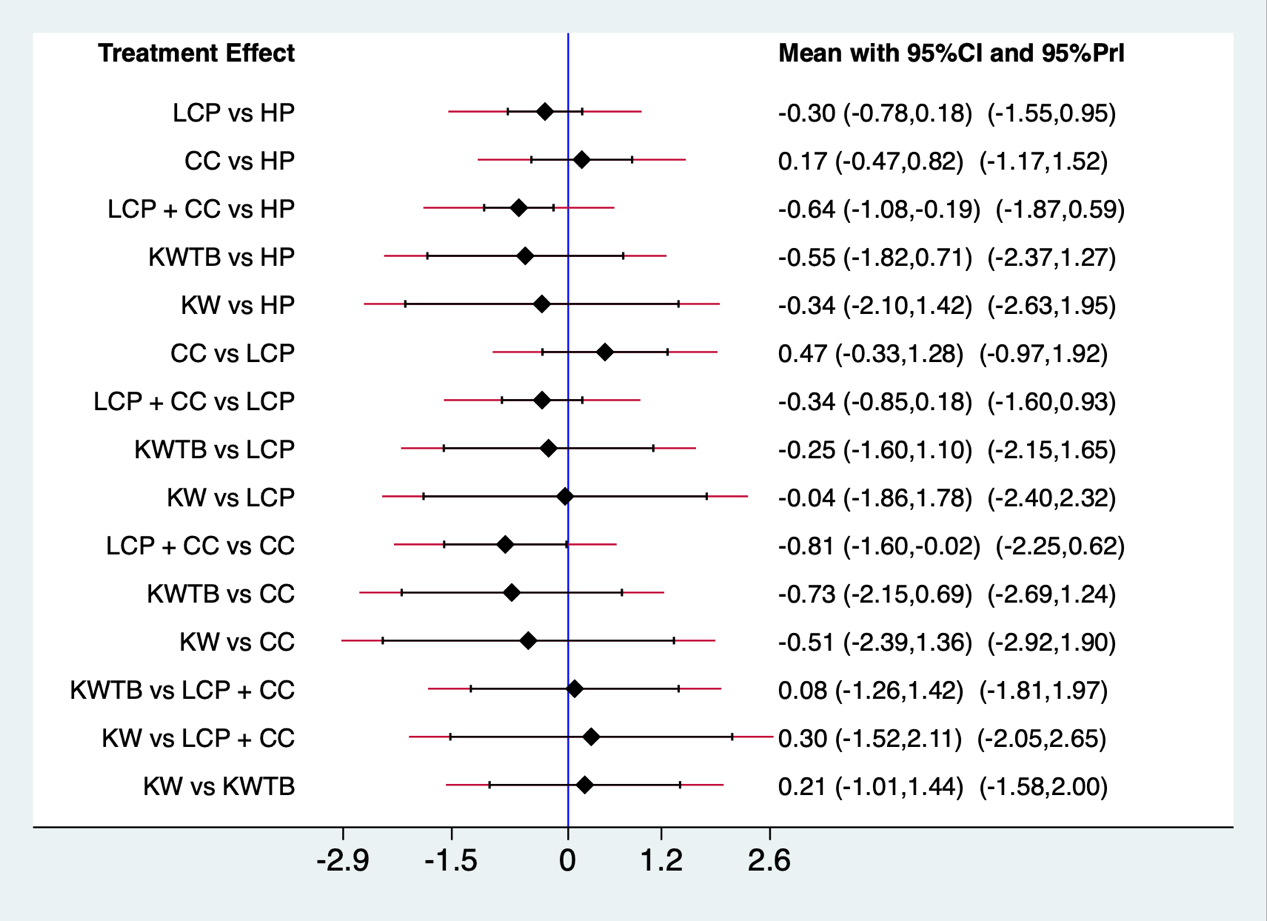
**
