# Supplementary material for: Different internal fixation methods for unstable distal clavicle fractures in adults: a systematic review and network meta-analysis
Source: J Orthop Surg Res. 2022 Jan 24;17:43. doi: 10.1186/s13018-021-02904-6 (PMC8785604; doi:10.1186/s13018-021-02904-6)
Supplement: Supplementary file 4 — Additional file 4: Table S1. The confidence rating for the CMS of different internal fixation comparisons for UDCFs. [file 13018_2021_2904_MOESM4_ESM.docx]

**Additional file 4: Table S1.** The confidence rating of CMS of different internal fixation comparisons for unstable distal clavicle fracture.

| **Comparison** | **No. of studies** | **Within-study bias** | **Reporting bias** | **Indirectness** | **Imprecision** | **Heterogeneity** | **Incoherence** | **Confidence rating** |
| --- | --- | --- | --- | --- | --- | --- | --- | --- |
| **CC:HP** | 5 | Some concerns | Low risk | No concerns | No concerns | Major concerns | No concerns | Low |
| **HP:KWTB** | 3 | Some concerns | Low risk | No concerns | No concerns | Major concerns | No concerns | Low |
| **HP:LCP** | 5 | Some concerns | Low risk | No concerns | No concerns | Major concerns | No concerns | Low |
| **HP:LCP + CC** | 6 | No concerns | Low risk | No concerns | No concerns | No concerns | No concerns | High |
| **KWTB + CC:LCP + CC** | 1 | No concerns | Low risk | No concerns | Major concerns | No concerns | No concerns | Low |
| **LCP:LCP + CC** | 5 | No concerns | Low risk | No concerns | No concerns | Major concerns | No concerns | Low |
| **CC:KWTB** | 0 | Some concerns | Low risk | No concerns | No concerns | Major concerns | No concerns | Low |
| **CC:KWTB + CC** | 0 | No concerns | Low risk | No concerns | Major concerns | No concerns | No concerns | Low |
| **CC:LCP** | 0 | Some concerns | Low risk | No concerns | Major concerns | No concerns | No concerns | Low |
| **CC:LCP + CC** | 0 | No concerns | Low risk | No concerns | Major concerns | No concerns | No concerns | Low |
| **HP:KWTB + CC** | 0 | No concerns | Low risk | No concerns | Major concerns | No concerns | No concerns | Low |
| **KWTB:KWTB + CC** | 0 | No concerns | Low risk | No concerns | Major concerns | No concerns | No concerns | Low |
| **KWTB:LCP** | 0 | Some concerns | Low risk | No concerns | No concerns | Major concerns | No concerns | Low |
| **KWTB:LCP + CC** | 0 | Some concerns | Low risk | No concerns | No concerns | No concerns | No concerns | Moderate |
| **KWTB + CC:LCP** | 0 | No concerns | Low risk | No concerns | Major concerns | No concerns | No concerns | Low |

HP, hook plate; LCP, locking compression plate; CC, coracoclavicular reconstruction; LCP + CC, combination of locking compression plate and coracoclavicular reconstruction; KWTB, Kirshner wire and tension band; KWTB + CC, combination of Kirshner wire and tension band and coracoclavicular reconstruction; KW, Kirshner wire
