# Supplementary material for: Different internal fixation methods for unstable distal clavicle fractures in adults: a systematic review and network meta-analysis
Source: J Orthop Surg Res. 2022 Jan 24;17:43. doi: 10.1186/s13018-021-02904-6 (PMC8785604; doi:10.1186/s13018-021-02904-6)

**Additional file 5: Figure S2.** Forest plots of the meta-analysis of different internal fixation methods for UDCFs. (A). UCLAs; (B). CCD; (C). Implant-related complications; (D). Reoperation; (E). Nonunion and delayed union; (F). Incision; (G). Operative time; (H). Blood loss; (I). Union time. 1, HP; 2, LCP; 3, CC; 4, LCP + CC; 5, KWTB; 6, KWTB + CC; 7, KW.

**Supplementary Figure 2A**


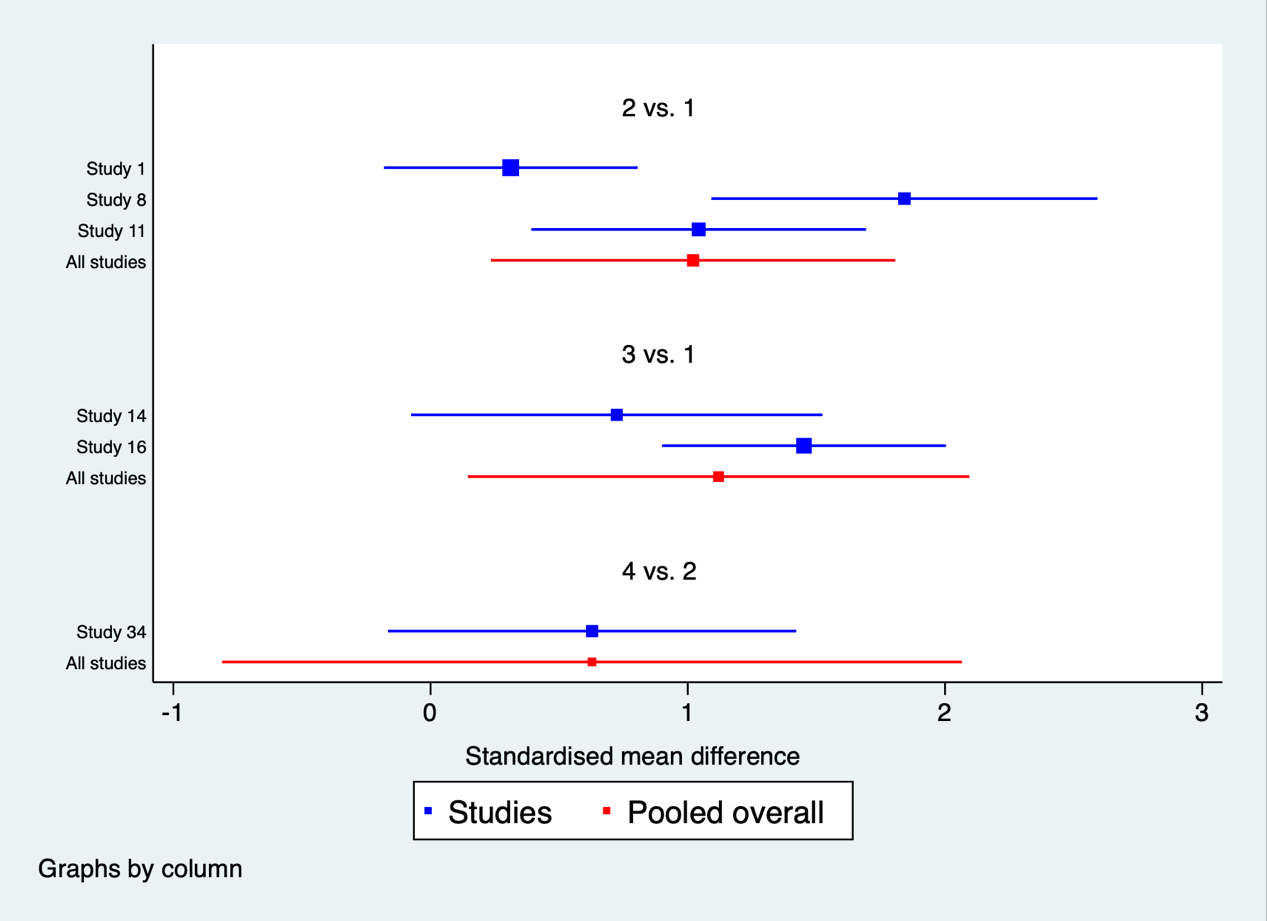


**Supplementary Figure 2B**


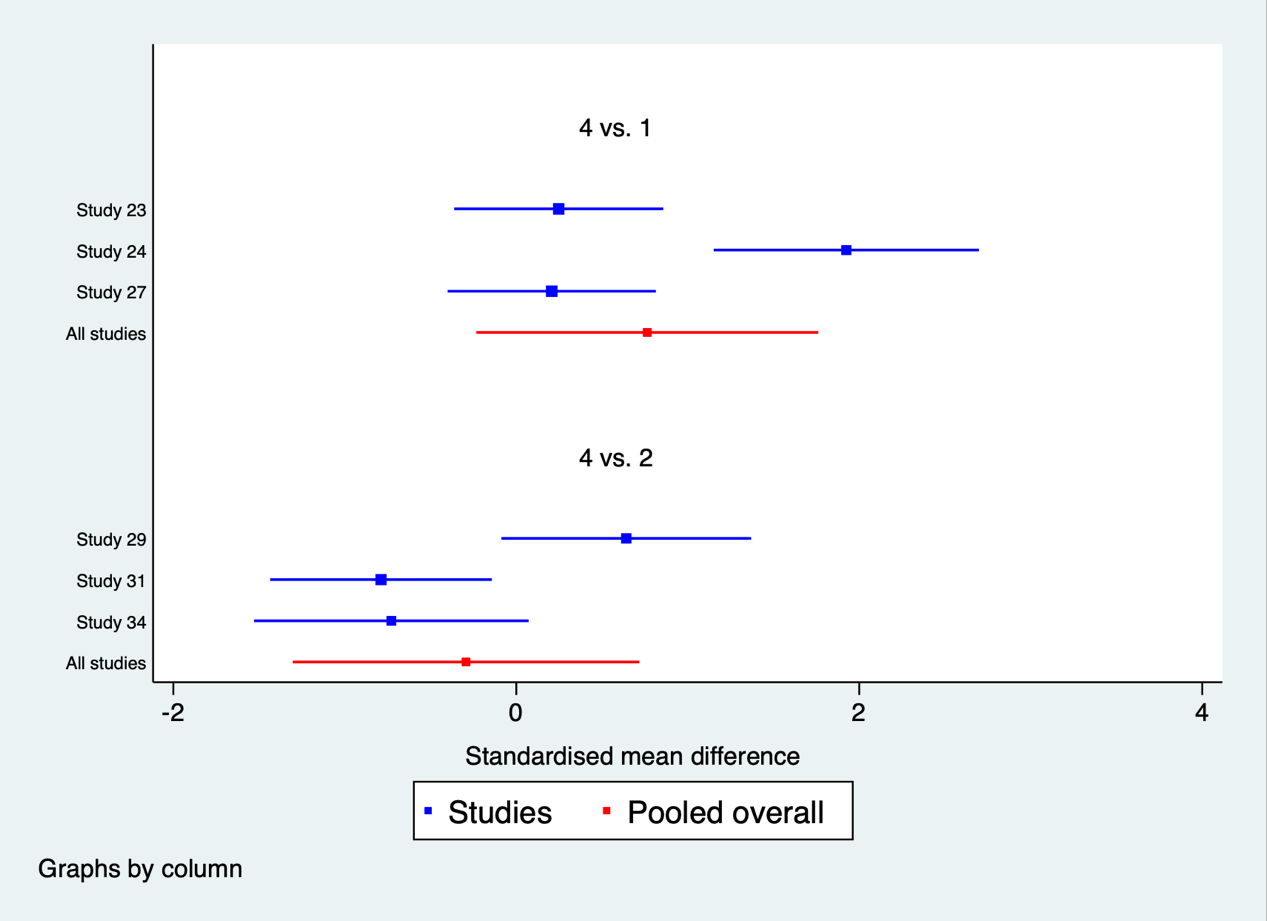


**Supplementary Figure 2C**


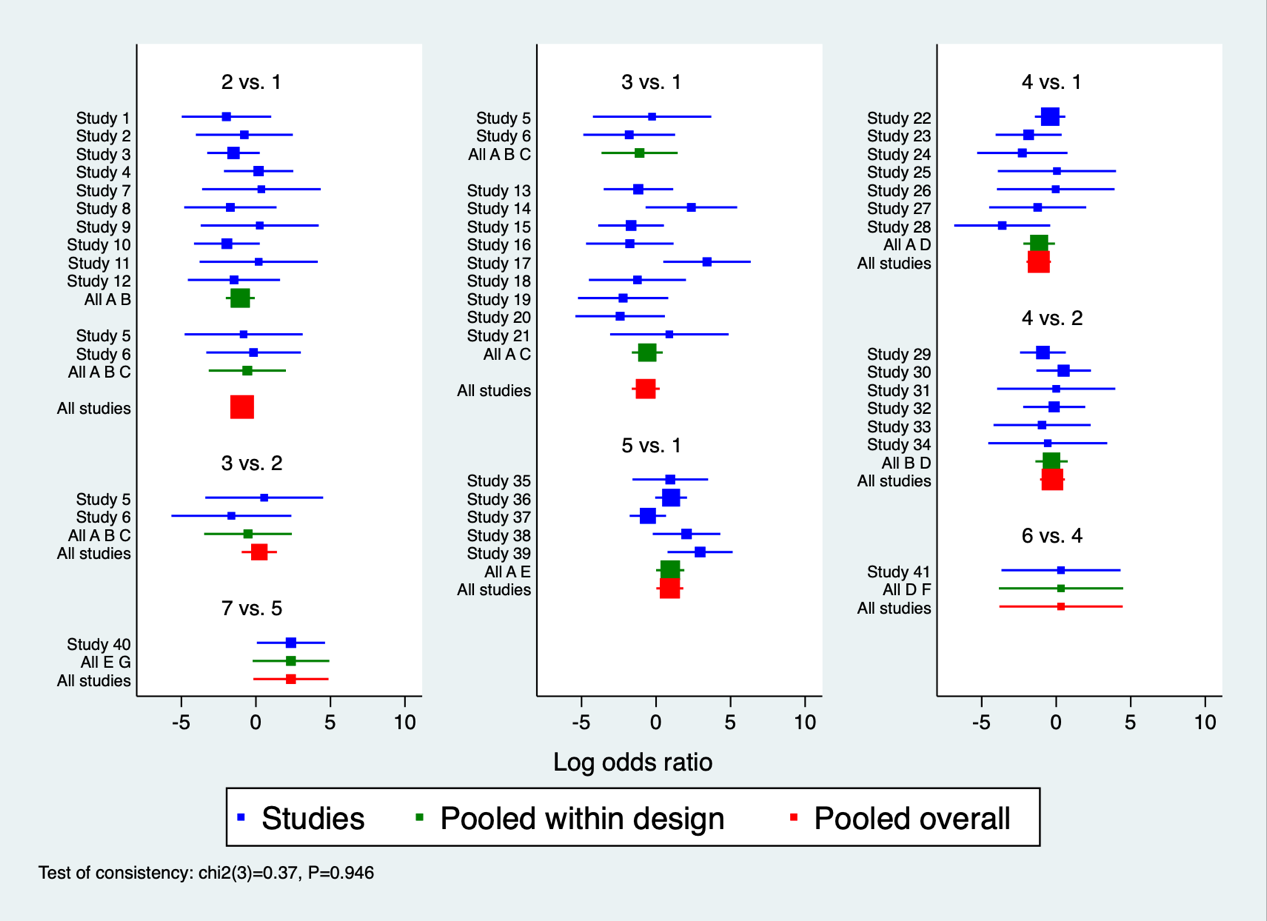


**Supplementary Figure 2D**

**
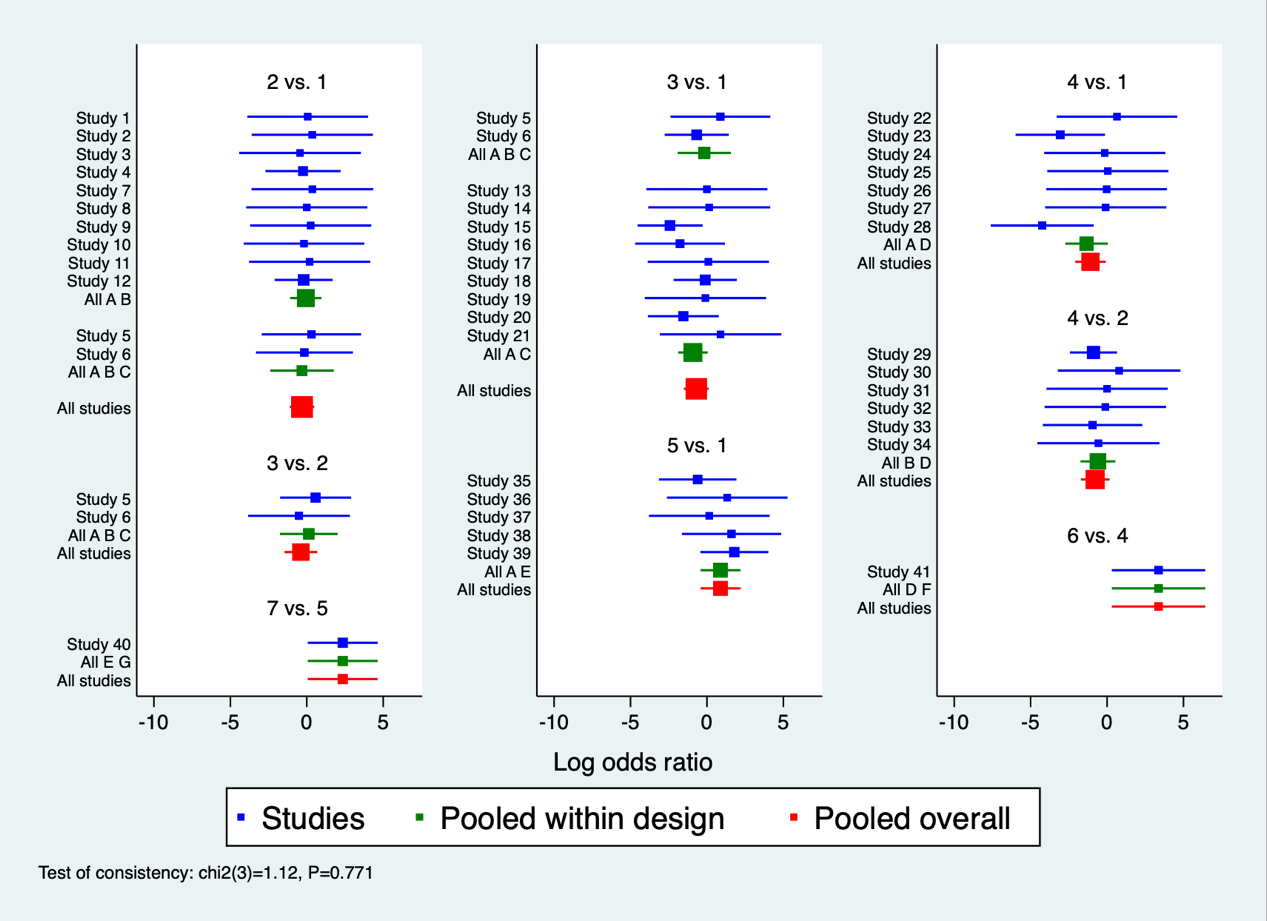
**

**Supplementary Figure 2E**


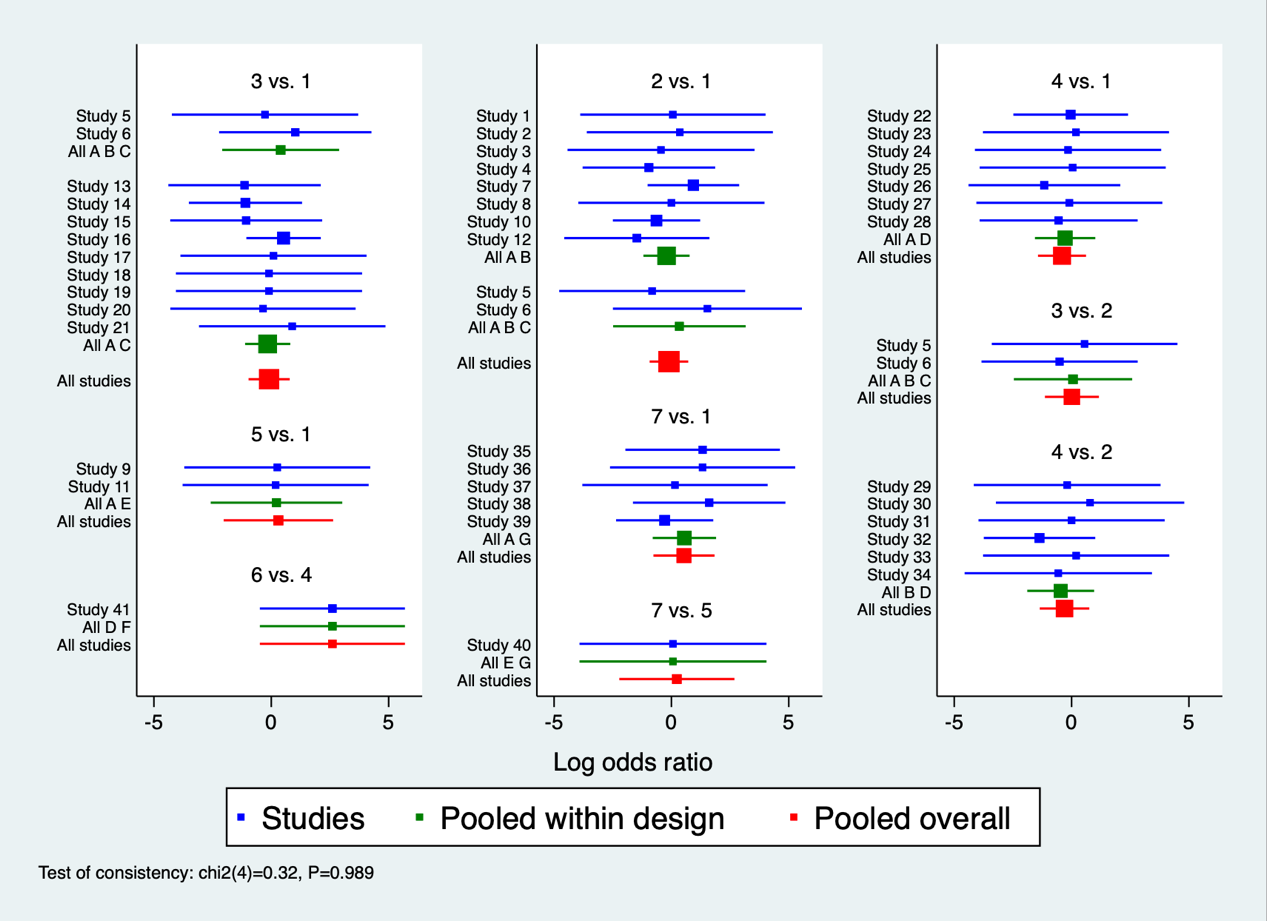


**Supplementary Figure 2F**


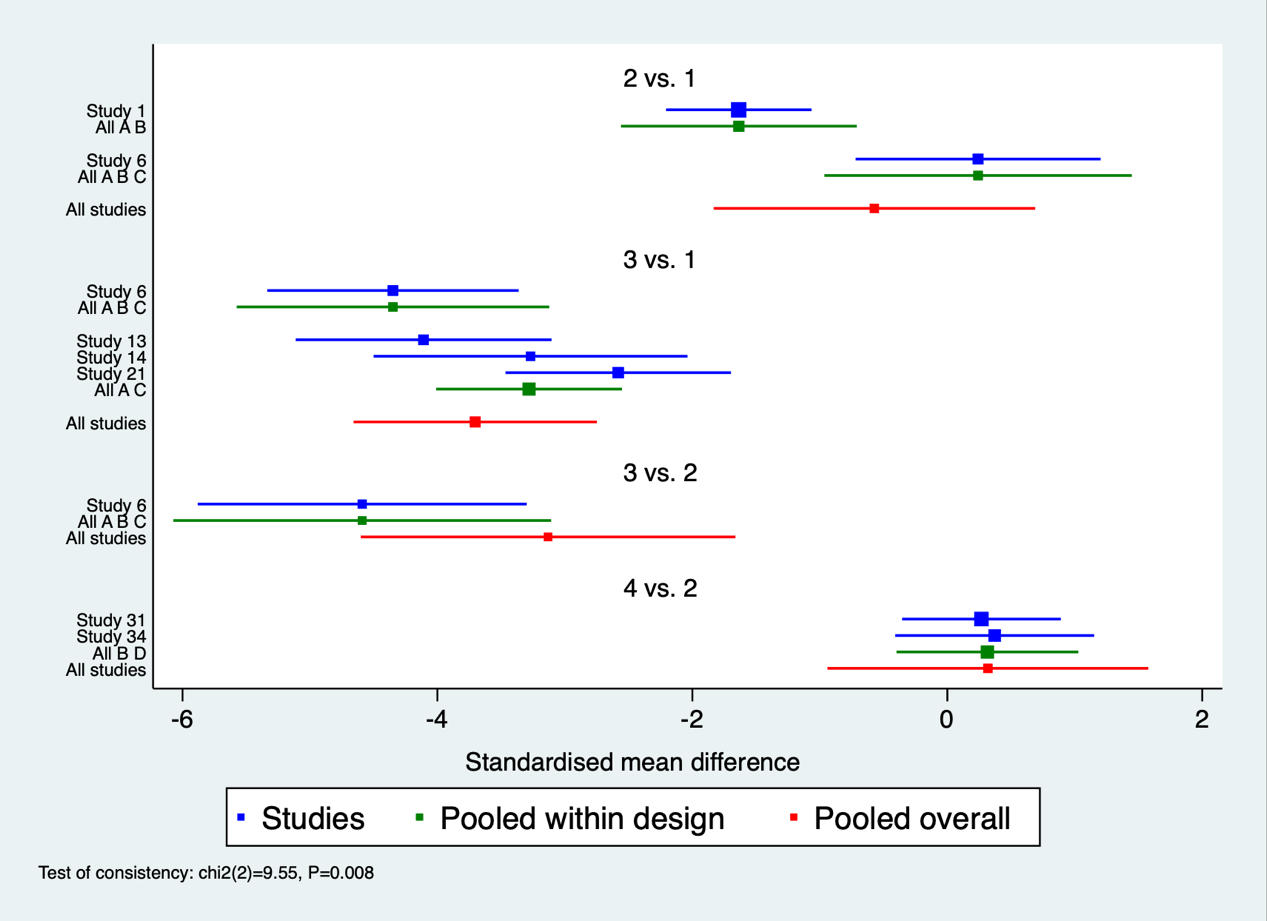


**Supplementary Figure 2G**


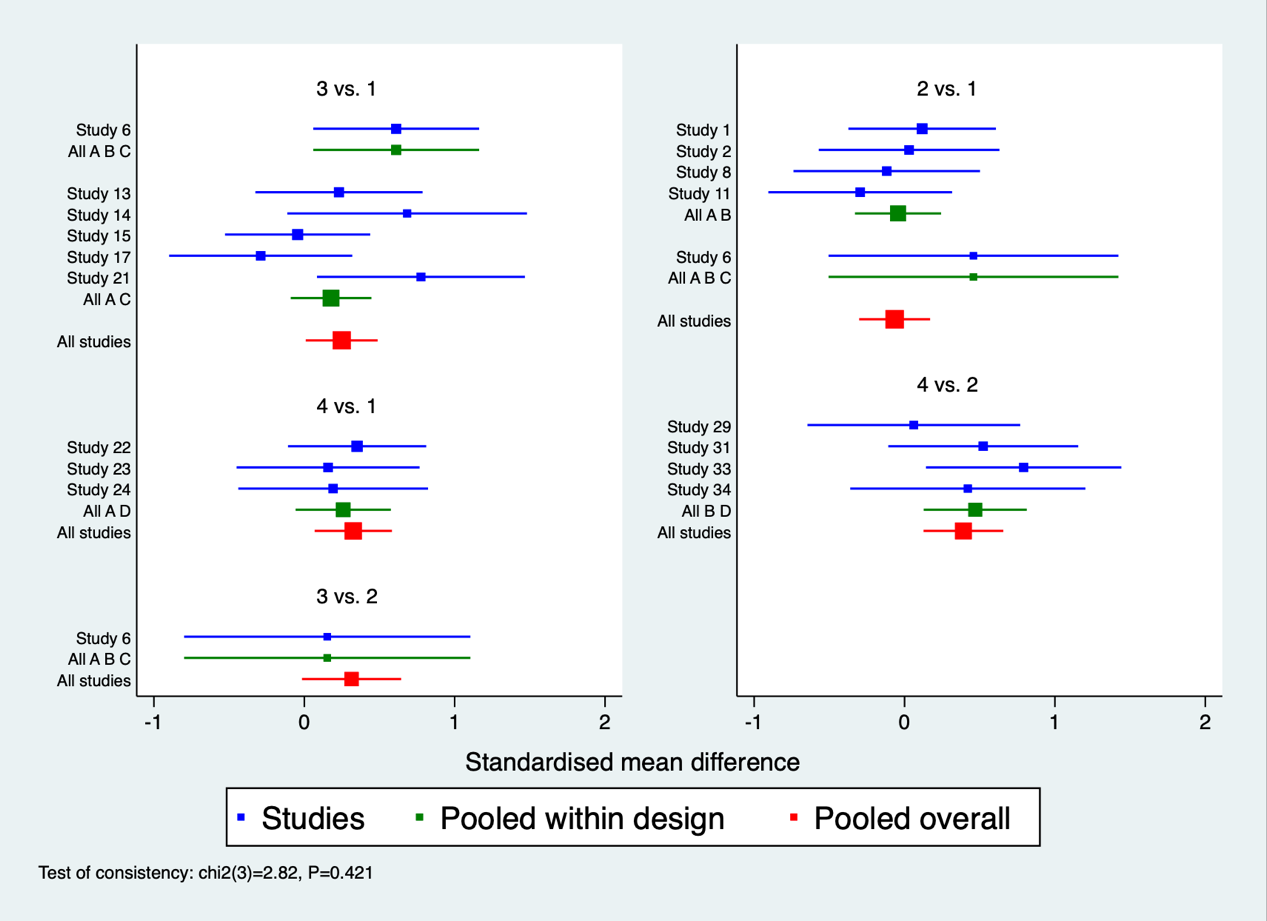


**Supplementary Figure 2H**


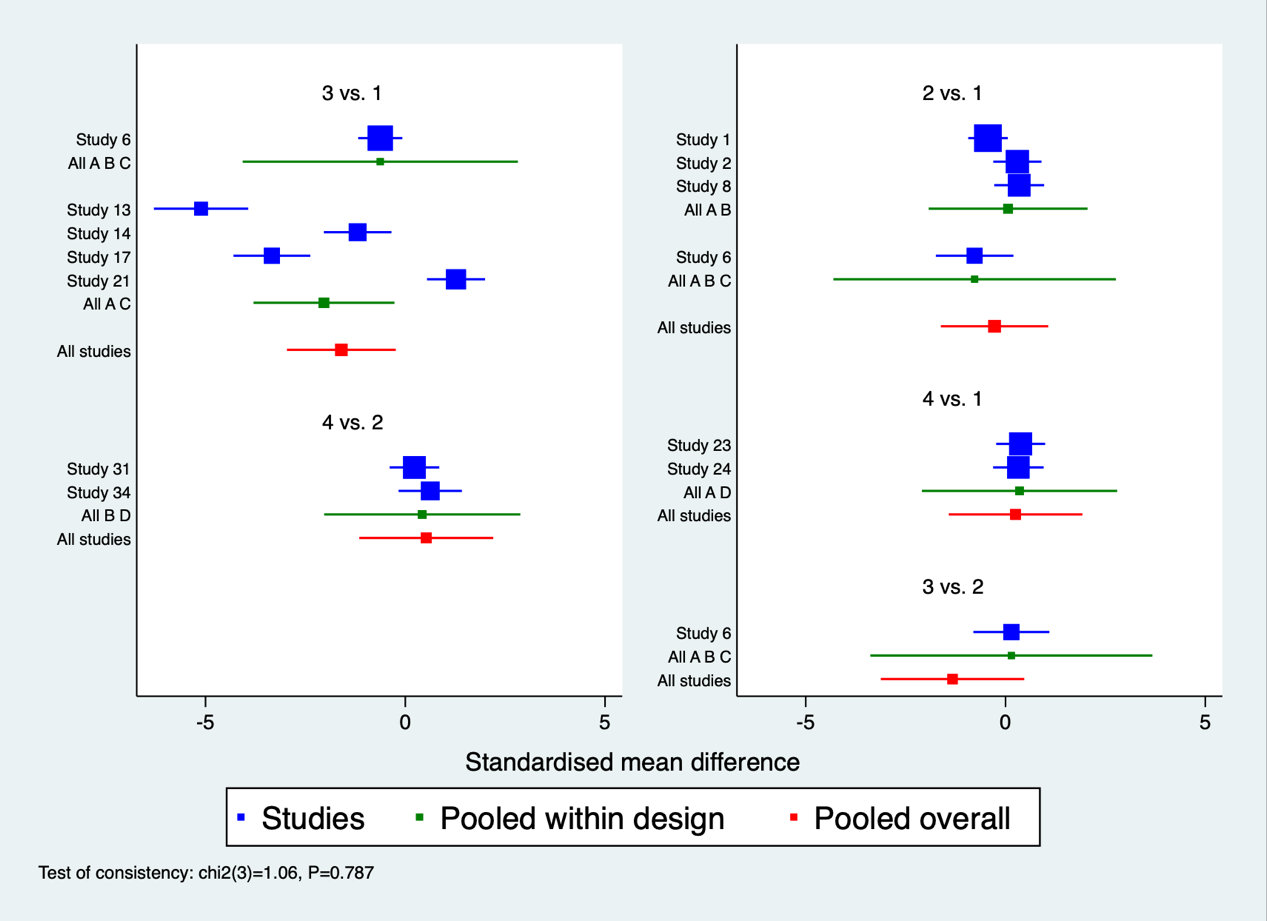


**Supplementary Figure 2I**


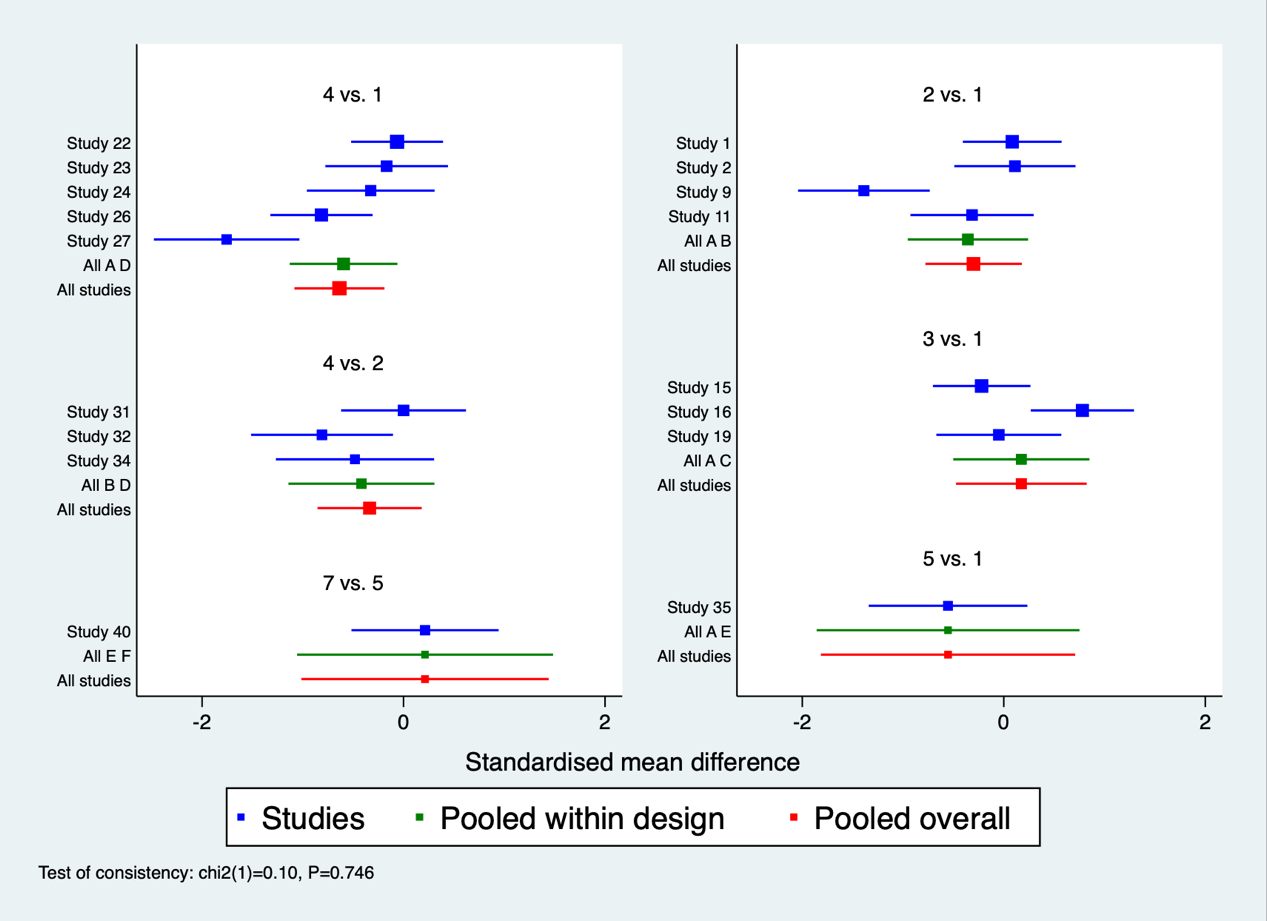

Supplement: Supplementary file 5 — Additional file 5: Fig. S2. Forest plots of the meta-analysis of different internal fixation methods for UDCFs. A. UCLAs; B CCD; CImplant-related complications; D Reoperation; E Nonunion and delayed union; F Incision; G Operative time; H Blood loss; I Union time. 1, HP; 2, LCP; 3, CC; 4, LCP + CC; 5, KWTB; 6, KWTB + CC; 7, KW. [file 13018_2021_2904_MOESM5_ESM.docx]
