# Supplementary material for: Different internal fixation methods for unstable distal clavicle fractures in adults: a systematic review and network meta-analysis
Source: J Orthop Surg Res. 2022 Jan 24;17:43. doi: 10.1186/s13018-021-02904-6 (PMC8785604; doi:10.1186/s13018-021-02904-6)

**Additional file 6: Figure S3.** Network meta-analysis maps of the outcomes. (A). UCLAs; (B). CCD; (C). Implant-related complications; (D). Reoperation; (E). Nonunion and delayed union; (F). Incision; (G). Operative time; (H). Blood loss; (I). Union time. Each node represents an intervention, and the size of the node is proportional to the number of patients assigned to the intervention. The lines indicate direct comparisons between nodes, and the size of the line is proportional to the number of trials comparing each pair of nodes.

**Supplementary Figure 3A**


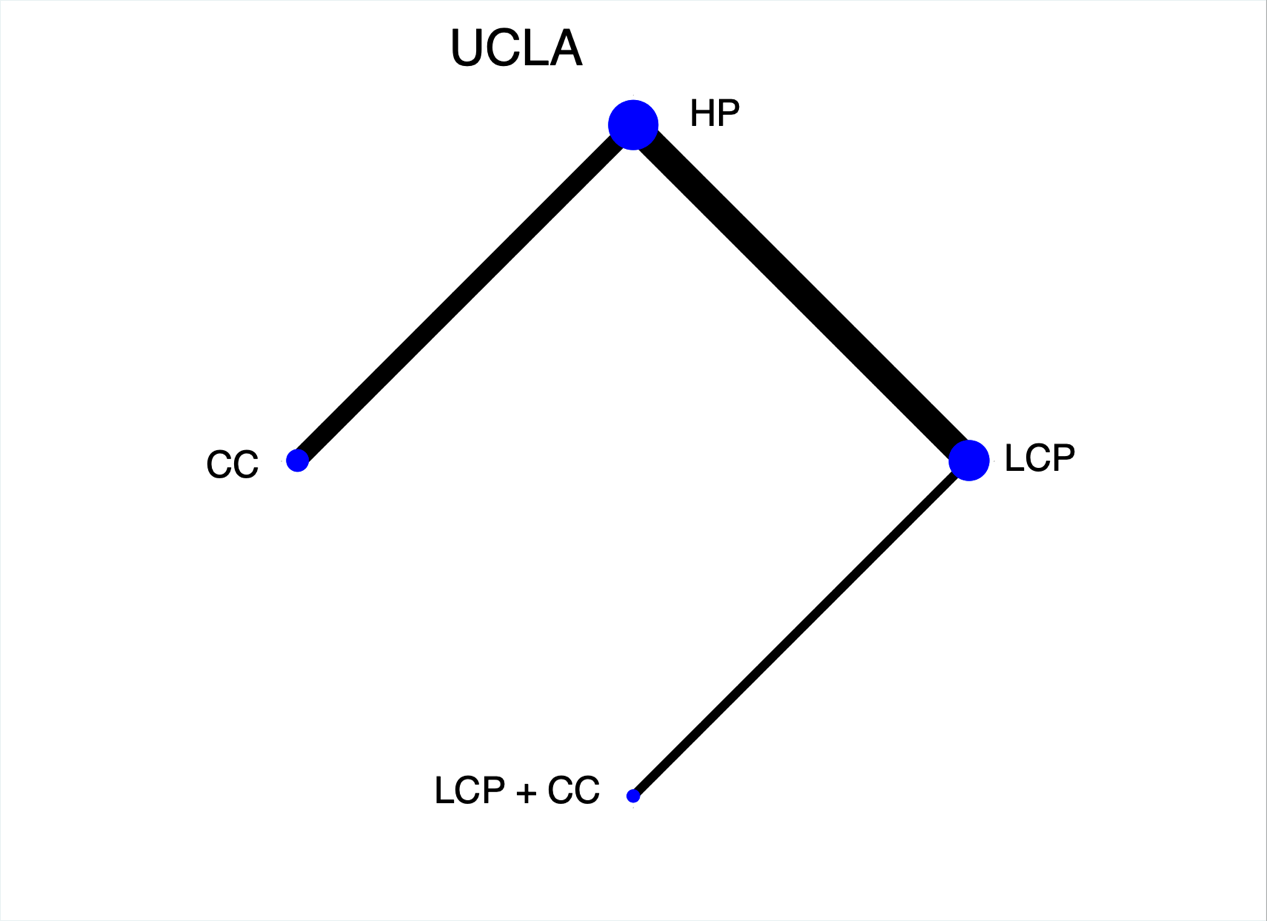


**Supplementary Figure 3B**


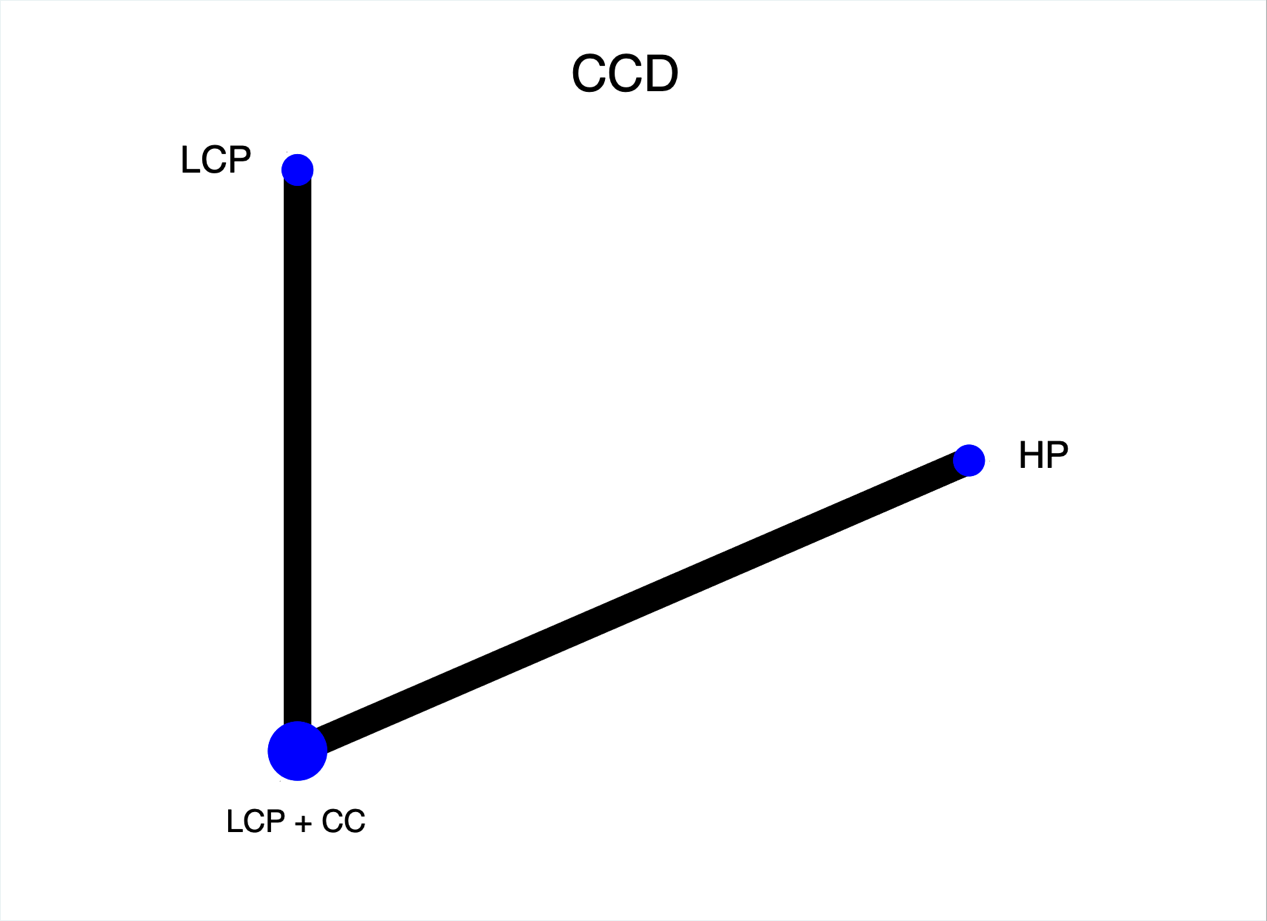


**Supplementary Figure 3C**


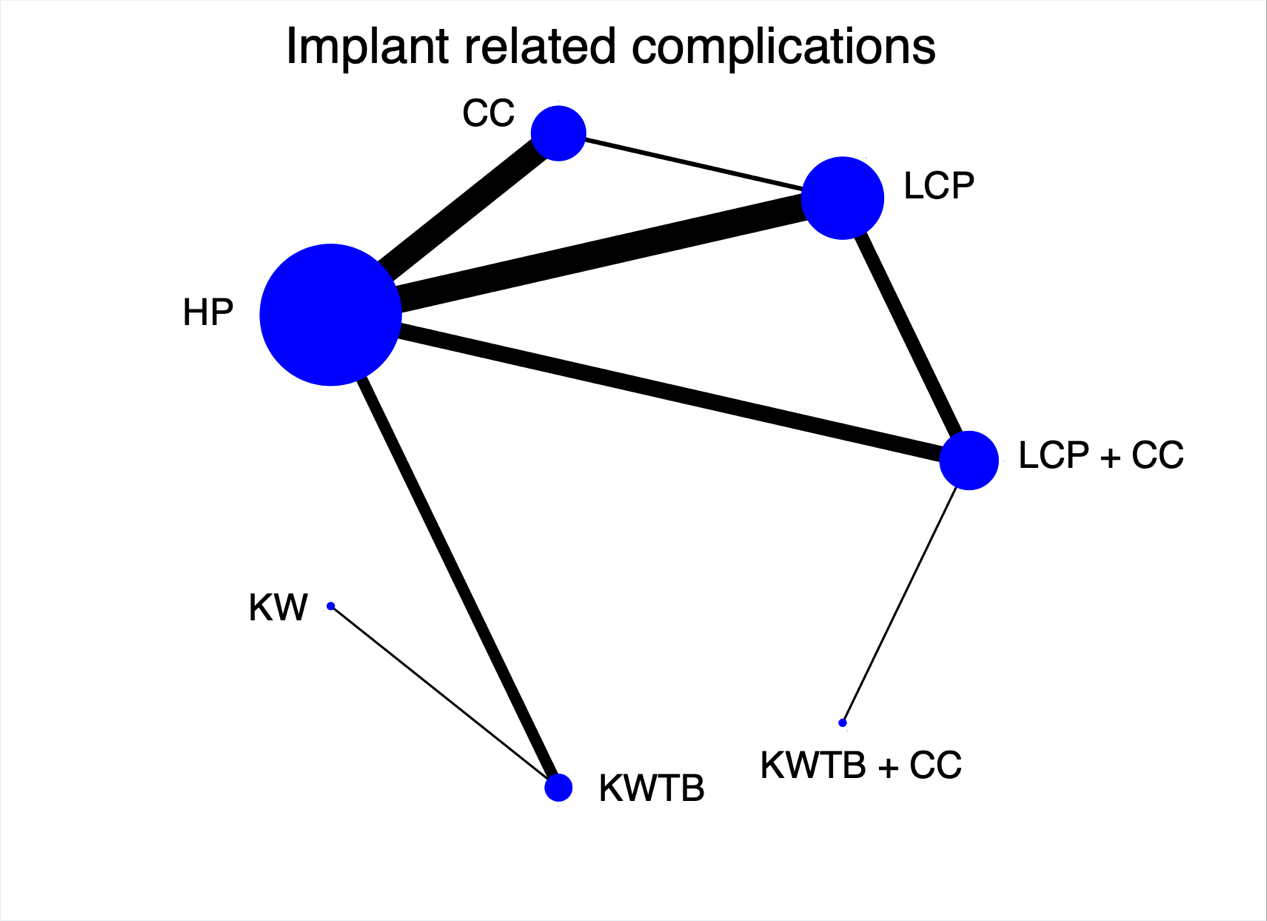


**Supplementary Figure 3D**


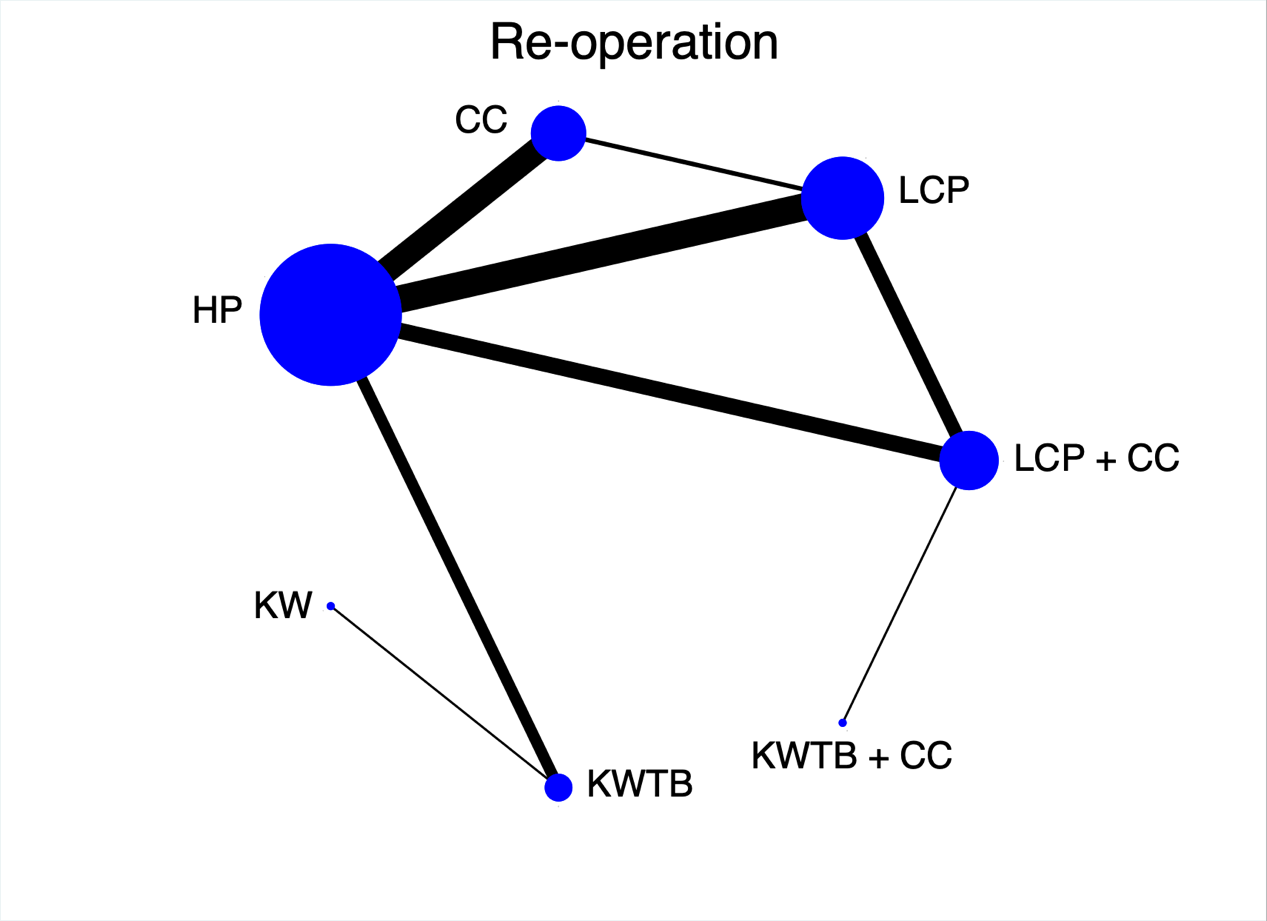


**Supplementary Figure 3E**


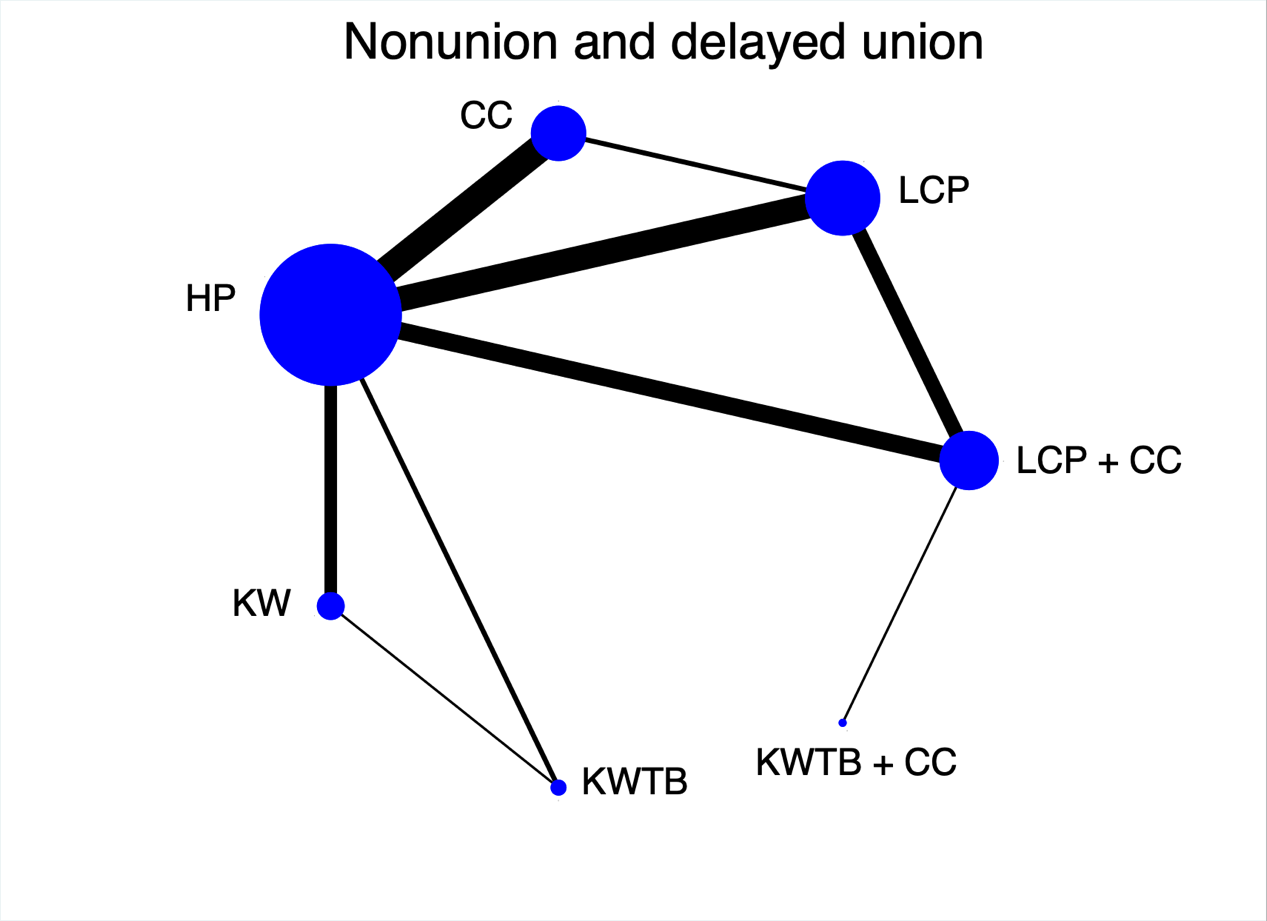


**Supplementary Figure 3F**


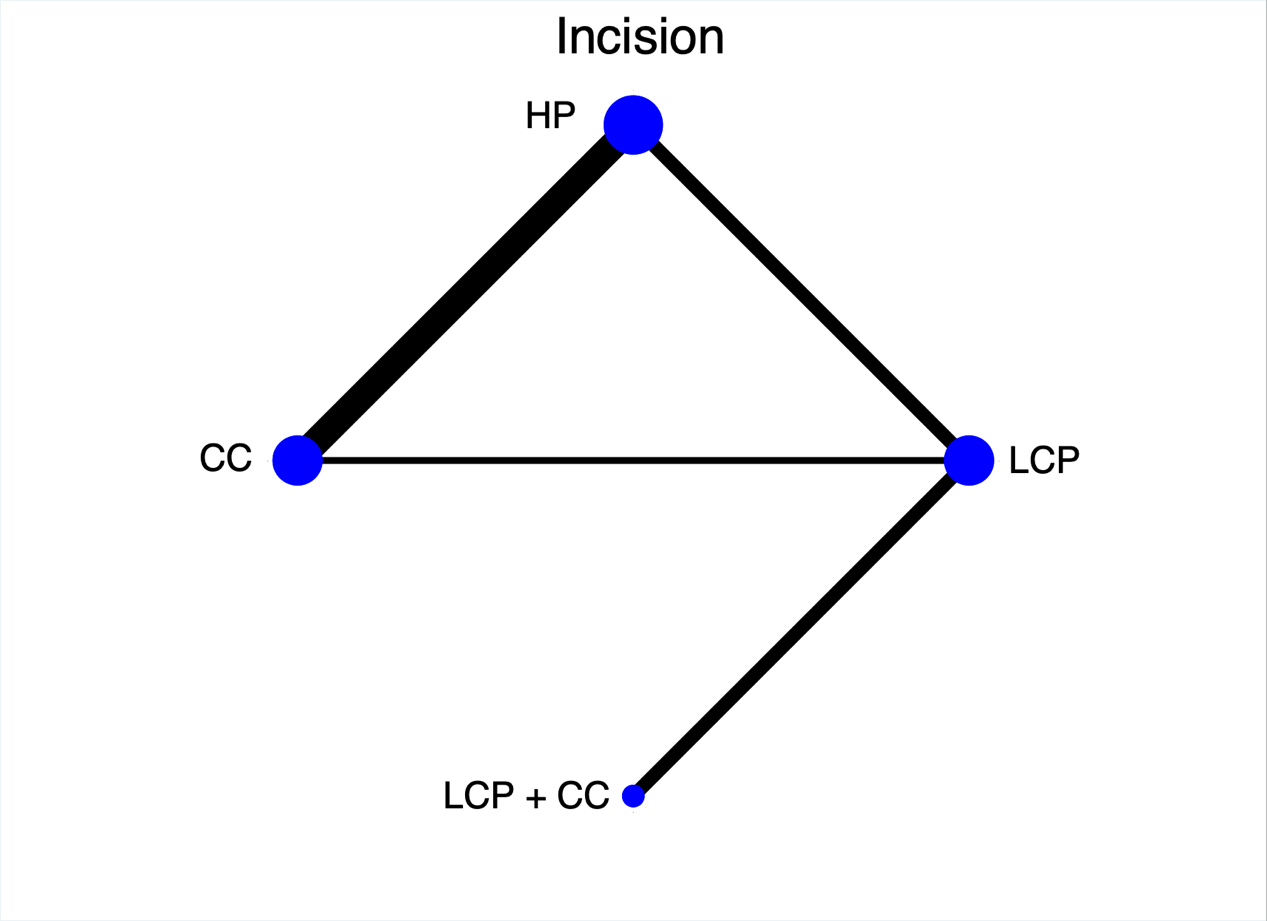


**Supplementary Figure 3G**


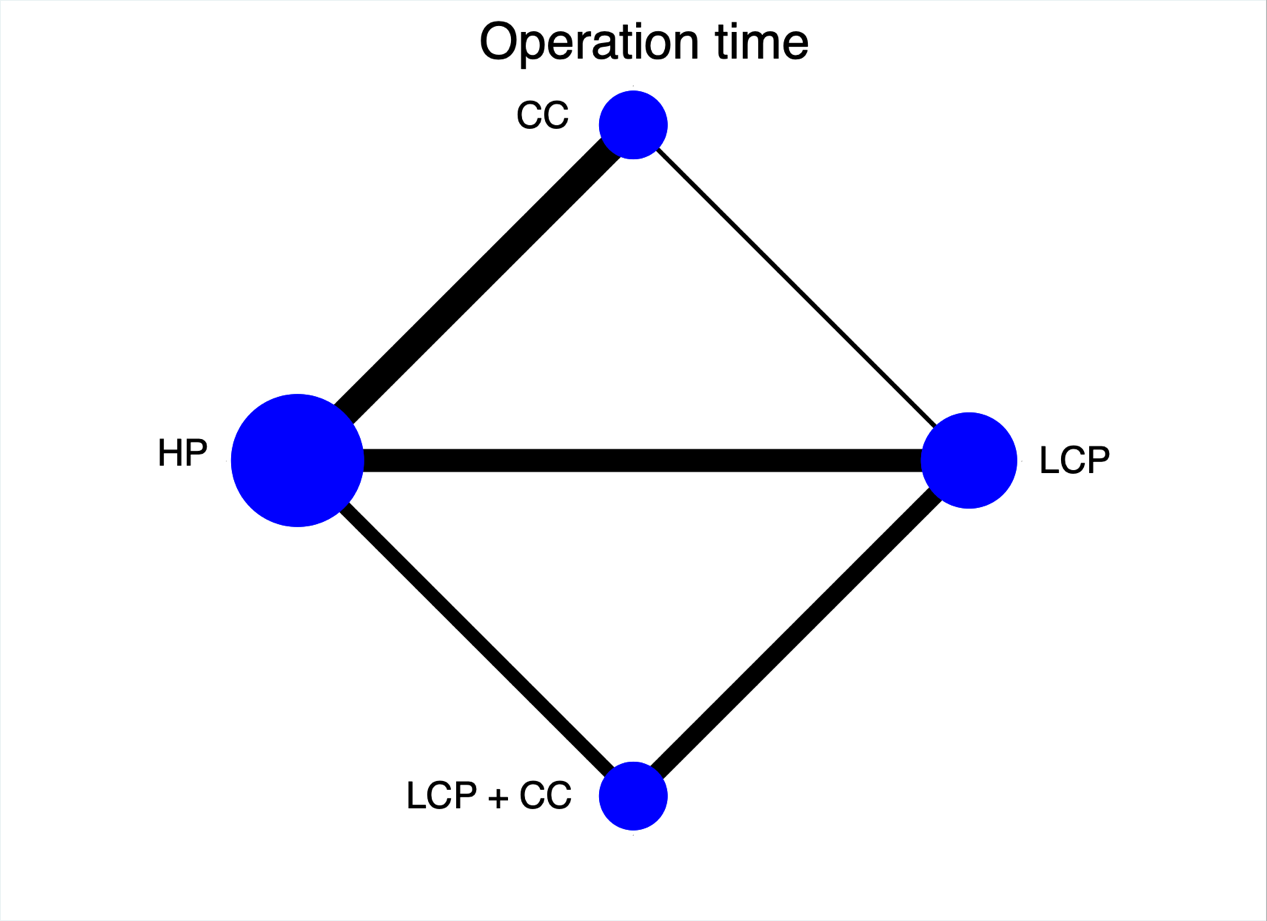


**Supplementary Figure 3H**

**
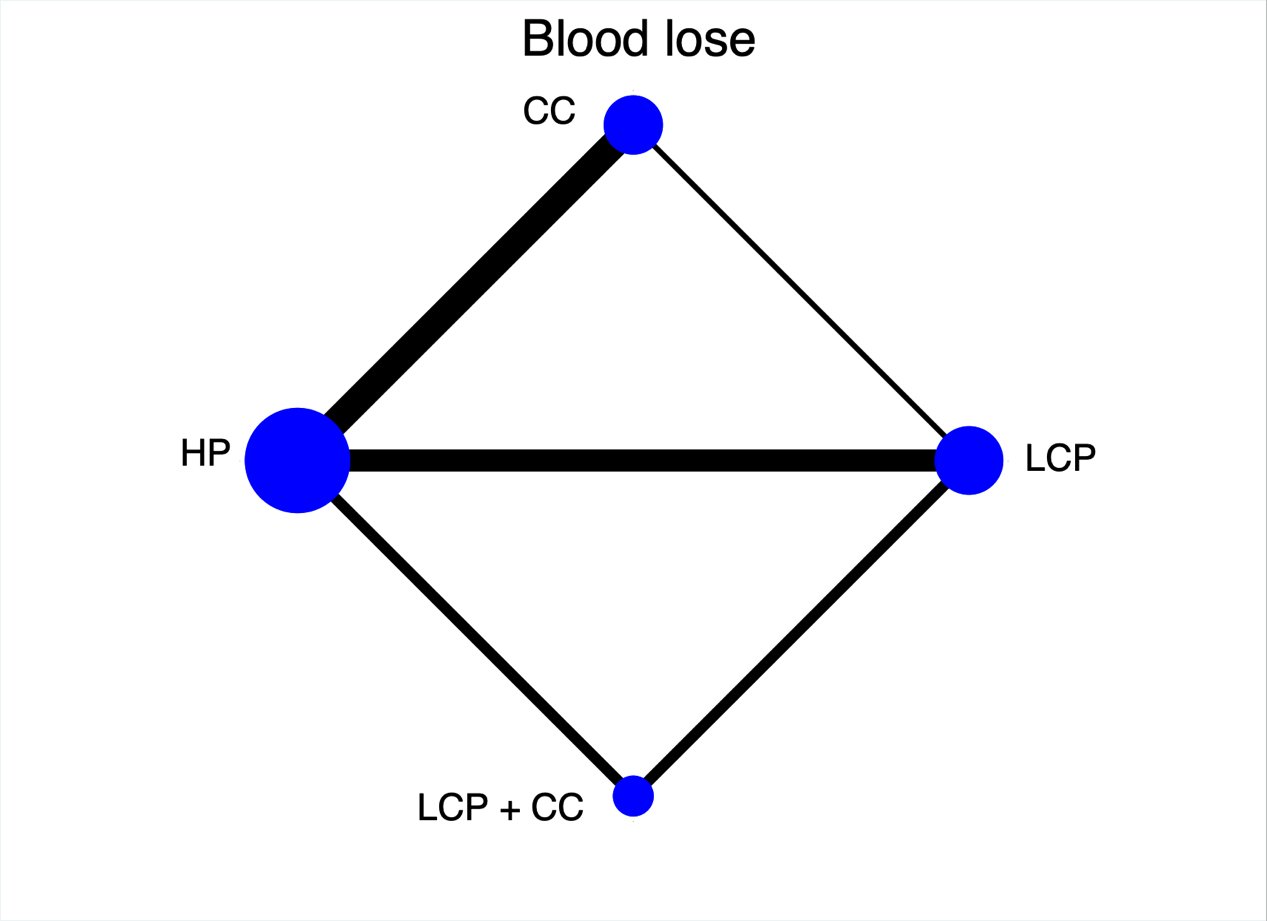
**

**Supplementary Figure 3I**


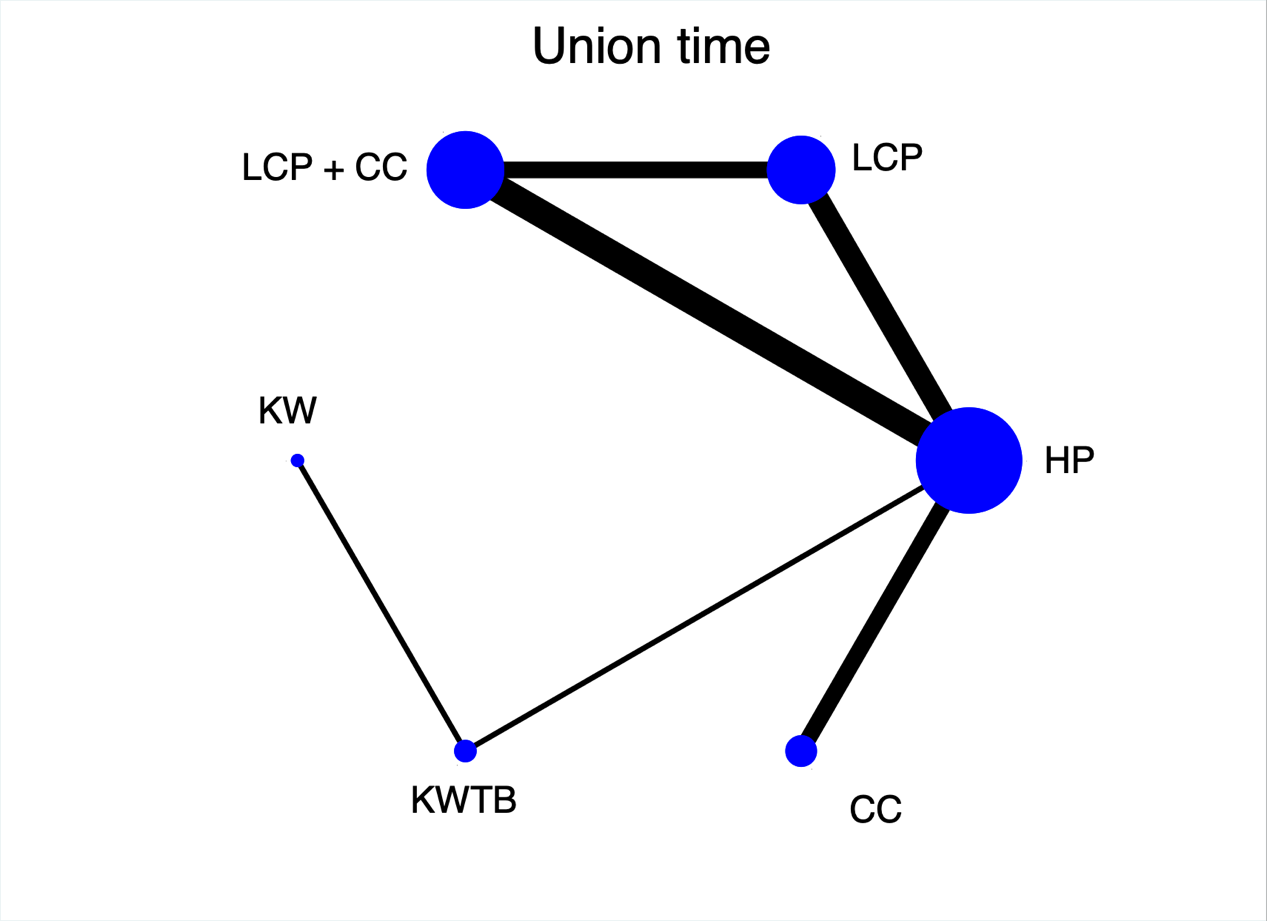

Supplement: Supplementary file 6 — Additional file 6: Fig. S3. Network meta-analysis maps of the outcomes. A UCLAs; B CCD; C Implant-related complications; D Reoperation; E Nonunion and delayed union; F Incision; G Operative time; H Blood loss; I Union time. Each node represents an intervention, and the size of the node is proportional to the number of patients assigned to the intervention. The lines indicate direct comparisons between nodes, and the size of the line is proportional to the number of trials comparing each pair of nodes. [file 13018_2021_2904_MOESM6_ESM.docx]
