# Supplementary material for: Different internal fixation methods for unstable distal clavicle fractures in adults: a systematic review and network meta-analysis
Source: J Orthop Surg Res. 2022 Jan 24;17:43. doi: 10.1186/s13018-021-02904-6 (PMC8785604; doi:10.1186/s13018-021-02904-6)

**Additional file 7: Figure S4.** Rankogram of different internal fixation methods for outcomes. (A). UCLAs; (B). CCD; (C). Implant-related complications; (D). Reoperation; (E). Nonunion and delayed union; (F). Incision; (G). Operative time; (H). Blood loss; (I). Union time.

**Supplementary Figure 4A**

**
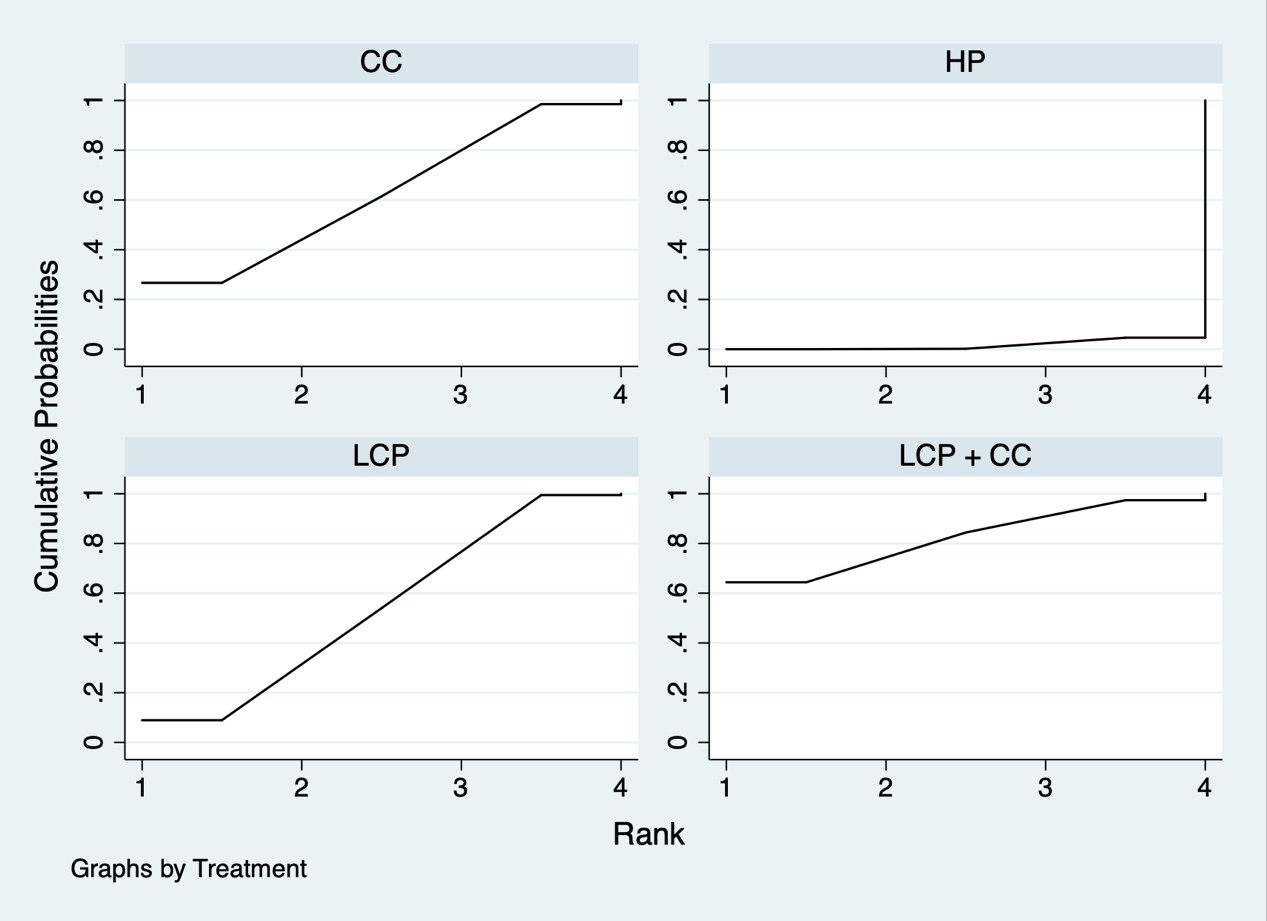
**

**Supplementary Figure 4B**

**
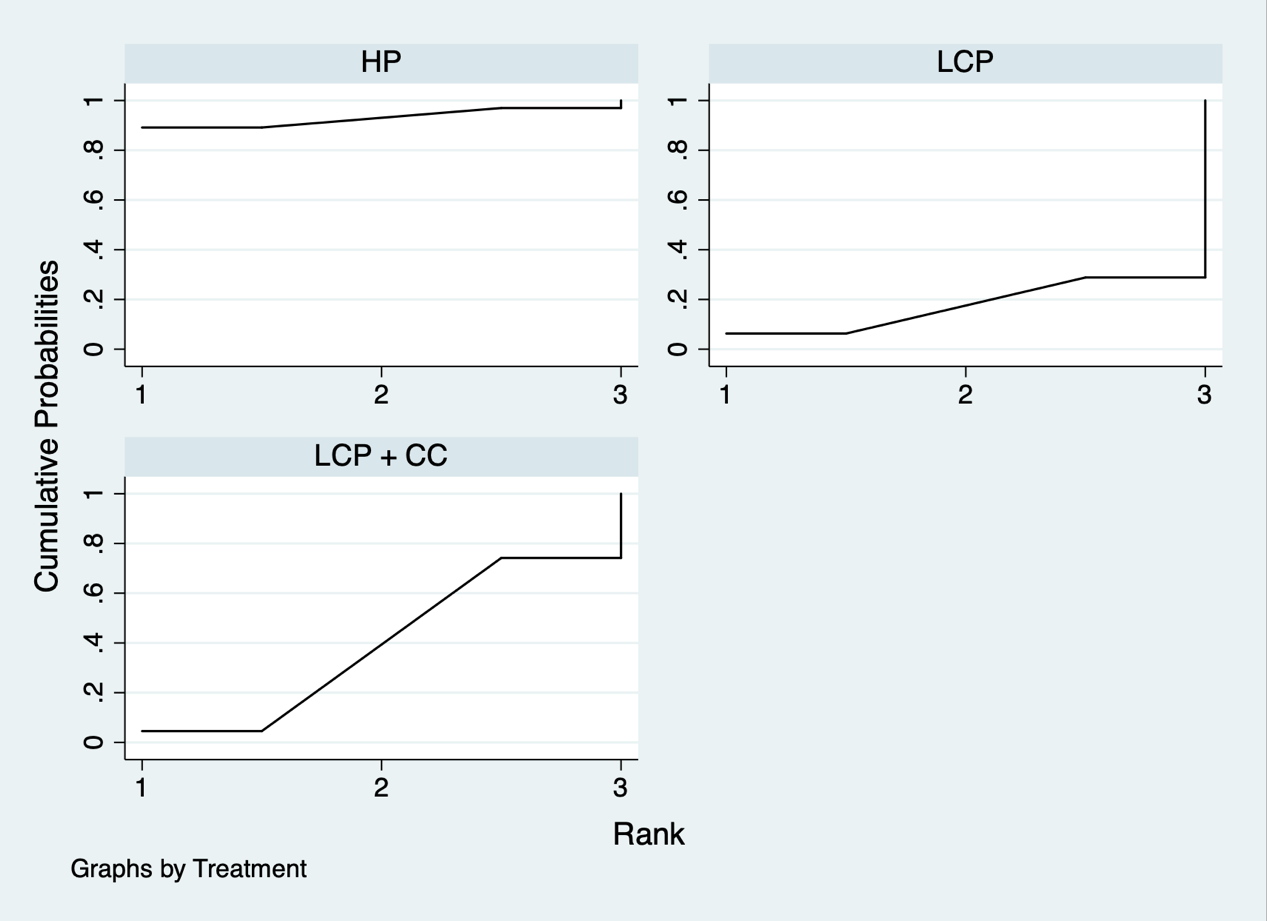
**

**Supplementary Figure 4C**

**
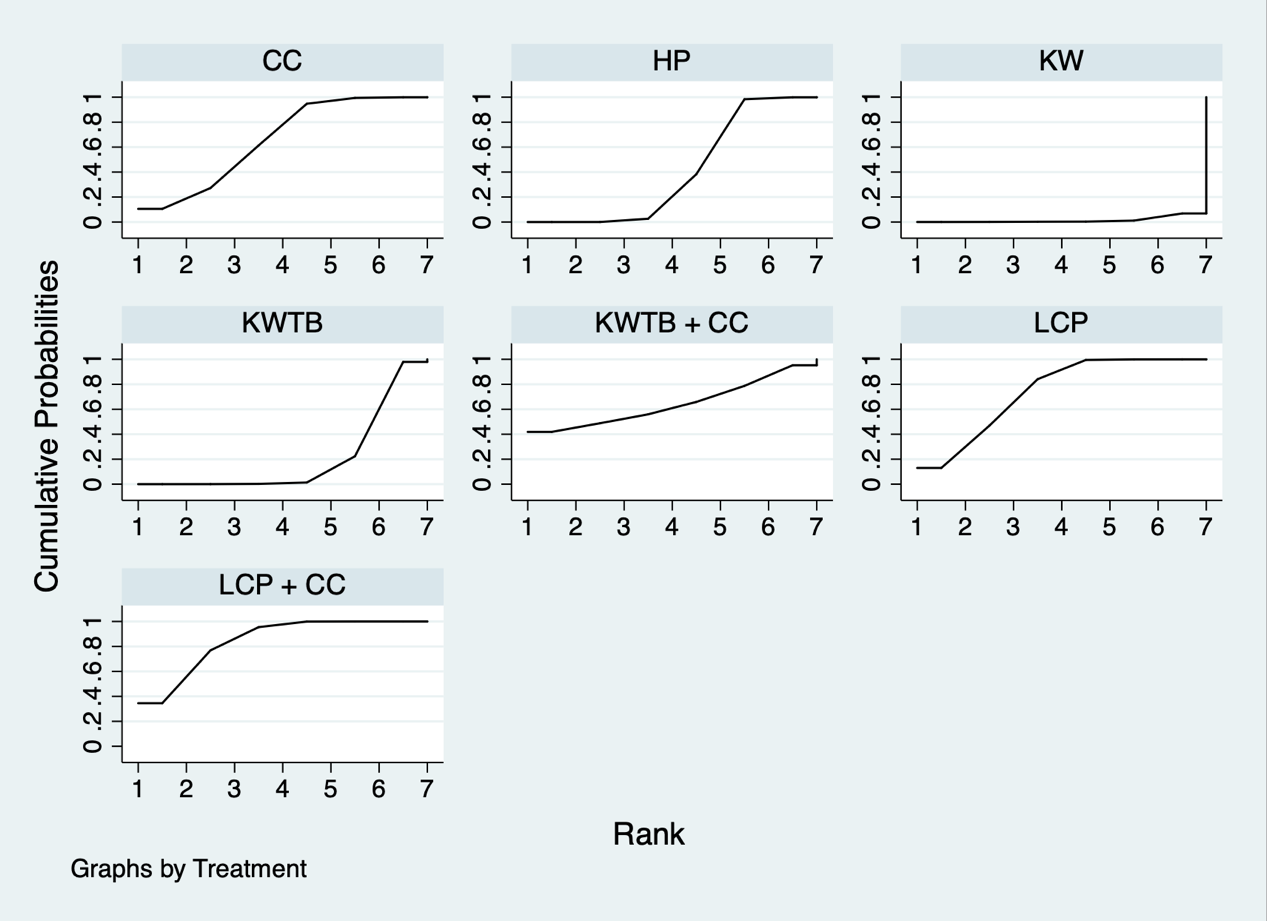
**

**Supplementary Figure 4D**

**
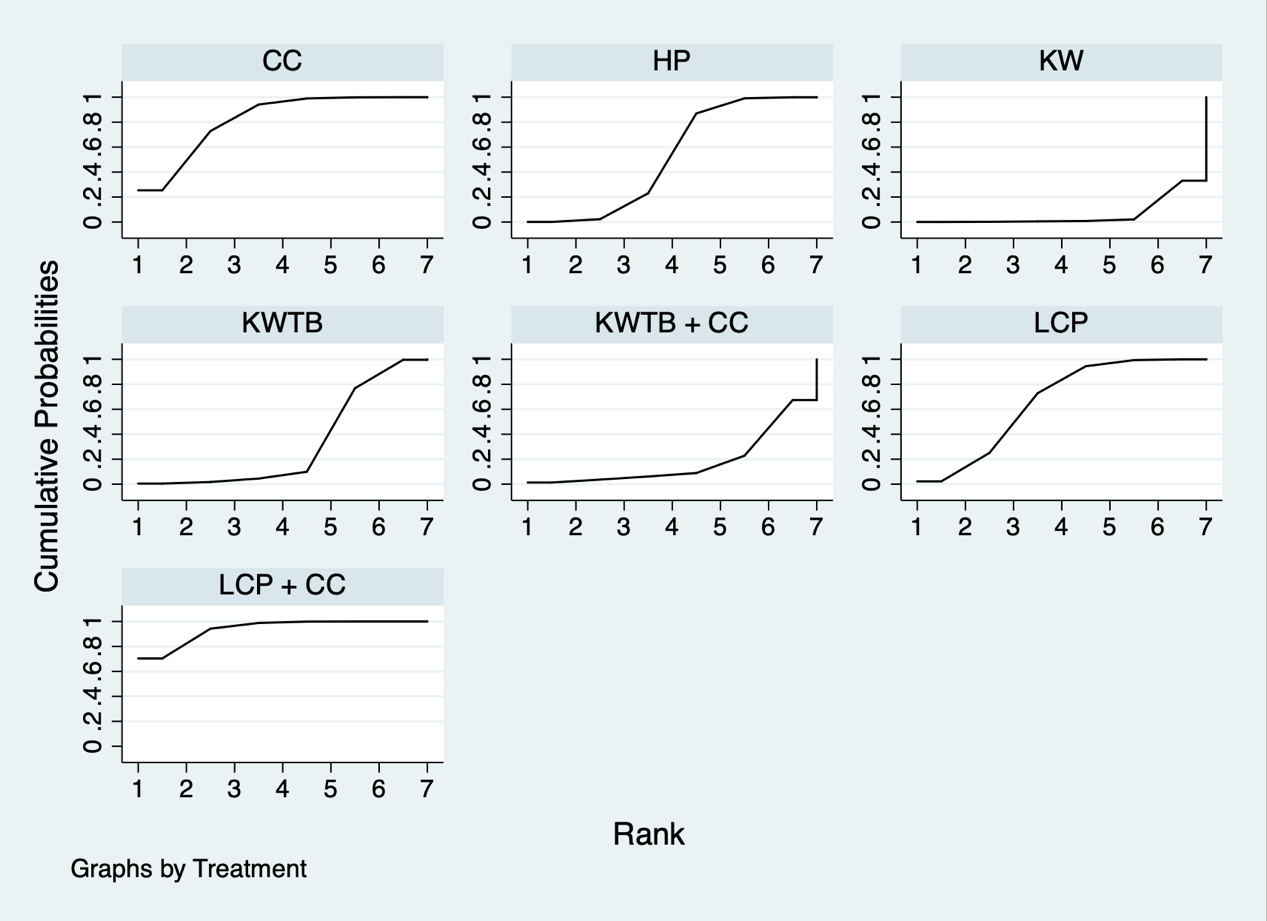
**

**Supplementary Figure 4E**

**
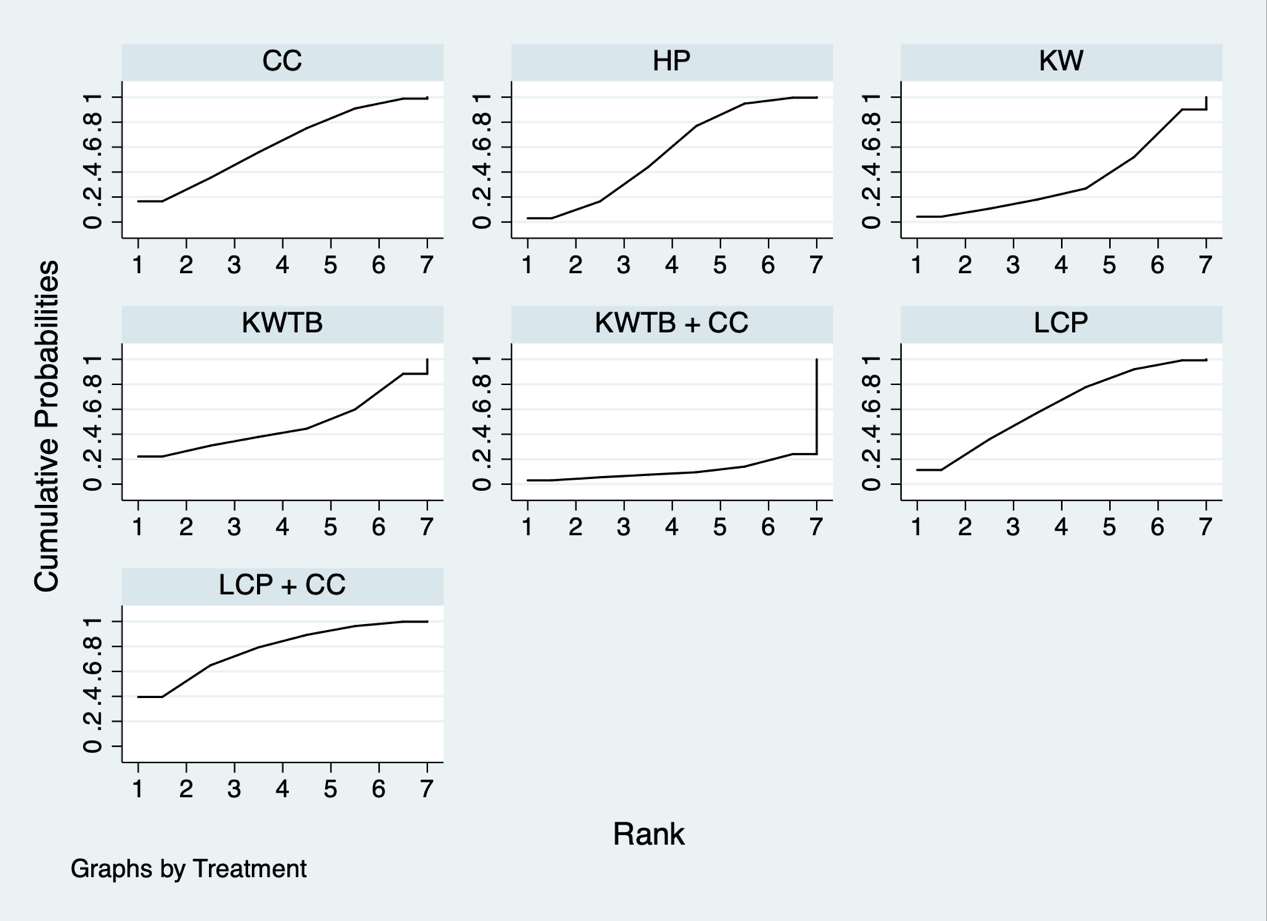
**

**Supplementary Figure 4F**

**
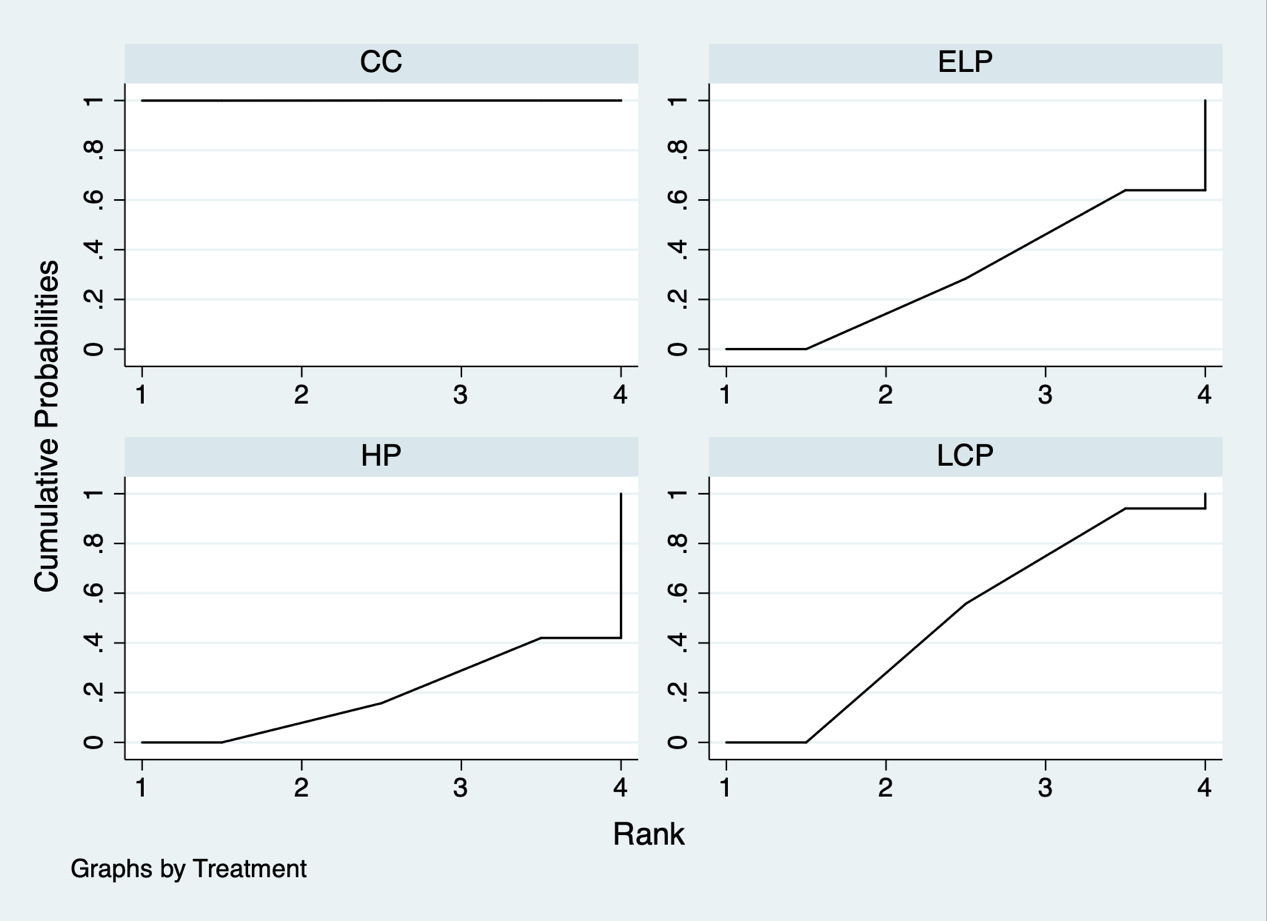
**

**Supplementary Figure 4G**

**
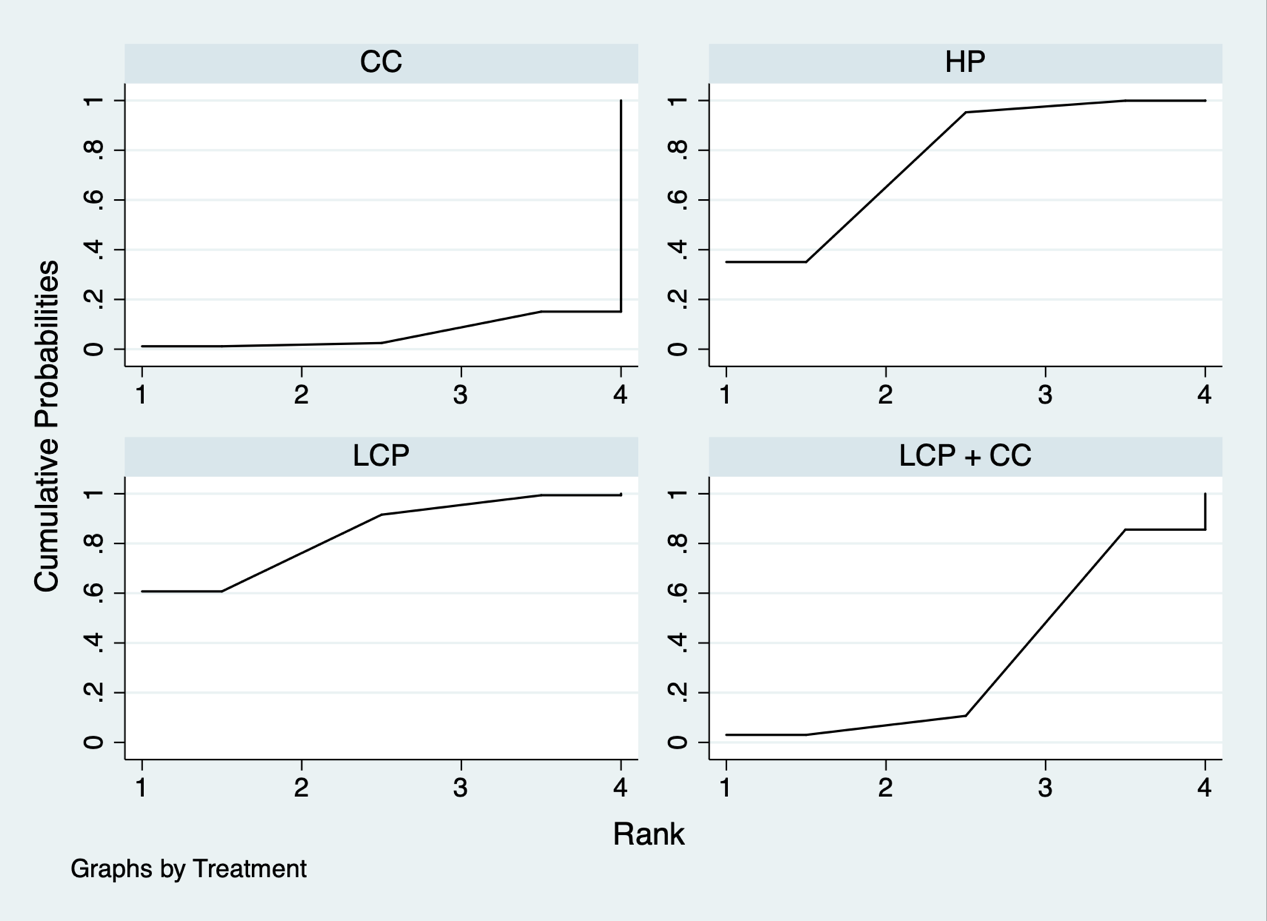
**

**Supplementary Figure 4H**

**
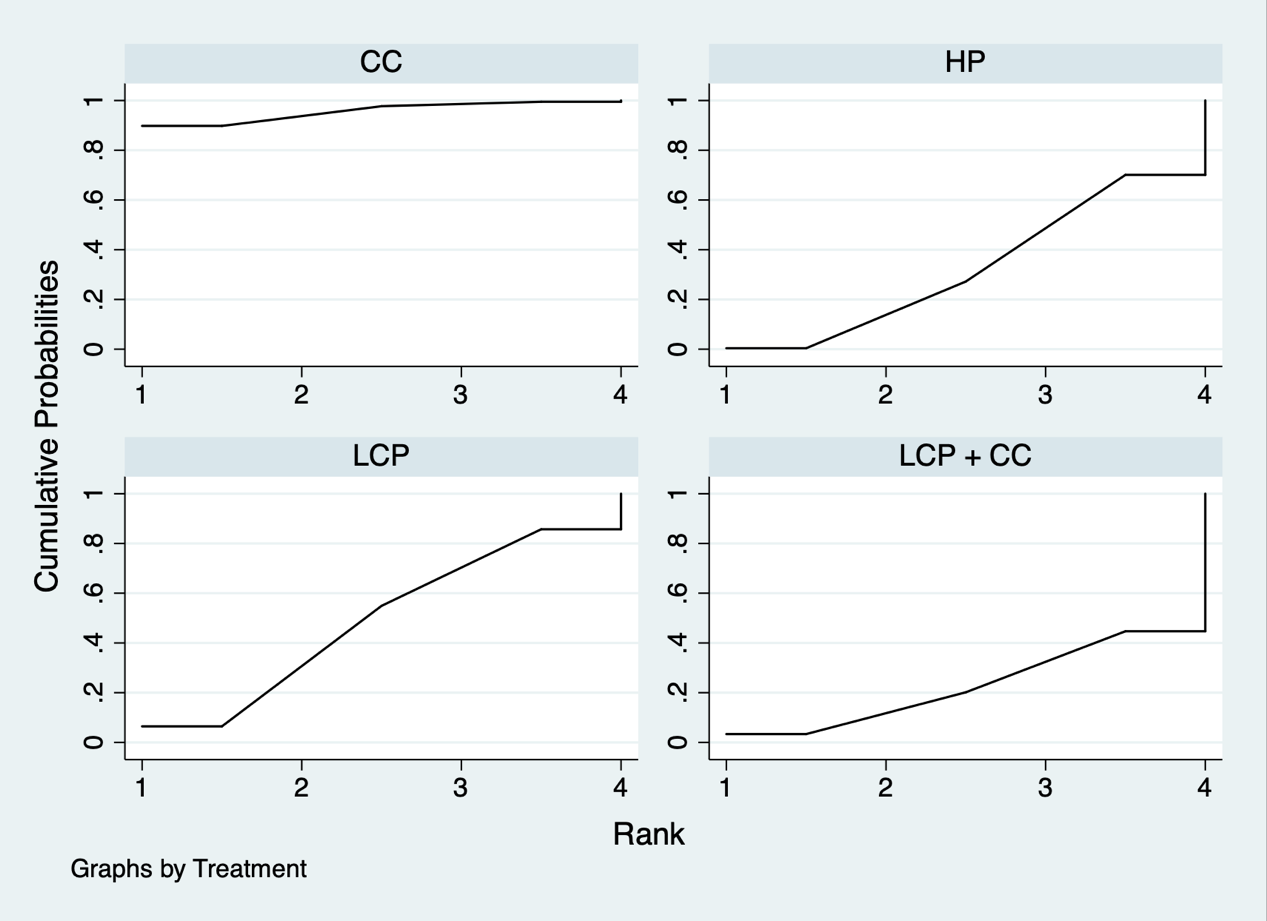
**

**Supplementary Figure 4I**


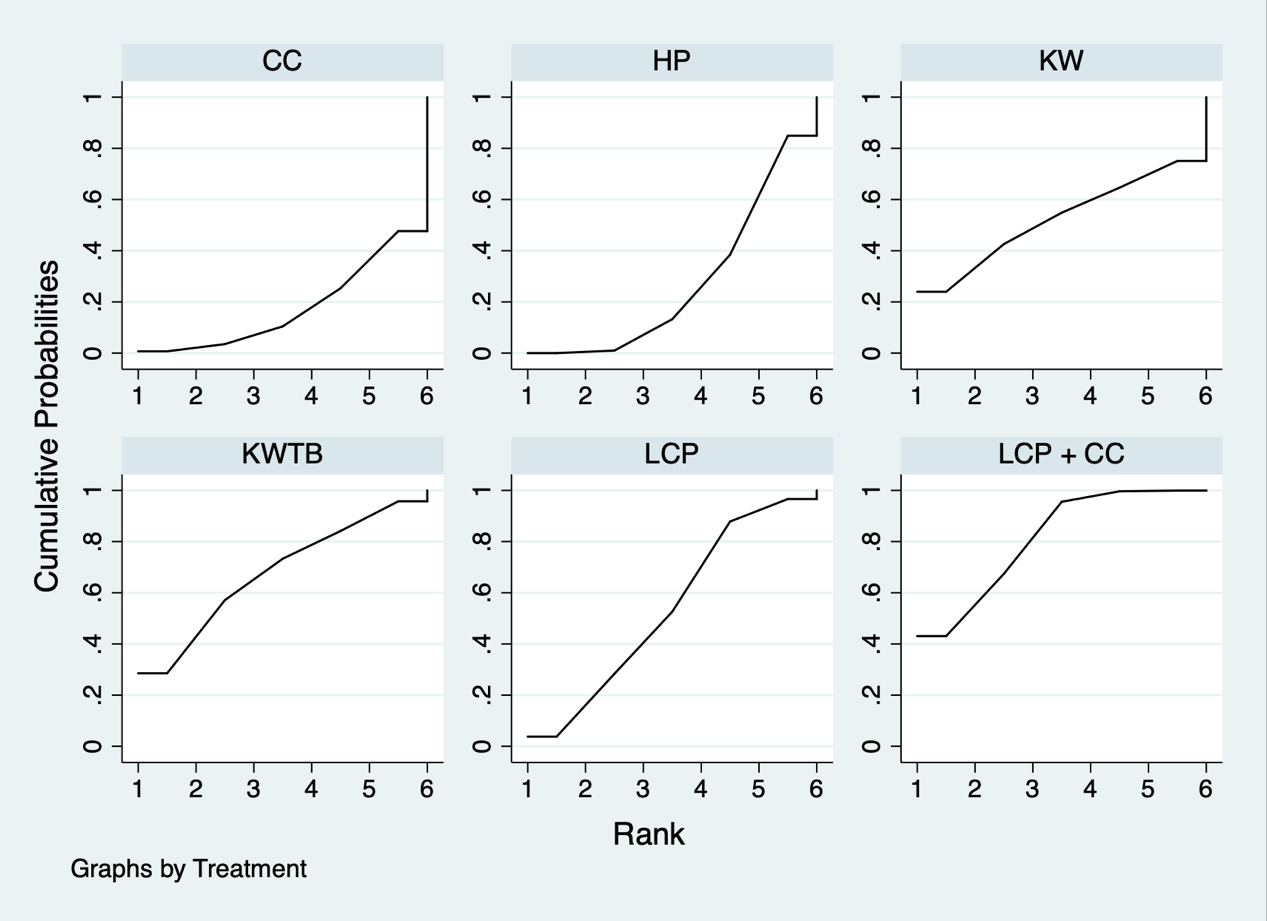

Supplement: Supplementary file 7 — Additional file 7: Fig. S4. Rankogram of different internal fixation methods for outcomes. A. UCLAs; B CCD; C Implant-related complications; D Reoperation; E Nonunion and delayed union; F Incision; G Operative time; H Blood loss; I Union time. [file 13018_2021_2904_MOESM7_ESM.docx]
