# Supplementary material for: Different internal fixation methods for unstable distal clavicle fractures in adults: a systematic review and network meta-analysis
Source: J Orthop Surg Res. 2022 Jan 24;17:43. doi: 10.1186/s13018-021-02904-6 (PMC8785604; doi:10.1186/s13018-021-02904-6)
Supplement: Supplementary file 8 — Additional file 8: Fig. S6. Network meta-analysis funnel plots for the assessment of publication bias of the included studies. A UCLAs; B CCD; C Implant-related complications; D Reoperation; E Nonunion and delayed union; FIncision; G Operative time; H Blood loss; I Union time. A, C–H A: HP, B: LCP, C: CC, D: LCP + CC, E: KWTB, F: KWTB + CC, G: KW; B: A: HP, B: LCP, C: LCP + CC; I A: HP, B: LCP, C: CC, D: LCP + CC, E: KWTB, F: KW. [file 13018_2021_2904_MOESM8_ESM.docx]

**Additional file 8: Figure S6.** Network meta-analysis funnel plots for the assessment of publication bias of the included studies. (A). UCLAs; (B). CCD; (C). Implant-related complications; (D). Reoperation; (E). Nonunion and delayed union; (F). Incision; (G). Operative time; (H). Blood loss; (I). Union time. (A), (C)-(H): A: HP, B: LCP, C: CC, D: LCP + CC, E: KWTB, F: KWTB + CC, G: KW; (B): A: HP, B: LCP, C: LCP + CC; (I): A: HP, B: LCP, C: CC, D: LCP + CC, E: KWTB, F: KW.

**Supplementary Figure 6A**

**
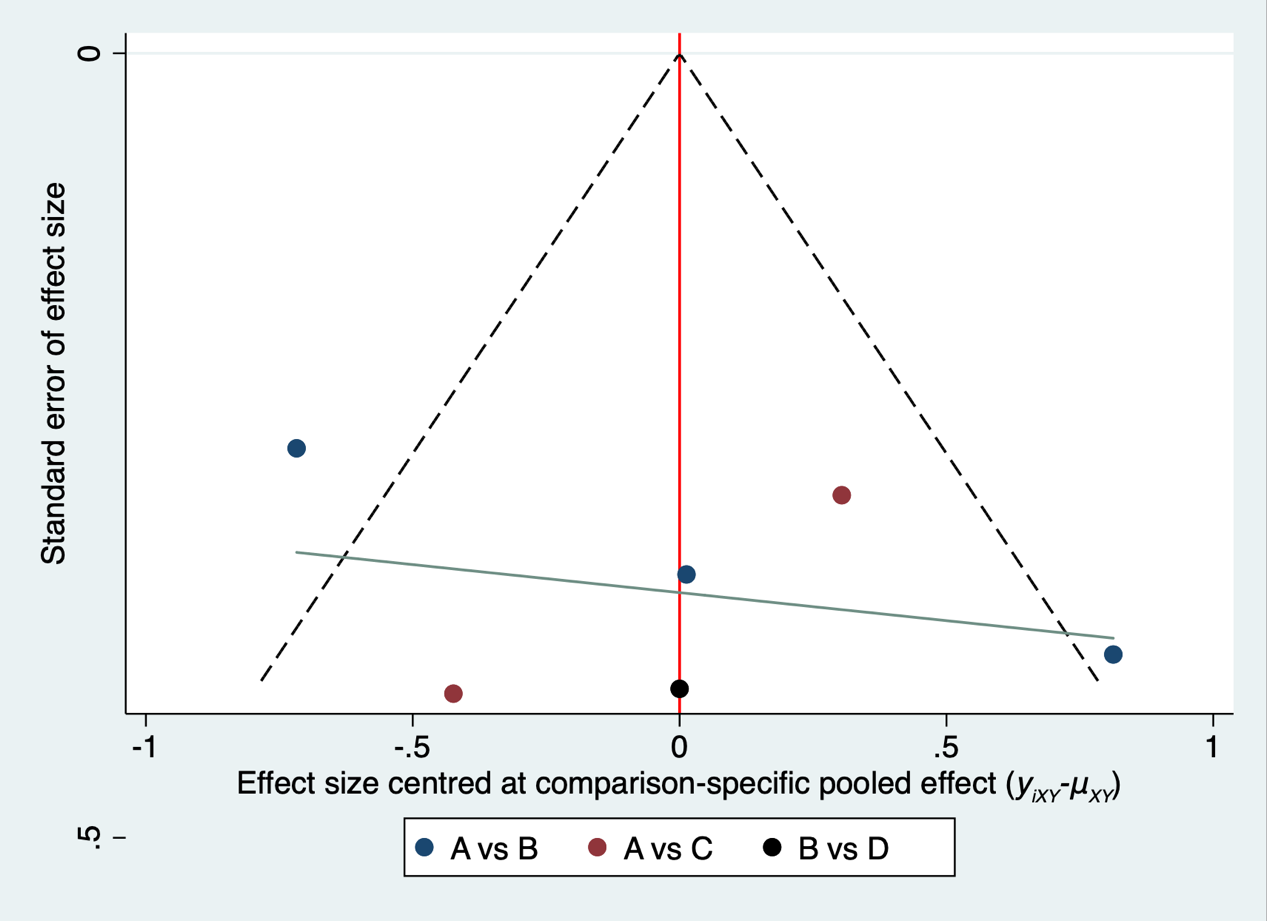
**

**Supplementary Figure 6B**

**
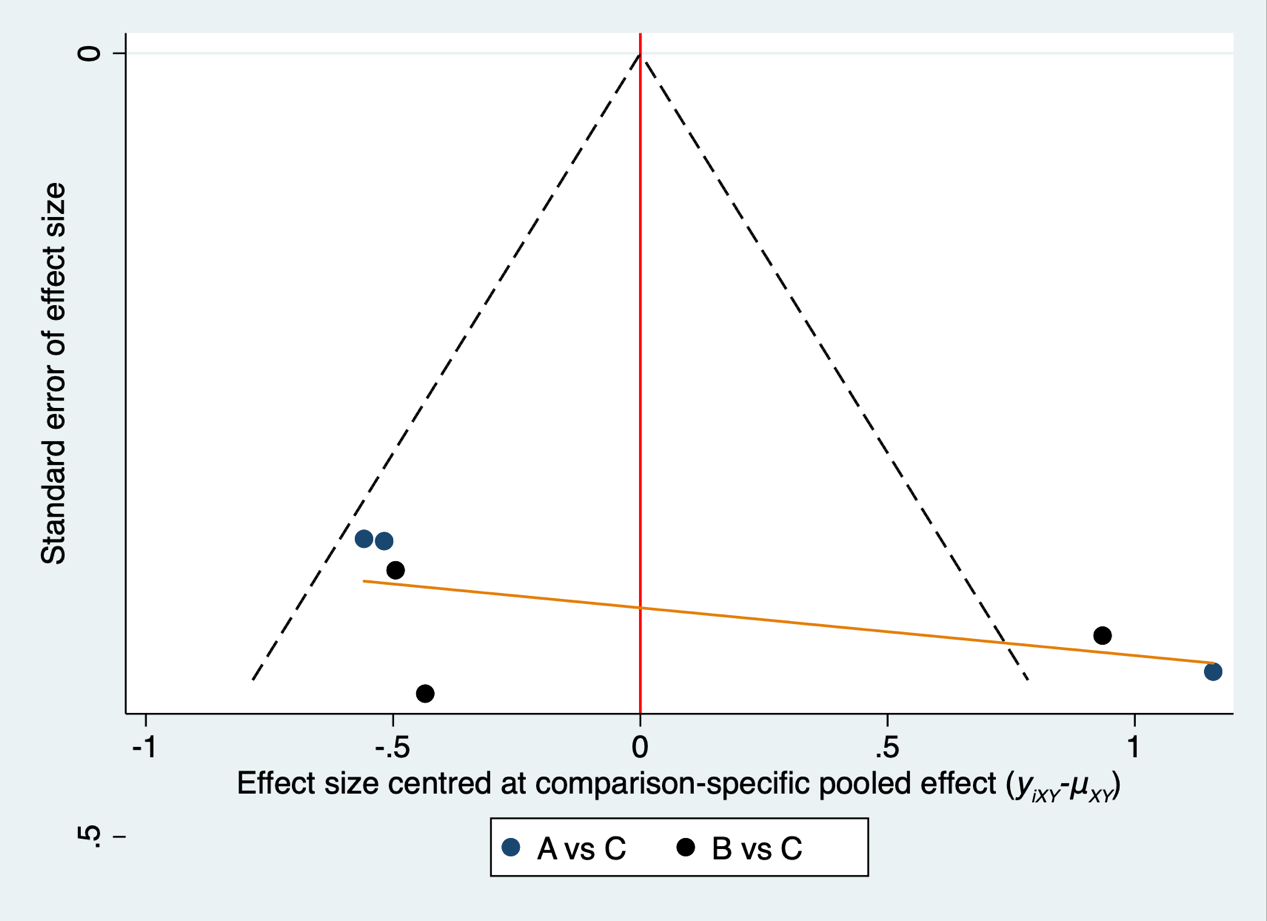
**

**Supplementary Figure 6C**

**
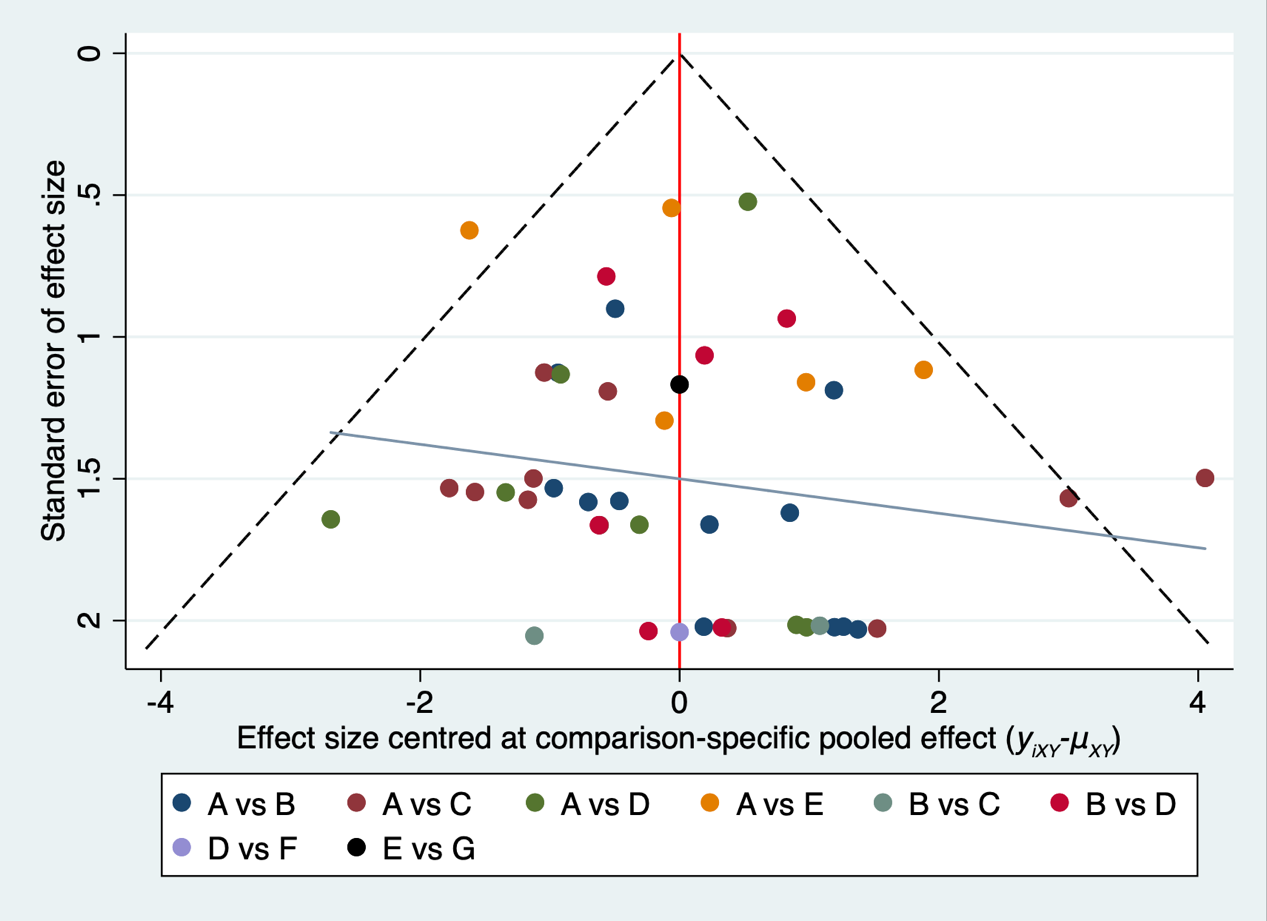
**

**Supplementary Figure 6D**

**
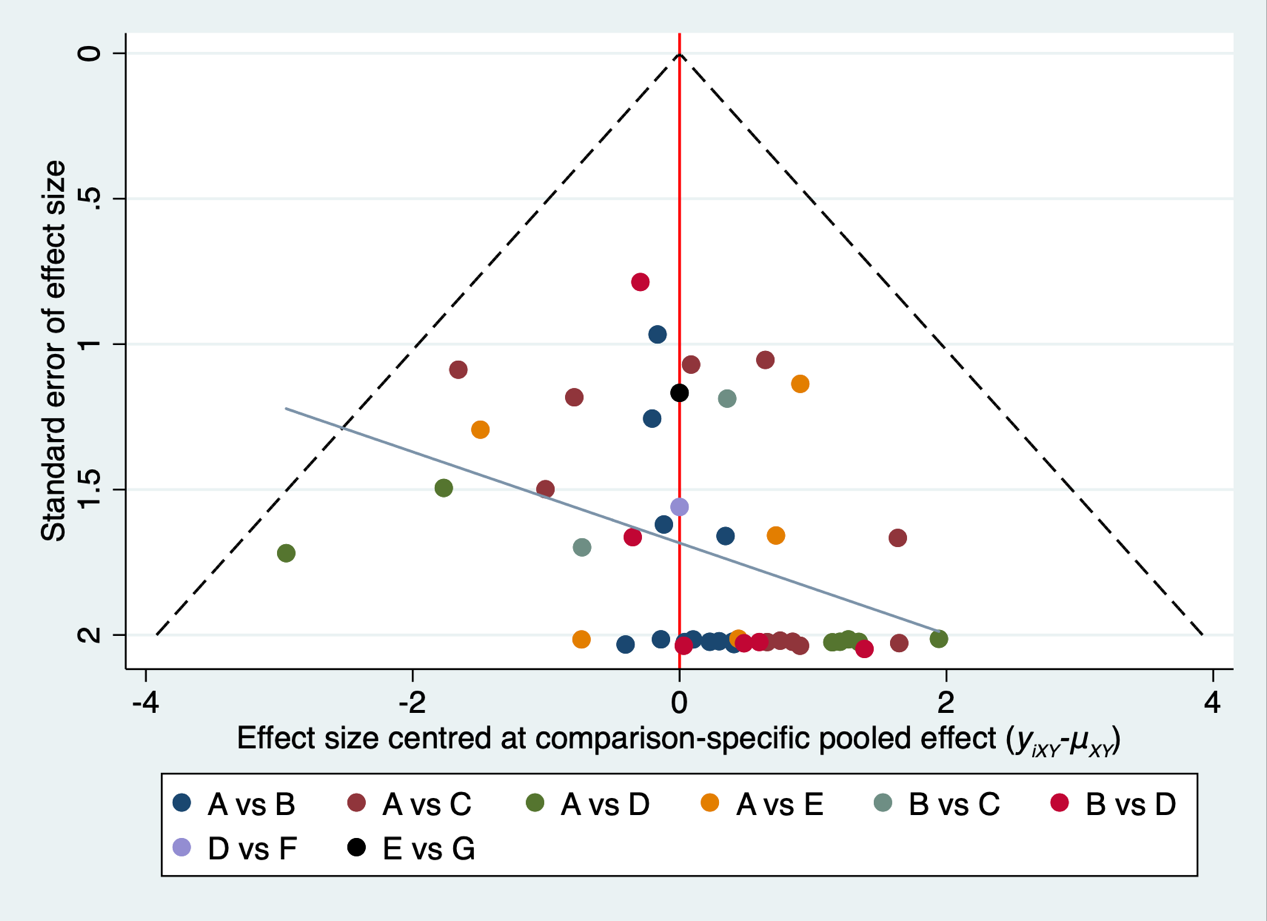
**

**Supplementary Figure 6E**

**
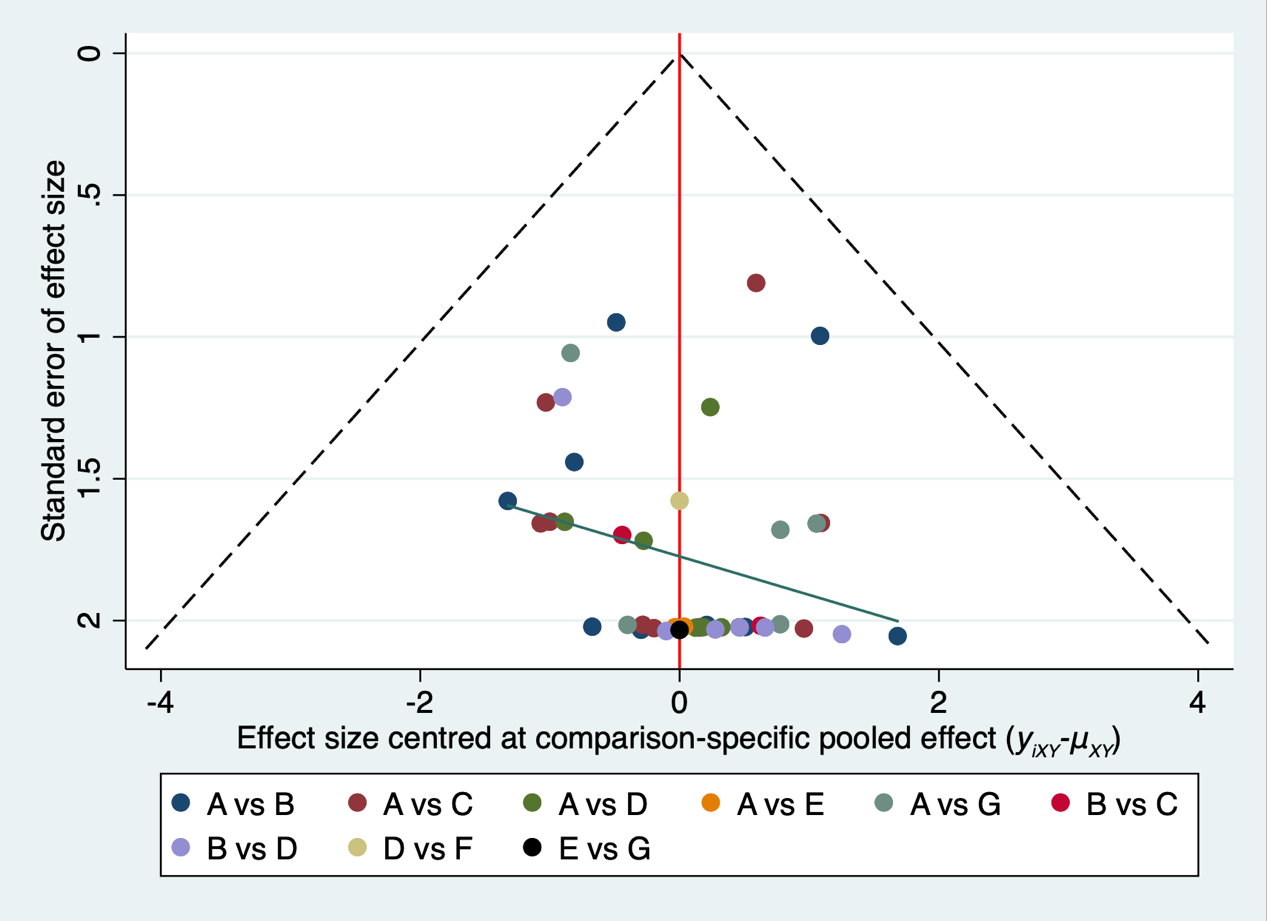
**

**Supplementary Figure 6F**

**
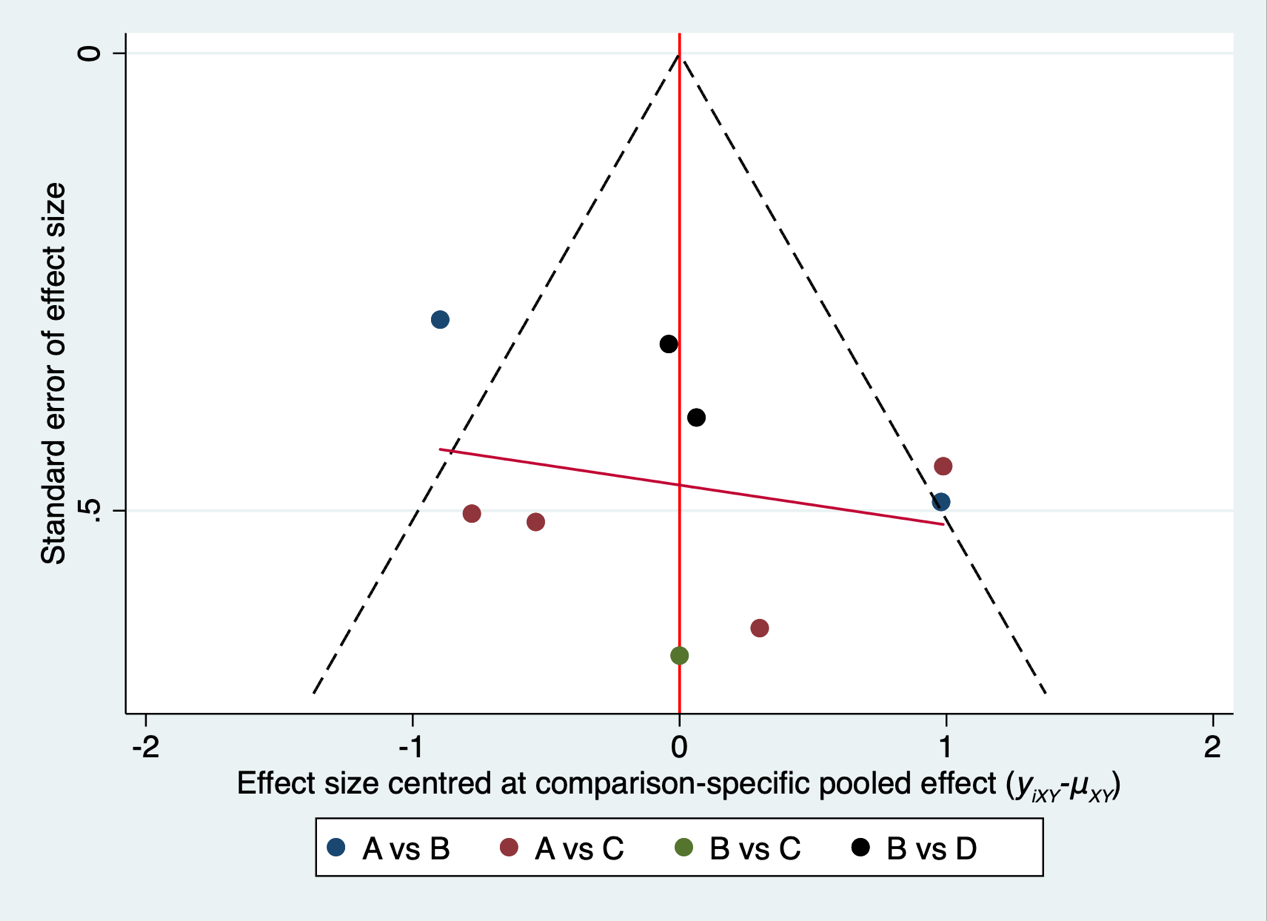
**

**Supplementary Figure 6G**

**
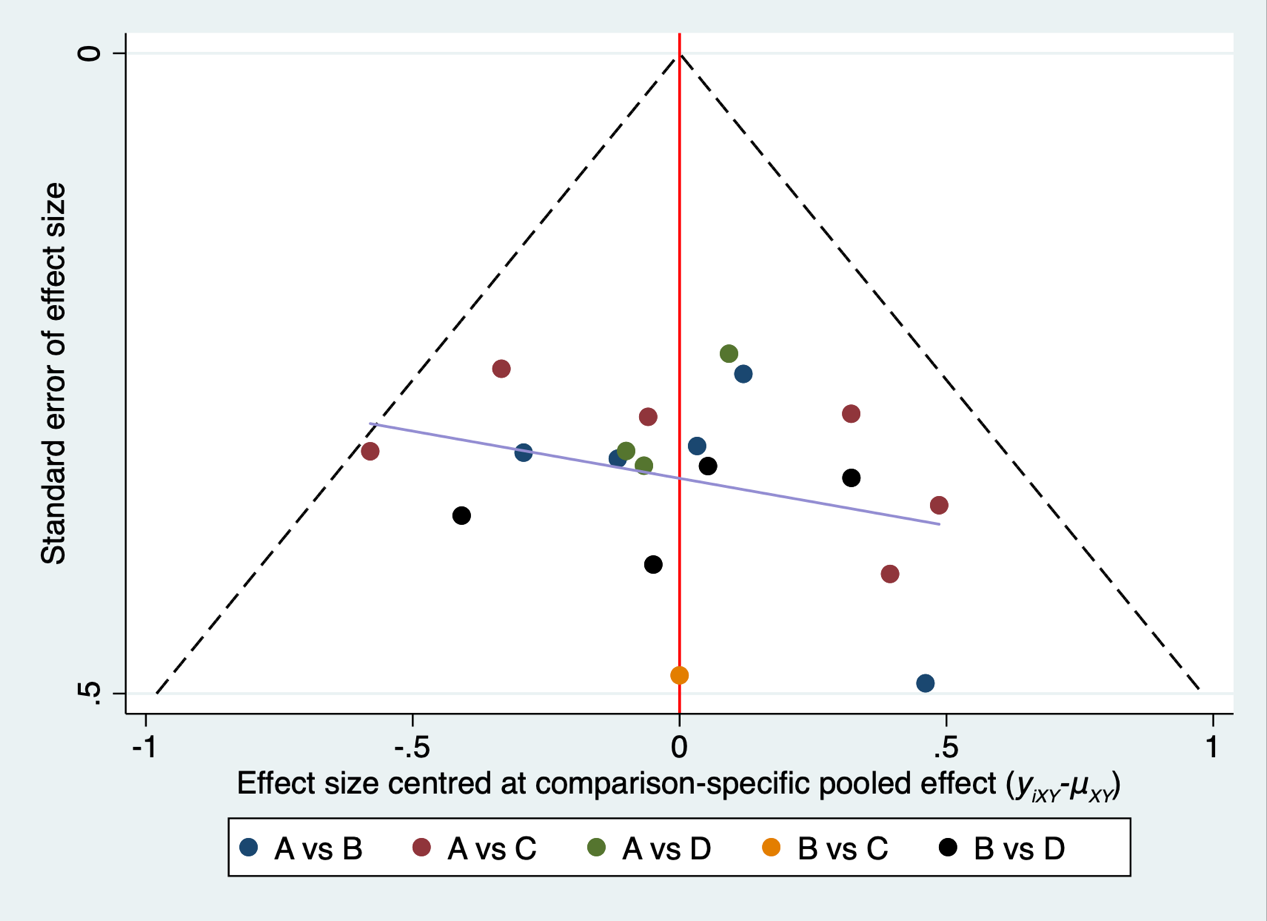
**

**Supplementary Figure 6H**

**
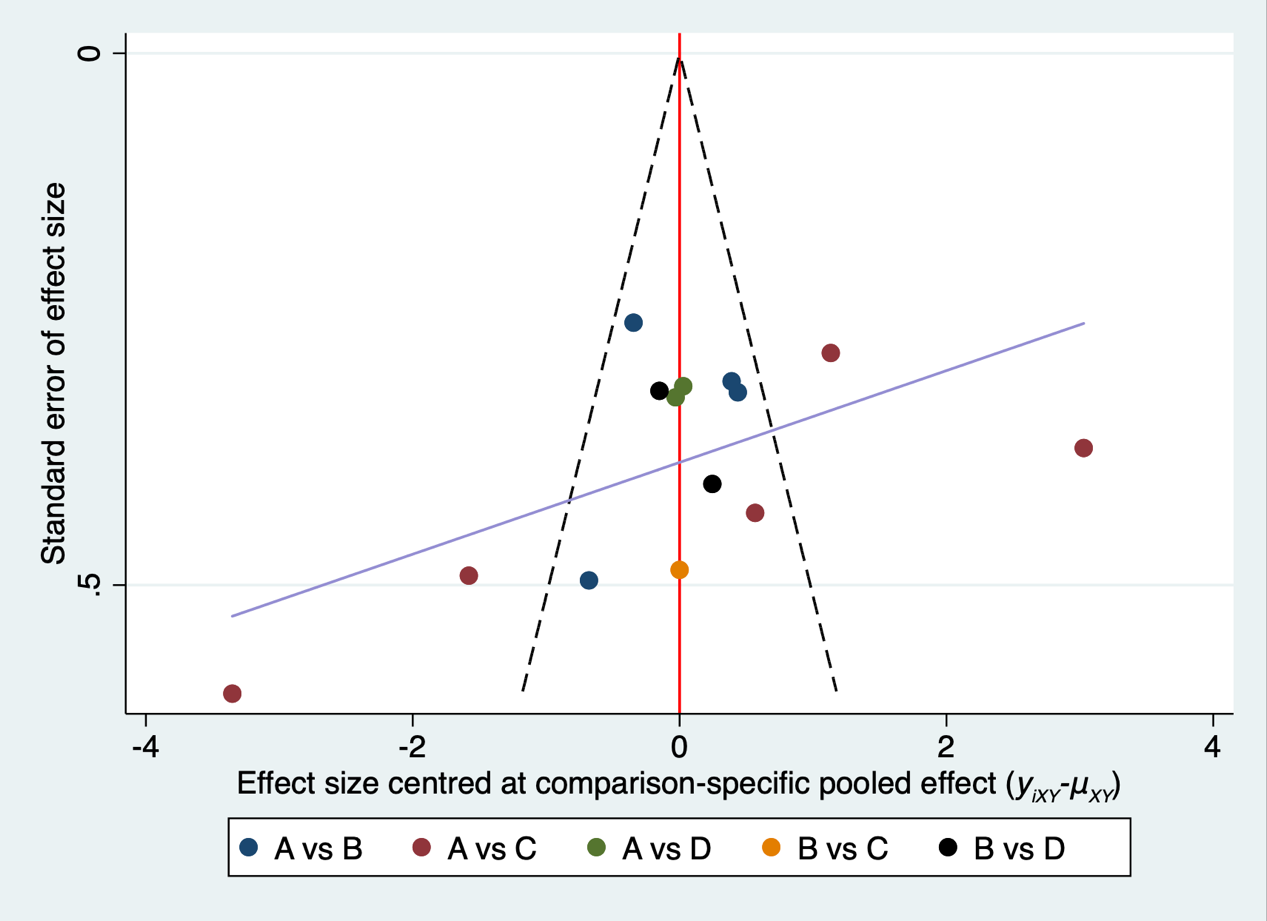
**

**Supplementary Figure 6I**

**
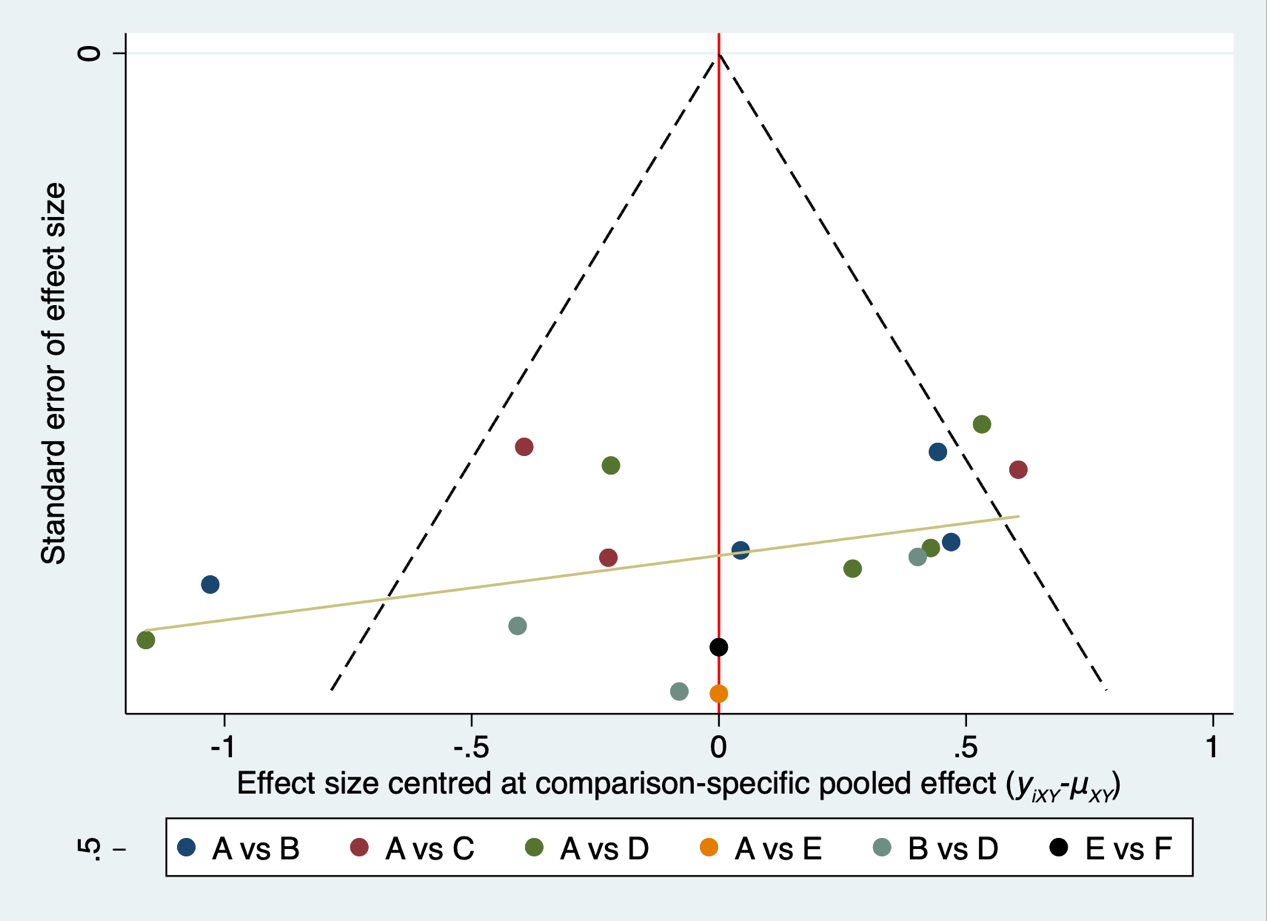
**
